# An ethnopharmacological survey and comparative analysis of plants from the Sudhnoti District, Azad Jammu and Kashmir, Pakistan

**DOI:** 10.1186/s13002-021-00435-2

**Published:** 2021-03-20

**Authors:** Muhammad Faraz Khan, Zia-ur-Rahman Mashwani, Ansar Mehmood, Rehmatullah Qureshi, Rizwan Sarwar, Khawaja Shafique Ahmad, Cassandra L. Quave

**Affiliations:** 1 Department of Botany, Faculty of Basic and Applied Sciences, University of Poonch Rawalakot, Rawalakot, 12350 Pakistan; 2 Department of Botany, PMAS Arid Agriculture University, Rawalpindi, Pakistan; 3 Center for the Study of Human Health, Emory University, 550 Asbury Circle, Atlanta, GA 30322 USA

**Keywords:** Medicinal plants, Ethnomedicine, Pakistan, Sudhnoti

## Abstract

**Background:**

This is the first comprehensive report on the traditional and novel uses of medicinal plants practiced by the indigenous communities of the Sudhnoti district of Azad Jammu and Kashmir (AJK), Pakistan. The area is rich in folklore and indigenous medicinal knowledge due to a unique tribal composition and socioeconomic conditions. This study aimed to document traditional knowledge of native plant use by the local communities, particularly those used for therapeutic purposes.

**Methods:**

Field surveys were conducted from September 2015 to March 2017. Interviews with 125 local inhabitants of different tribes, age groups, genders, and occupations were conducted using structured and semi-structured questions along with group discussions. Data gathered on plant uses, local names, and modes of application of each plant species were organized in tables. Ethnobotanical indices such as use value (UV) and cultural significance index (CSI) were used to produce quantitative information on the plant use category, frequency, and cultural preference of species. Reports on therapeutic uses of medicinal plants were compared with previous studies.

**Results:**

In all, 88 plant species from 45 families were reported, out of which 67 (77%) were used in ethnomedical applications. Asteraceae, Rosaceae, Fabaceae, and Lamiaceae were the dominant families. *Berberis lycium* was the most valued plant species, followed by *Zanthoxylum armatum* and *Taraxacum officinale. Mentha arvensis* had the highest cultural significance, followed by *Mentha longifolia*, *Punica granatum*, and *Zanthoxylum armatum.* Leaves were the most preferred plant parts in the preparation of medicine exclusively or mixed with other parts. The most frequently used process of crude preparation of medicinal plants was cooking. Oral intake was the predominant route of administration.

**Conclusions:**

Our comparative analysis confirmed that most of the plants documented have uses that match those previously reported for the region and other parts of the world, with the exception of novel medicinal uses for 11 plant species, including *Verbascum thapsus* for earache, *Elaeagnus umbellata* for hepatitis, *Achillea millefolium* for oral care, *Dicliptera roxburghiana* to prevent sunstroke in cattle, *Rumex hastatus* for allergy antidote, *Pyrus pashia* for hepatitis, and *Nerium oleander* for diabetes*.*

**Supplementary Information:**

The online version contains supplementary material available at 10.1186/s13002-021-00435-2.

## Background

Azad Jammu and Kashmir (AJ&K) is a predominantly Himalayan state in the north of Pakistan located 90 km away from Islamabad, Pakistan’s capital city. The north-western border of the district is pierced by the Jhelum River from the Rawalpindi district of Pakistan. The area is hilly, striped by narrow valleys along the course of nullahs and streams, nesting a dense rural population of approximately 300,000 people, according to the 2017 census. Studies have shown that the Himalayan region is home to more than 10,000 medicinal and aromatic plants, and local communities primarily rely on herbal medicine to meet their primary healthcare needs [1]. Other studies revealed that more than 80% of the Pakistani population is reliant solely or primarily on traditional medicine for their everyday health and livelihood [2].

Owing to a unique tribal composition, where up to 85% of the district population belongs to the “Sudhan” tribe, the Sudhnoti district has conserved extensive traditional folklore and practices. On the other hand, discrete communities of other ethnic groups, especially the Gujjars, have a legacy of plant uses ranging from food to medicine [3]. The dependence of local communities on traditional medicine can also be linked to poor healthcare infrastructure in the area. The local socioeconomic conditions are characterized by low per capita income compared to other parts of the country, leading to an overwhelming dependence on biodiversity for sustenance and medicine.

Plants are vital for human survival as a source of food, fuel, fodder, timber, and medicine [4]. Recent studies have confirmed large amounts of pollen in fossil spectra from Georgia dating back to the Upper Paleolithic Period [5], indicating the prehistoric relationship of plants with humankind. Ethnobotanical studies aim to document the folk uses of medicinal plants by local communities to understand the traditional interdependence of plants and people. Current approaches in ethnobotany include the scientific study of plant-people relationships for the latter’s well-being in a sustainable manner. The use of relative cultural indices is an emerging trend in ethnobotany, which provides data amenable to hypothesis testing and comparative analysis [6]. Additionally, primary data reported with comprehensive use descriptions and comparative accounts can help rationalize any possible complexity arising from indices [7]. According to a study conducted in the Sudhnoti District, local communities of the area are highly dependent on plants for their therapeutic uses; therefore, documentation of their medicinal knowledge, as well as pharmacological validation thereof, is highly encouraged [8].

Although ethnobotanical studies have been conducted on a large scale in the surrounding areas of AJK [9–12], ethnomedicinal knowledge in the Sudhnoti District remains poorly studied. To the best of our knowledge, a single attempt was made that focused primarily on medicinal plants without acknowledging novel therapeutic uses of medicinal plants [9]. The aim of our study was two-fold: (1) to document the ethnobotanical knowledge of the area and (2) to compare it with other studies conducted in the surrounding regions and other parts of the world at large in efforts to identify novel medicinal plant uses that could merit further pharmacological evaluation.

## Methods

### Study area

**Fig. 1:**
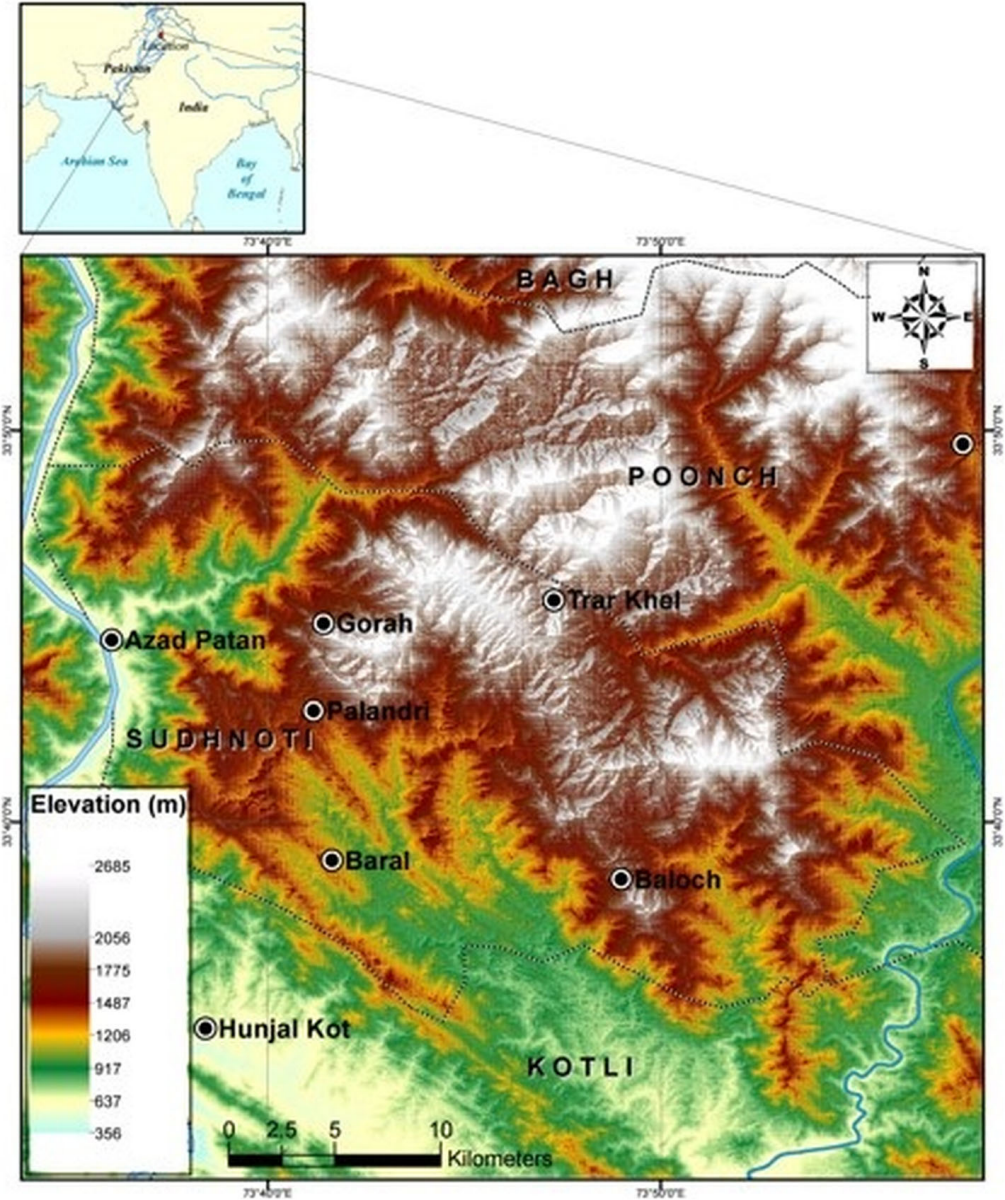
Map of the study area

The Sudhnoti District, AJ&K, study area lies in coordinates ranging from 33^°^.40′–33^°^ 50′ N latitude and 73.40–73^°^ 50′ E longitude. The altitude range is approximately 600 to 2100 m.a.s.l. (Fig. 1). The study area is comprised of hills and mountains along with small valleys and plains, spanning a total area of about 5695 km^2^. During the summer and winter seasons, the temperature ranges between 20 to 35 ^°^C and 5 ^°^C and 20 ^°^C, respectively.

### Ethnographic composition

The study area has a distinct tribal composition with a majority of the population belonging to Sudhan tribe, followed by Awan, Syed, Gujjar, Qureshi, and Kashmiri ethnic groups. Nearly, the entire population lives in a rural setting with a strong religious influence. Hence, gender discrepancies still exist in the health and education sectors and the acquisition of traditional knowledge. When researchers approach a typical rural household, the elder male is most likely the source of traditional knowledge, especially when the researcher is male. In small towns, including the district headquarters, there is an appreciable number of educated youths possessing common knowledge of plant uses. However, the bulk of traditional folklore is still conserved with the illiterate elderly adults, living in the remote highlands and riverside pastures. Besides small-scale conventional farming and livestock rearing, most of the population survives directly on biodiversity and work on daily wages for sustenance. Pahari is the most commonly spoken language in the area, with different dialects spoken. Only a few informants from the Gujjar tribe prefer to speak Gojri while discussing traditional uses of plants; the same is true for elderly women from the Kashmiri tribe. The conventional healthcare infrastructure comprises a district headquarter hospital with a dozen doctors and less than ten basic health units in the entire district. On average, there is one physician per 7000 citizens in the district. Access to physicians is even more restricted for the people living in remote areas as there is a very underdeveloped road network in the study area.

### Sampling and data collection

Six different sites in the Sudhnoti District were selected based on altitude and demographic composition for sampling and data collection, namely, Azad Pattan, Baral, Baloch, Palandri, Gorah, and Trarkhal (Fig. 1). Prior to data collection, a preliminary survey was conducted in which local administration and community stakeholders were engaged for prior consent. Multiple field surveys were conducted from September 2015 to March 2017. Prior informed consent was always verbally obtained before conducting interviews, and the ethical standards of the Society for Economic Botany and International Society of Ethnobiology were followed [13]. Open-ended interviews and structured questions followed field surveys and transect walks either at common places where local people gather (e.g., local bazaars) or at the residences of informants. The first author conducted all interviews in the local language (Pahari) and subsequently documented the interviews in Urdu and English. Local people with biomedical and geographical knowledge were also involved whenever required for better communication, such as understanding disease symptoms. Local healers known as “Pansari,” and nomadic healers locally known as “Sunyasi,” were interviewed with temporarily mounted plant samples.

During fieldwork, 88 plant specimens were collected, preserved, identified, and indexed. A total of 121 informants of 30-85 years old were interviewed (Table 1). The gender division of infomants was 55% male and 45% women. Collected plant specimens were dried, pressed, and mounted on herbarium sheets. The identification of plants was carried out with the assistance of Dr. Rehmatullah Qureshi at PMAS Arid Agriculture University. The Flora of Pakistan [14] was used as a standard for identification and nomenclature. Voucher specimens were submitted to the herbarium of PMAS Arid Agriculture University, and accession numbers were acquired. Botanical nomenclature followed the most updated accepted names included (http://www.theplantlist.org) and family designations followed the Angiosperm Phylogeny Group IV assignments [15]. Digital images of the collected herbarium specimens are available in Additional file 1.

**Table 1:** The demographic composition of the informants

Variables	Categories	No of informants	Percentage
Gender of informants	Male	66	54.77
	Female	55	45.23
Age of informants	25 to 45	26	20.60
	46 to 65	49	39.66
	Above 65	46	37.19
Marital status	Married	95	78.51
	Unmarried	21	17.35
	Widow	5	4.13
Education status	Illiterate	59	48.76
	Secondary school	23	19.00
	College	26	21.48
	University	13	10.74
Employment status	Farmer	27	22.31
	Shepherd	34	28.09
	Other	60	49.58
Ethnic group	Sudhan	82	67.76
	Gujar	19	15.70
	Others	20	30.37
Language	Pahari	114	94.21
	Gojri	4	3.30
	Kashmiri	3	2.47

### Use value (UV)

Use value, a primary measure of all reported uses of a plant species cited by an informant was calculated by the following formula

$$ \mathrm{UV}=\sum {\mathrm{U}}_{\mathrm{i}}/\mathrm{N} $$

where *U*_i_ is the number of uses revealed by each informant for a given species and *N* is the total number of informants.

### Cultural significance index (CSI)

CSI is an anthropological index developed by [16] and modified by [17]. CSI calculates the importance of plant taxon through a researcher-generated weight ranking of multiple factors. A two-point scale for the variables such as use frequency (2 = species effectively used, 1 = not the preferred use), management, and preference were used. To reduce the sensitivity of the method to sampling intensity, a correction factor is also incorporated as in the consensus method [17, 18].

$$ \mathrm{CSI}=\Sigma\ \left(\mathrm{i}\times \mathrm{e}\times \mathrm{c}\right)\times \mathrm{CF} $$

Here, “*i*” refers to the species management, “*e*” is the preference of use of a plant species by informant for a specific purpose, and “*c*” is the frequency of use reported.

### Comparative analysis

Primary ethnobotanical uses of all plant species were compared to regional studies containing “ethnobotany,” “medicinal plants,” “folklore,” or related search terms using different databases [19]. Dissimilar uses cited by other researchers were reviewed and cited. To avoid repetition of similar uses reported in multiple articles for a particular species, the one chosen for citation was the one conducted nearest to the immediate study region.

### Novelty assessment

A total of 13 plant species were identified as being reported for novel uses from the region. These plant species were subjected to a robust comparative review using literature databases, i.e., PubMed, Scifinder, and NAPRALERT, to develop a broader picture of their previously reported pharmacological activities and cross-examining the novelty of uses reported in the present study. Novel uses are highlighted in bold in Table 2.

**Table 2:** Ethnobotanical profile of plants from the area. Plants with novel medicinal uses are highlighted in bold

Family	BN. Acc.	LN	PU	Rec	App	Uses	***N***	UV	CSI	Previously reported uses	References
Acanthaceae	***Dicliptera roxburghiana*** **Nees. FK-5006**		Aerial parts	Fodder	Oral	**Used to treat sun stroke in buffaloes**.	1	0.023	1.70	The powder is used as a general tonic	[20]
	*Justicia adhatoda* L. FK-5022	Bhekar	Leaves	Wrap	Topical	Used with onion, turmeric and oil for papules and pimples.	3	0.103	8.86	Used for wound infections. Decoction is antispasmodic, expectorant and abortifacient	[21]
	***Nerium oleander*** **L. FK-5038**	Kneera	Shoots/flowers	Powder, aerial parts	Oral	**Used to treat diabetes. Used to treat gum bleeding and toothache.**	7	0.139	7.25	Used to treat stomach pain. Used to treat ear pain and eyes problems. Considered poisonous.	[22]
	*Strobilanthes attenuata* Nees FK- 5065	Malol	Flower			Used to prepare local dyes.	1	0.007	2.40	The decoction is used for the treatment of boils. It is also laxative and anti-helminthic.	[23]
Amaranthaceae	*Amaranthus spinosus* L. FK-4986	Ganar	Aerial parts	Vegetable	Oral	Cooked as vegetable Effective against constipation and obesity. Used as fodder.	8	0.103	2.40	Seeds are cooked with rice and given for joint pain.	[24]
Apocynaceae	*Carissa spinarum* L. FK-4995	Granda	Fruit			Fruit is edible	2	0.021	3.00	Fruit is used as a blood purifier.	[25]
Araliaceae	***Hedera helix*** **L. FK-5019**	Batkal	Leaves	Infusion	Oral	**Used to treat diabetes.**	6	0.029	1.60	Used for benign warts	[26]
Asteraceae	***Achillea millefolium*** **L*.*FK-4984**	Kangi	Root	Infusion	Oral	**Root extract is used to treat gum problems. Infusion is used to remove kidney stone.**	4	0.022	3.60	Used for fever, colds, and urinary disorders. Aerial parts are used to improve bile flow.	[27]
	*Artemisia vulgaris* L. FK-4988	Chaoo	Aerial parts	Vegetable	Oral	Cooked as vegetables. Decoction is used for fevers.	6	0.044	2.00	Kills parasitic worms. Leaf extract is used for malaria and fever.	[28]
	*Artemisia absinthium* L. FK-4989	Isanteen	Whole plant	Powder	Oral	Dried ground powder of whole plant is used as hypoglycemic.	8	0.120	4.00	Leaf powder is used for gastric issues. Paste is applied on teeth for pain relief	[28]
	*Cichorium intybus* L. FK-4998	Kasni	Leaves	Vegetable/decoction	Oral	Cooked as a vegetable; decoction is used to treat liver inflammation. Laxative, effective against constipation.		0.059	6.00	Leaves are used as vegetables. Roots and leaves are used as a diuretic, laxative and tonic. Used for fever, gas trouble, and body swelling. Effective for stomach problems.	[29]
	*Conyza canadensis* (L.) Cronquist. FK-5000	Gandi booti				Reported as allergic. Causes skin irritations.	1	0.01	0.24	Uses as fodder	[10]
	*Oplismenus compositus* (L.) P. P.Beauv. FK-5041	Chura kaha	Aerial parts			Fodder grass	4	1.290	1.37	Fodder grass	[22]
	*Taraxacum officinale* F.H. Wigg. FK- 5071	Hand	Leaves	Vegetable	Oral	Cooked as vegetable for women after child birth. Used as antidiabetic and for jaundice.	6	0.260	8.00	Used to treat bone fractures. Used for cattle after delivery for 15 days for strengthening bones and muscles.	[30]
	*Tagetes minuta* L. FK- 5072	Satbarga	Inflorescence	Ointment	Topical	Used as ointments for skin care by females.	4	0.135	2.60	Used for skin disease. Effective against fungal infections. Used in fevers and fits, for piles and earache.	[31]
Berberendiaceae	*Berberis lycium* Royle FK-4992	Sumblo	Root bark	Decoction	Oral	Decoction of root bark is boiled and used for wound healing and as anti-infective.	10	0.163	9.60	Used to treat external injuries and to stop bleeding.	[32]
Boraginaceae	*Cynoglossum lanceolatum* Forssk. FK-5003			Infusion	Oral	Aphrodisiac, demulcent	4	0.140	3.41	Infantile diarrhea, heals sores, wounds, joins cracked and fractured bones and relieves swollen limbs	[33]
	*Trichodesma indicum* (L.) Lehm. FK- 5075		Roots		Oral	Used for inflammation	2	1.331	1.87	Ethno-veterinary uses	[34]
Brassicaceae	*Capsella bursa*-*pastoris* (L.) Medik. FK-4994	Trepatri	Whole plant	Infusion	Oral	Whole plant is used for intestinal problems, i.e. vomiting. Used as fodder.	3	0.014	3.00	Decocotion of the plant is used to lower blood pressure and abdominal pain, bile secretion, obesity and hemorrhages.	[35]
	*Sorghum halepense* FK-5037		Leaves	Vegetable	Oral	Cooked as vegetable for its carminative effect.	2	0.014	6.60	Rhizome is used by “hakeems” for stomach pain and indigestion	[27]
Buxaceae	***Sarcococca saligna*** **(D.Don) Müll. Arg. FK-5059**	Naidroon	Leaves	Infusion	Oral	**Root extract is used to treat toothache**. **Leaves are antipyretic.** Used for thatching	5	0.022	5.0	Used for hypertension, hypoglycemia.	[36]
Convolvulaceae	*Convolvulus arvensis* L. FK-4997		Aerial parts	Vegetable	Oral	Used as fodder.	4	0.088	1.80		
Cypraceae	*Eriophorum comosum* (Wall.) Nees FK-5012	Smakh	Aerial parts			Fodder grass	8	0.074	0.93	Used for abdominal pain care.	[37]
Dryopteridaceae	*Dryopteris ramose* (C. Hope) C. Chr. FK-5008	Leengri	Aerial parts	vegetable	Oral	Used as a vegetable to treat ulcer and constipation.	6	0.037	1.70	Plant juice is used for stomach pain. Fronds are used as fodder for horses.	[38]
Ebenaceae	*Diospyros lotus* L. FK-5007	Amlook	Fruit/ leaves	Food	Oral	Fruit eaten to cure stomach troubles, leaves used as fodder and wood for fuel.	4	0.111	4.80	Juice of unripe fruit in used to lower blood pressure. Fruit is used as a remedy for hiccups.	[20, 38]
Eleagnaceae	***Elaeagnus umbellata*** **Thunb. FK-5009**	Kankooli	Fruit	Juice	Oral	Fruit is edible and fruit juice is taken as a treatment for chronic liver problems including hepatitis.	7	0.177	9.10	Root is edible. Green parts are used as a source of forage. Wood is used for fuel.	[20, 39]
Euphorbiaceae	*Euphorbia helioscopia* L. FK-5011	Dodhal	Aerial parts	Latex	Oral	Used to treat constipation.	7	0.070	3.20	Roots are used as anthelmintic. Aerial parts are used for constipation. Seeds are used for cholera.	[10]
	*Mallotus philippensis* (Lam.) Mill Arg. FK-5029	Kamella	Fruit			Considered toxic due to its purgative effects. Fruit was used to extract red dye previously.	2	0.092	1.80	Anthelminthic effects are reported	[40]
Fabaceae	*Astragalus psilacanthus Boiss.* FK-4990	Kanda	Aerial parts			Used as fodder.	5	0.031	1.13	Leaves are used for flu and toothache.	[28]
	*Indigofera heterantha* Wall Brandis FK-5020	Jand	Aerial parts	Infusion		Used for stomach disorders. Used to make baskets. Used as fodder.	3	0.074	2.50	.	
	*Lespedeza juncea* Wall FK-5023	Jandi	Aerial parts			Fodder grass	4	0.170	0.79	Used to treat a variety of skin diseases	[9]
	*Lotus corniculatus* L. FK-5024	Trepatri	Aerial parts			Fodder	5	0.195	0.69		
	*Melilotus indica* L. All. FK-5033	Sree kaha	Leaves	Vegetable	Oral	Cooked as vegetable. Seeds are used to treat genital infection	1	0.07	2.0	Infusion is used in cough and bronchial disorders.	[3]
	*Trifolium repens* L. FK- 5074	Shatahl	Leaves	Vegetable	Oral	Used to treat gout. Used as fodder.	3	0.037	3.48	Anti-rheumatic and depurative, used in cough, cold, gout and leucorrhoea.	[41]
Fagaceae	*Quercus incana* W. Bartram. FK- 5050	Erruti	Seeds	Powder	Oral	A major source of fuel wood. Seeds are used to treat dysentery. Used to make traditional plough.	3	0.109	1.30	Astringent, used in diarrhea and indigestion, wood is used to make agriculture Tools.	[12, 38]
Gentianaceae	***Gentiana olivieri*** **(Griseb)** **FK-5017**	Neel kanth	Leaves	Decoction	Oral	Decoction is used for cough. **Roots are used to treat infection of genitals in male child.**	6	0.051	7.80	Used for cardiac ailments. Root is used for stomachache	[42]
	*Swertia chirata* Buch.-Ham. ex Wall. FK- 5066	Chraita	Leaves	Infusion	Oral	Used against fevers i.e. typhoid and malarial fevers.	3	0.213	5.40	The decoction is used for the treatment of boils. Laxative and anti-helminthic	[10]
	*Swertia alata* C.B.Clarke. FK- 5067	Chraita	Whole plant	Decoction	Oral	Used for fever and liver inflammation	4	0.201	3.33		
Geraniaceae	*Geranium wallichianum* D. Don ex Sweet FK-5015		Leaves	Paste	Topical	Leaf paste is used for joint pain.	4	0.044	1.60	Root extract is used in chronic diarrhea. Rhizome is used in powder form for backache, mouth ulcers and chronic diarrhea	[43]
	*Geranium nepalense* Sweet FK-5016		Roots	Tea	Oral	Gives relief from pains, i.e. migraine. Used to treat renal disorders.	7	0.022	3.50	Astringent, used to cure renal problems.	[44]
Juglandaceae	*Juglans regia* L. FK- 5021	Khour	Bark	Chewing stick	Oral	Bark is used as chewing sticks to cure gum sores and colorize lips as a traditional cosmetic item.	4	0.150	8.80	Plant is astringent. Used for toothache, bark is used as chew stick. Fruits is edible and sold in market.	[10, 12]
Lamiaceae	*Colebrookea oppositifolia* Lodd. FK-4999	Muskana Bhekar	Leaves	Wrap	Topical	Paste is wrapped on injury site.	3	0.19	2.92	Antibacterial, antifungal	[28]
	*Mentha longifolia* (L.) L. FK-5031	Jangli pondna	Aerial parts	Powder	Oral	Carminative. Used as an ingredient of sausage	5	0.190	9.80	Used against diarrhea. Used as tonic to cough, flu and asthma. Anti-cholera and anti-dyspepsia effects.	[10]
	*Mentha arvensis* L. FK-5032	Podna	Aerial parts	Sauce	Oral	Carminative. Used for its cooling effects; considered good for digestive system.	7	0.99	9.90	Used to cure bronchial disorders and abdominal pain.	[10]
	*Micromeria biflora* (Buch - Ham. ex D. Don) Benth. FK-5035		Roots	Infusion	Oral	Used for a muscular itching of the stomach; locally known as “naar”.	1	0.012	3.80	Used to treat kidney stone. Used as an herbal tea ingredient. Used to treat toothache.	[10, 45, 46]
	*Otostegia limbata* (Benth), Boiss. FK-5040	Chitti sumbal	Aerial parts	Paste	Topical	Used to cure open wound infections	4	0.341	2.48	Antibacterial	[47]
Lilliaceae	*Tulipa stellata* Hook. FK- 5073	Goggar	Bulb			Bulb locally known as “gogger” is edible.	3	0.038	3.50	It has toxic effects on the CNS of animals resulting in high fever, abdominal cramps, violent tremors and twitching of the muscles	[43]
Lythraceae	*Punica granatum* L. FK-5048	Drunni	Fruit peel	Powder	Oral	Fruit is edible, used to prevent infection and to heal wounds.	9	0.111	5.50	Powder made from fruit is used for diabetes and gastric ulcer. Ground rind of dried flower is given for leucorrhea.	[48]
Malvaceae	*Grewia optiva* Drumm. ex Burret FK-5018	Tahamman	Aerial parts	Fodder	Oral	Effective for childbirth in cattle. Fruit is edible. Used to make ropes. Used as forage.	5	0.133	2.20	Used for smooth delivery and quick discharge of afterbirth, given to young animals to induce puberty.	[9]
Moraceae	*Broussonetia papayrifera* (L.) L'Hér. ex Vent. FK-4993	Jangli Toot				Used as fuel and leaves as fodder.	5	0.066	4.20	The plant is toxic and causes allergies. It is also used for fodder and fuel purposes.	[11]
	*Ficus palmata* Forrsk FK-5013	Tussa	Leaf	Ash	Nasal	Fruit is edible. Leaves are used as fodder, wood as fuel. Ash of leaves is sniffed as expectorant.	7	0.190	7.0	Latex is applied on viral warts present on skin. Fruit is used to cure respiratory disorders	[43]
Onagraceae	*Oenothera rosea* L’Her ex Aiton. FK-5039	Nashtar	Whole plant	Infusion	Oral	Used to treat whooping cough and asthma.	3	0.014	2.00	Used to reduce thrombosis, menopause and other degenerative diseases. Plant is used as fodder.	[10, 49]
Oxalidaceae	*Oxalis corniculata* L. FK-5042					Used as fodder	2	0.040	0.98	Reported for antioxidant and antitumor activity.	[50]
Pinaceae	*Cedrus deodara* (Roxb. ex D.Don) G.Don FK-4996					Used as furniture wood.	7	0.148	1.39	Used as anti-inflammatory, analgesic,anti-hyperglycemia, antispasmodic, insecticidal, anti-apoptotic, anti-cancer, immmuno-modulatory, molluscidal, anxiolytic and anticonvulsant properties.	[21]
Plantaginiaceae	*Plantago lanceolata* L. FK-5043	Batti	aerial parts	Dried Husk	Oral	Used for the treatment of constipation and hemorrhoids.	4	0.007	0.10	Used in rheumatism and griping pain of bowels. Astringent. Leaves used in fevers and dysentery and to prevent prolonged bleeding after giving birth. Chopped leaves are used to color skin.	[10, 51–53]
	*Plantago major* L. FK-5044	Salathee	Leaves	Paste	Oral	Used for wound healing and constipation. Used as fodder also	5	0.004	2.00	Used in rheumatism and griping pain of bowels, astringent, leaves. Used in fevers dysentery and to prevent prolonged after birth bleeding.	[51]
Poaceae	*Arundo donax* L. FK-5036	Nal	Whole plant	Pipes		Used to make pipes and thatching material.	4	0.145	4.40		
	*Cynodon dactylon* (L.) Pers. FK-5002		Aerial parts	Paste	Topical	Paste of aerial parts is applied on broken bones for healing	5	0.133	1.20	.	
	*Poa annua* L. FK- 5046	Malla	Aerial parts			Grazed on by cattle.	2	0.034	1.80	Used as fodder	[10]
	*Setaria pumila* (Poir.) Roem & Schult FK- 5064		Whole plant			Used as fodder	2	0.071	0.21	Chewed for toothache and infection. Powder is applied for infection.	[54]
Polygonaceae	***Rumex hastatus*** **D. Don. FK-5056**	Chukhree	Aerial parts	Infusion	Oral	**Used for jaundice and antidote for allergies caused by weeds**.	3	0.051	2.40	Used as antirheumatic, tonic and laxative. Antioxidant, antitumor and antimicrobial Antioxidant, and to cure dental problems.	[55–58]
	*Rumex nepalensis* Spreng FK-5057	Khoh	Aerial parts	Infusion	Oral	Used as antidote for allergic reaction of other weeds. Cooked as vegetable	5	0.074	3.40	Leaf extract is used as antiseptic, cooked as vegetable.	[10]
Pteridaceae	*Adiantum capillus-veneris*. L. FK-4983	Kakwai	Whole plant	Infusion	Oral	Infusion of whole plant is used to treat flu and urinary tract infection (UTI).	6	0.029	2.40	Used to treat bronchitis, hair loss, inflammatory and skin diseases.	[59]
	***Pteris cretica*** **L. FK-5045**	Koochi	Aerial parts			**Used to clean milk utensils as an antifungal agent.**	2	0.083	2.40	The paste wrapped in a bandage is applied on External wounds.	[10]
Ranunculaceae	*Clematis grata* Wall FK-5001		Aerial parts	Decoction	oral	Aqueous extract of aerial parts is used as to treat worms.	6	0.050	6.00	Used to treat intestinal worms in man and cattle.	[32]
	*Ranunculus laetus* Wall. Ex Royle. FK. 5051	Mleeth	aerial parts	Paste	Topical	Used for skin conditions, rashes and burns.	2	0.035	2.40	Paste of fresh leaves is germicidal, applied once a day.	[3]
	*Ranunculus arvensis* L. FK- 5052	Chochumba	Aerial parts	Vegetable	Oral	Used to cure infections Part of a traditional vegetable	2	0.093	1.50	Used to treat gout, fever and asthma.	[23]
Rhamnaceae	*Ziziphus jujube* Mill. FK- 5085	Tukbair	Leaves	Powder	Oral	Considered sacred, leaves and fruit are used as pain healers.		0.091	5.50	Pain healer, gum problems, stomach disorder, heart burn, diarrhea, hemorrhoids, emoilient, skin disease	[60]
Rosaceae	*Fragaria vesca* L. FK-5014	Ammal budha	Fruit	Tea	Oral	Fruit is edible and used to treat heart burn. Diarrhea in children is treated with leaves. Used to make tea.	8	0.125	9.70		
	*Prunus persica* (L.) Batsch FK- 5047	Arwari	Fruit			Fruit is edible, wood is used for fuel.	6	0.186	8.00	Antiseptic, used for external wounds. Used for fuel wood and fodder	[12]
	***Pyrus pashia*** **Buch.-Ham ex D. Don. FK- 5049**	Tangi	Leaves	Decoction	Oral	**Used for hepatitis B and C.** Wood is used in agriculture for tools and as lumber wood.	4	0.191	4.50	Used as forage. Wood is used for making sticks, leaf extract is used for hair loss.	[10]
	*Rosa brunonii* Lindl FK- 5053	Jangli gulab	Leaves	Paste	Oral	Flower is used against scabies. Used as fodder.	4	0.098	5.40	Decoction is taken internally for constipation	[20]
	*Rubus ellipticus* Sm. FK-5054	Akhray				Used for fencing. Leaves are used as fodder. Fruit is edible.	5	0.220	8.80	The fruits is edible and laxative. Used in fencing and hedges.	[61]
	*Rubus fruticosus* L. FK-5055	Pamnar	Fruit/ leaves	Food	Oral	Used for paling. Fruit is used for chilling effect. Leaves used as fodder.	5	0.066	3.50	Fruits are edible and have cooling effect. Spiny branches are used for fencing.	[10]
Rubiaceae	*Rubia manjith* Roxb. ex Fleming FK- 5063	Tharyat				Used as fodder.	2	0.191	1.00		
Rutaceae	*Zanthoxylum armatum* DC. FK- 5084	Timber	Seed/ shoots	Sauce	Oral	Shoot is used to make tooth stick for treatment of oral infections.	5	0.267	8.85	Ground leaves used for digestion. Fruit is carminative and is used in sauce. Fruit is also used for piles.	[10]
Sapindaceae	*Dodonaea viscoa* Jacq. FK-5005	Sanatha	Woody parts			Used as fuel wood and roof thatching in mud houses.	7	0.430	3.80		
Saxifragraceae	*Bergenia stracheyi* Auct*.* FK-4991	Bhatphary	Tuber	Powder	Oral	The plant is used to remove kidney stone. Tuber powder is used for wound healing and diabetes.	3	0.014	4.00	Root extract is used to cure ulcers, coughs and colds. Bark is antiseptic and used to heal cuts and wounds.	[43]
Simaroubaceae	*Ailanthus altissimus* (Mill) Swingle. FK- 4985	Drave	Woody parts			Used for fuel and timber.	6	0.029	3.00	Used for timber and fuel.	[27]
Solanaceae	*Solanum villosum* Mill FK-5060	Kachmach	Fruit/ aerial parts	Vegetable	Oral	Fruit is edible and effective for mouth sores. Cooked as vegetable and used to treat stomachache.	3	0.480	5.40	Fresh leaves are applied Externally for swelling joints. Decoction is drunk for constipation.	[62]
	*Solanum pseudocapsicum* L. FK-5061	Marcholi	Leaves	Paste	Topical	Used to treat boils. Excessive use may cause vomiting.	3	0.014	0.30	Fresh leaves are applied externally for swelling joints. Decoction is drunk for constipation.	[10]
	*Solanum surattense* Burm. f*.* FK- 5062	Mokri	Whole plant	Vegetable	Oral	Cooked as vegetable that is effective for menstrual problems.	2	0.180	0.30		
Ulmaceae	*Ulmus wallichiana* Planch. FK- 5077	Mannu	Leaves			Used as fodder. Wood is used to make furniture and fuel.	4	0.170	4.20	Used as fuel, and for making furniture, branches for fencing and thatching.	[63, 64]
Urticaceae	*Debregeasia longifolia* (Burm.f.) Wedd. FK-5004		Fruit	Food	Oral	Fruit is used to treat bloody diarrhea. Used for fodder and fuel.	3	0.110	4.20	The fruits are grinded and are used against bloody diarrhea. Used as fodder.	[10]
Verbenaceae	***Verbascum thapsus*** **L. FK-5080**	Jangli tmako	Leaves	Wrap	Topical	**Used in combination with mustard oil and salt to treat physical trauma.**	3	0.140	2.20	Leaves are smoked to treat asthma and sore throat. Leaves are applied to inflamed body parts	[65]
	*Verbena officinalis* L. FK- 5079	Choro				Used as fodder.	2	0.037	2.50		
Viburnaceae	*Viburnum grandiflorum* Wall. Ex DC. FK- 5081	Jammar	Fruit/ leavs	Infusion	Oral	Infusion is used to treat typhoid fever. Extract of flower is used to treat respiratory problems.	5	0.207	1.60	Extract of leaves is used for whopping cough and also to relieve abdominal pain. Fruits are used to treat constipation.	[65]
Violaceae	*Viola odorata* L. FK- 5082	Gul naksha	Flower	Decoction	Oral	Decoction has cooling effect. Juice is used to treat earache.	6		7.70	Leaves are taken internally to treat respiratory problems. used in “kahwa” to treat cough and headache.	[61]

Abbreviations: *BN* botanical names and accession number (Acc.), *Fam* family, *LN* local name, *SN* scientific name, *PU* part used, *Re.* recipe, *App.* mode of application, *UV* use value, *CSI* cultural significance index, *N* absolute number of informants

## Results

### Diversity of medicinal plants

**Fig. 2:**
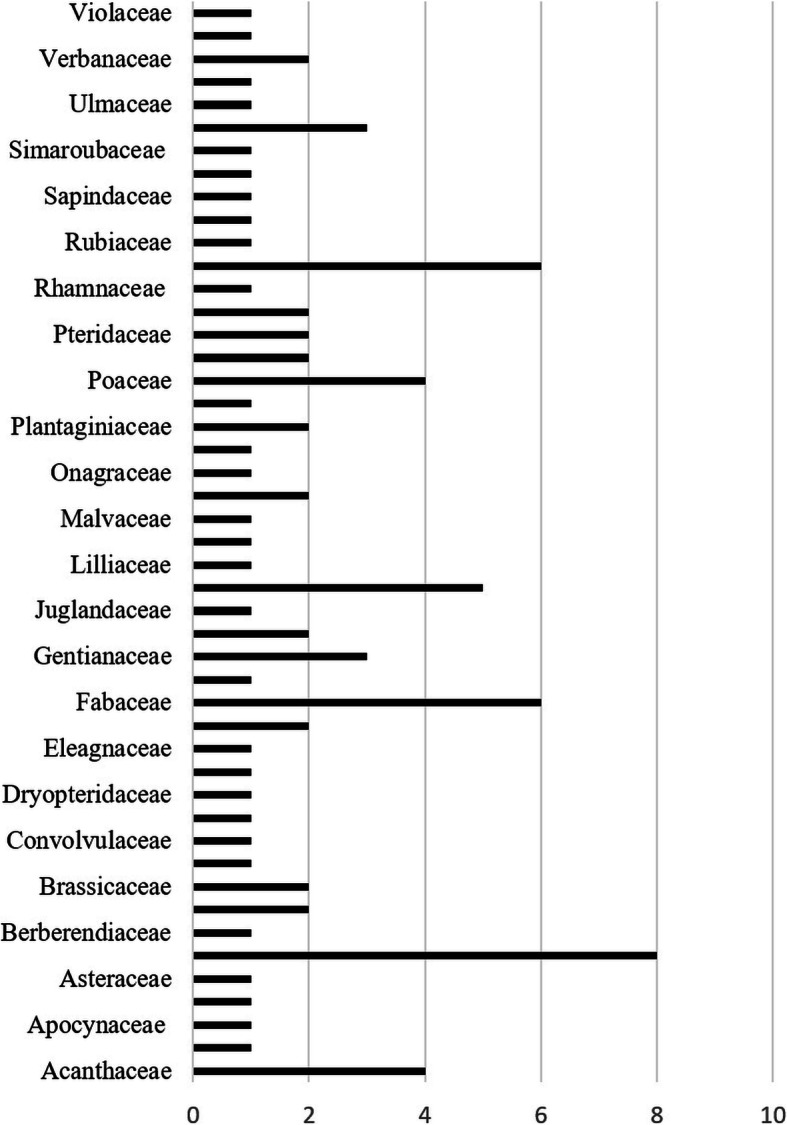
Family distribution of plants used as ethnomedicine among local communities of District Sudhnoti

During the first phase of this study, 88 plant species from 47 families were tabulated after taxonomic identification and scrutiny. Asteraceae was the dominant family with eight plant species, followed by Rosaceae (6), Fabaceae (6), and Lamiaceae (5). Other significant families were Poaceae and Acanthaceae, each with four plant species (Fig. 2). All other plant species represented 45 angiosperm families. The data show that angiosperms contribute to 97% of the collected plant species, followed by ferns (3%).

### Medicinal taxa cited by different ethnic groups

**Fig. 3:**
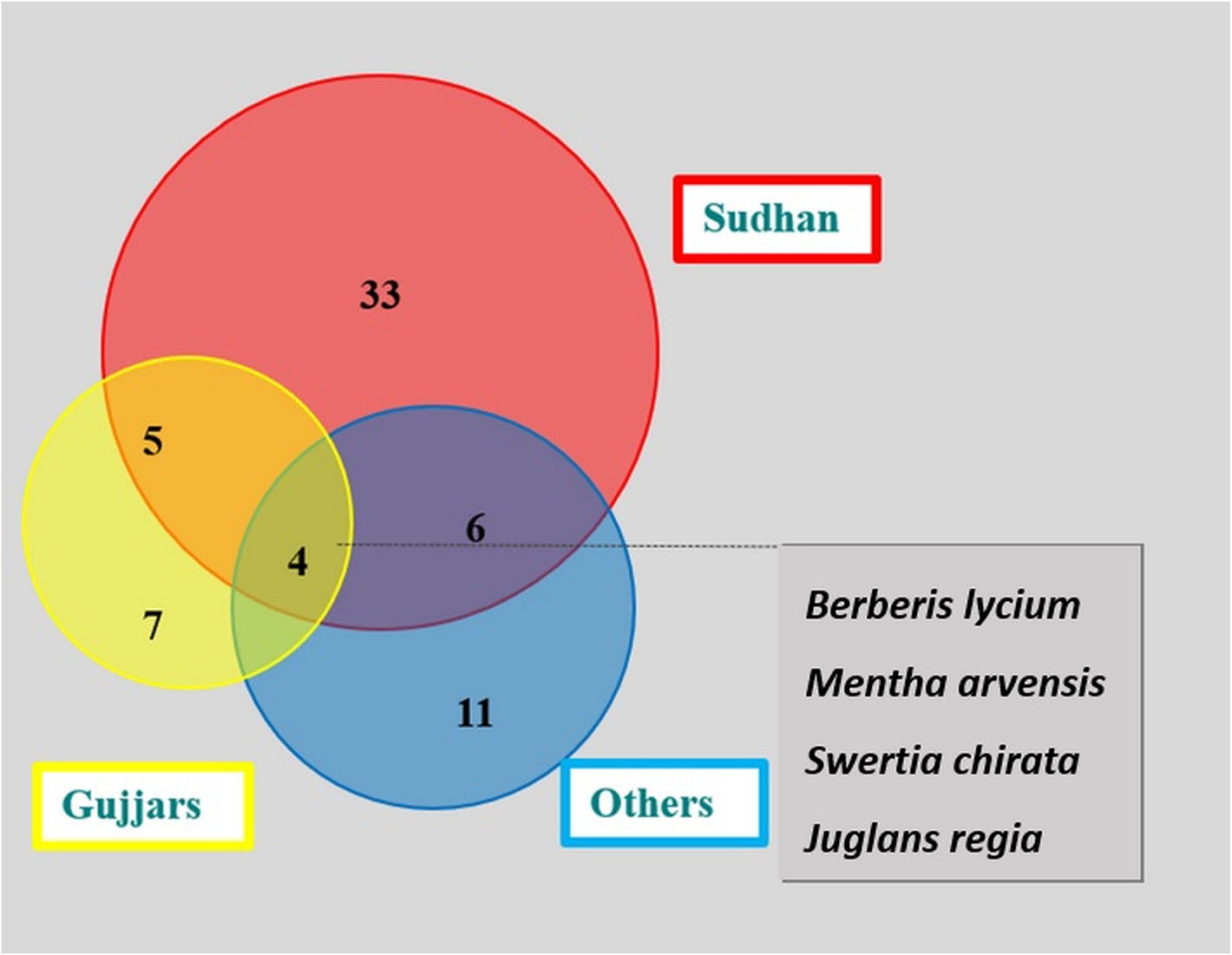
Venn diagram comparing medicinal plants used by different ethnic groups in Sudhnoti District

The informants belonging to different ethnic origins were grouped into three major categories: Sudhan, Gujjar, and Others (Kashmiri, Syed, Quereshit, etc.). Sudhans, owing to their largest share in population, cited the highest number of plants (48), Gujjars cited 21, and others reported 16. There was also an overlap of taxa between ethnic groups: six between Sudhan and Gujjars and five between Sudhan and Others. Only four plants were commonly cited by all three groups (Fig. 3).

### Ethnobotanical uses and therapeutic value

**Fig. 4:**
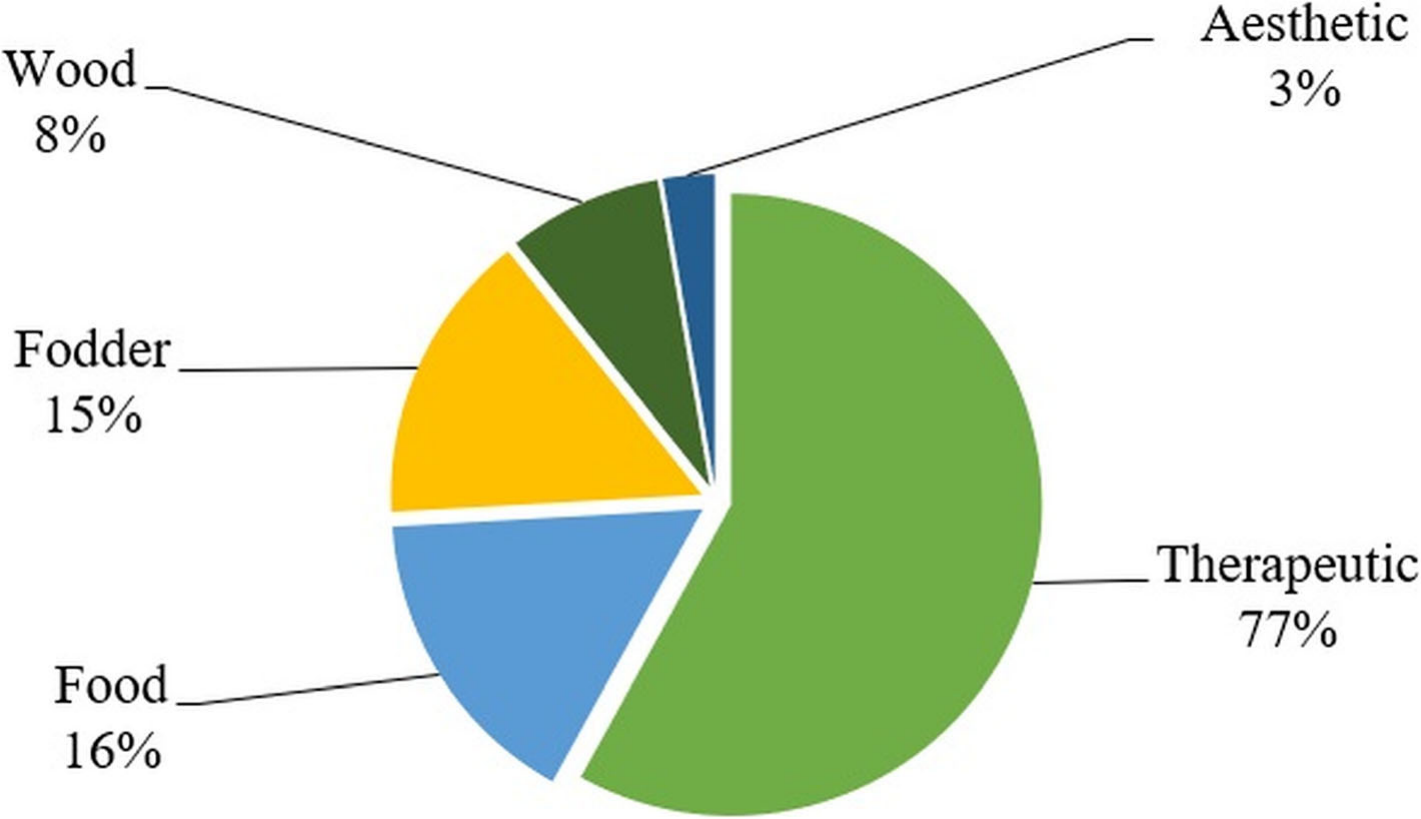
Major plant use categories from the area

Out of the total 88 plant species, 78% of plants were used for therapeutic purposes, followed by food (18%), and wood (8%). Plant species having aesthetic or religious value are grouped together (Fig. 4). The used categories are highly overlapping, as different plant parts of certain species were reported for different uses by different community sections simultaneously.

### Parts used for medicinal purposes

**Fig. 5:**
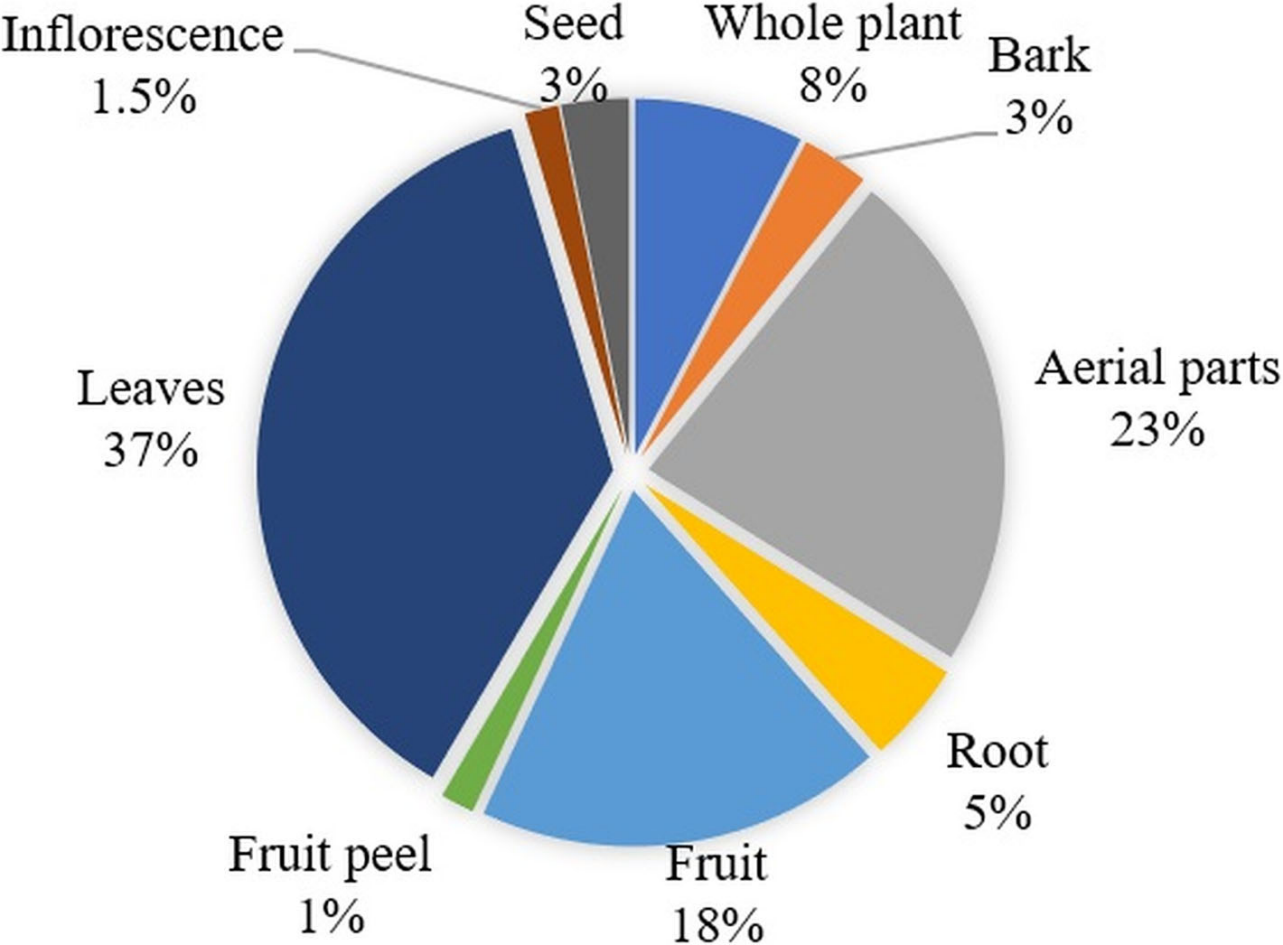
Percentage of plants parts used to cure various ailments

Among the plant species used for medicinal purposes, leaves were the most commonly used plants parts (37%), followed by aerial parts (23%), and fruits (18%). However, in the case of medicinal herbs, the use of aerial parts and the whole plant is dominant (Fig. 5). The use of bark and roots is culturally discouraged due to the local tendency toward resource conservation.

### Method of preparation of folk recipes

**Fig. 6:**
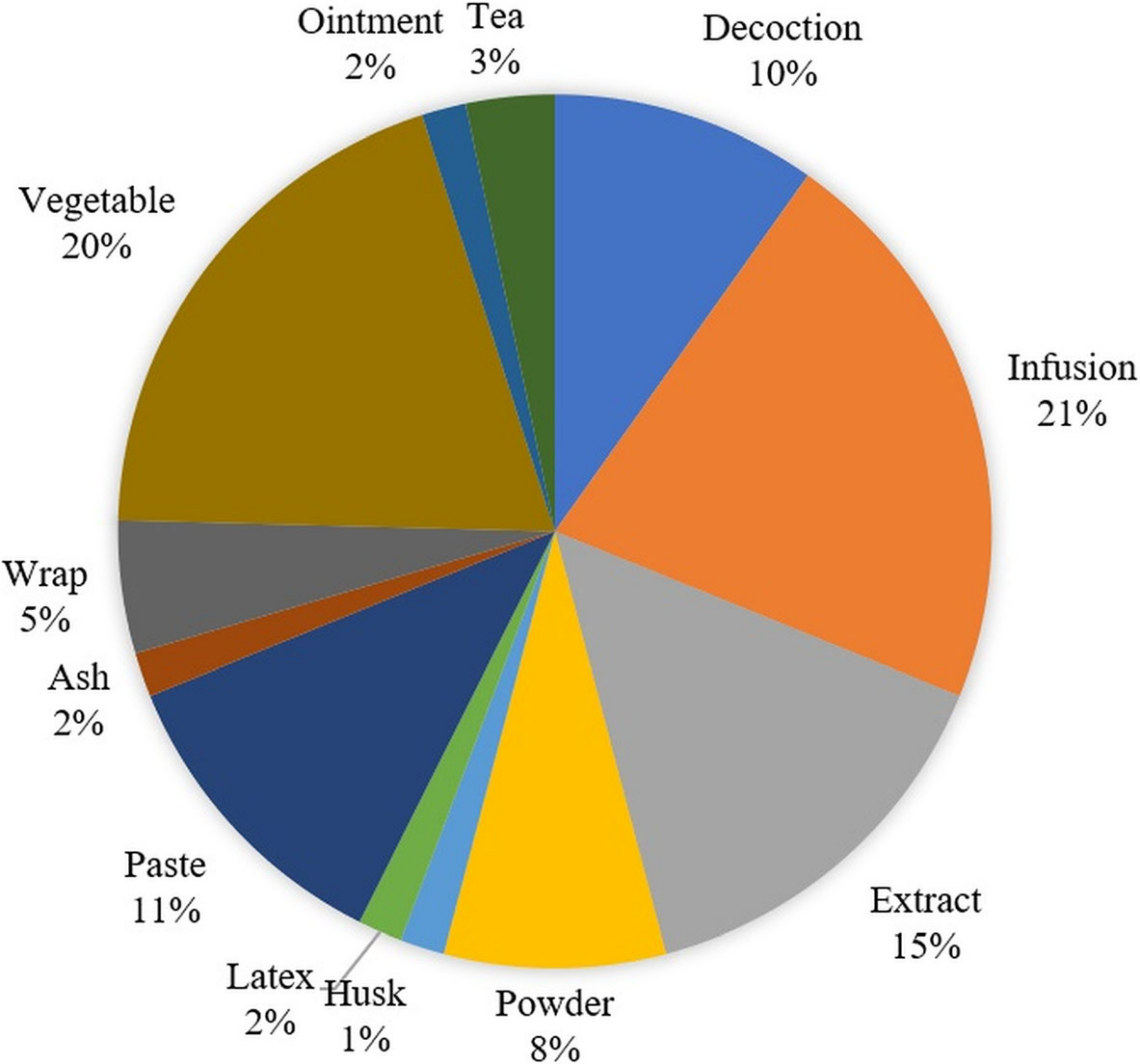
Mode of utilization of plants to treat various diseases

The largest share of plant species (20%) had a reported use as cooked vegetables. However, the share of plants used as infusions (21%) and decoctions/teas (13%) together was 34% of all the species (Fig. 6). Interestingly, there is a notable trend of multi-plant formulations devised by semiprofessional herbalists and traditional practitioners. In these cases, the powder of more than one plant or plant part is orally administered with water in a formula known as “Phakki.” There are some instances with such recipes where overdose and malpractice resulted in adverse drug reactions [66].

### Route of administration

With the ancient mindset that human ailments result from an abnormality in the body’s internal system, most of the botanical preparations are taken orally (85%). External application is restricted to pain management, bone fractures, wounds, and skin infections. A couple of plants were reported for nasal inhalation and as drops for the eyes or ears. The use of certain plant species as chewing sticks (*Zanthoxylum armatum* DC, Rutaceae, and *Nerium oleander* L., Apocynaceae) is also considered internal here for ease of classification.

#### Use value

A total of 20 plant species were identified as the most valuable in the area (Table 2). *Berberis lyceum*, with a use-value of 0.343, was the most valued plant species followed by *Zanthoxylum armatum* (0.28), *Taraxacum officinale* (0.26), *Solanum villosum* (0.26), *Swertia chirata* (0.21), and *Mentha longifolia* (0.1), cited mostly for their medicinal use-value. *Dodonaea viscoa* (0.23), *Pyrus pashia* (0.19), and *Ficus palmata* (0.18) were reported with moderately high use-values.

#### CSI

Cultural significance of a plant species is markedly affected by preference, management, and frequency of use by local inhabitants. *Mentha arvensis*, with a value of 9.9, has the highest cultural significance followed by *Mentha longifolia* (9.7), *Berberis lyceum* (9.6), *Justicia adhatoda* (8.8), and *Zanthxylum armatum* (6.7). Other species with cultural significance are *Punica granatum*, *Artemisia absinthium,* and *Swertia chiraita* (Table 2).

#### Novelty analysis

The current study’s fundamental objective is to compare the findings of the field survey and data analysis with previously reported uses from the region. Comparative analysis identified 11 plant species with specific uses reported from the area for the first time, shown in bold in Table 2. For example, *Elaeagnus umbellata* was reported for liver ailments, *Gentiana olivieri* for genital infection, and *Pteris cretica* for antifungal activity. Pharmacological profiling of plant species was developed with a literature review (Table 2).

## Discussion

The study area has diverse vegetation where 47 plant families represented a field collection of 88 plants. This high level of biodiversity was also reported by a previous study conducted in the area [7]. Altitude variation is one of the factors affecting floral richness in the area, as the study area of a mere few thousand km^2^ represents subtropical (altitude 600 to 2100 m.a.s.l.) to alpine climate conditions. There are rich traditional medicinal practices in the area where 68% of plant species were used for therapeutic purposes. This indicates the presence of a large number of medicinal plants in the local flora and diverse traditional knowledge. Major ethnic groups such as Sudhan and Gujjar prefer to treat their daily ailments with plant-based remedies. The diversity in traditional knowledge is linked to the fact that both of the major tribes have a strikingly different tribal descent where Sudhans originally migrated from Afghanstan still hold strong ties with Pashtoon folklore. On the other hand, Gujjars, originally migrated from central Asia and northeastern Europe, have a legacy of a nomadic lifestyle and remain settled in alpine Himalayan pastures since hundreds of years.

Moreover, District Sudhnoti is one of the most remote districts of Azad Kashmir, having an inferior transport system and road infrastructure. This disadvantage in communication has a conservatory impact on traditional knowledge as an influx of people with other cultural backgrounds is occurs only occasionally. The majority of the study area population lives in far flung rural areas and manage primary health care needs from traditional means. As easy access to hospitals and allopathic medicines is restricted to a mere few thousand people living around the district headquarters of Sudhnoti and a couple of small towns.

Another reason for the strong belief of the local populace on plant-based remedies is due to the influence of traditional healers, who are often adept in the spiritual component of healing. Generally, healers are well-equipped with the concept of Dam Darood (insufflation) and the mythical practices and prayers accompanying adminstration of herbal formulations. Consequently, they prescribe herbal remedies aided by insufflation accompanied by a period of ardent prayers for that particular ailment. For example, elderly women breathe on the roots of *Gentiana oleiveri* and advise it to be used for children with genital infections.

Locals use some plant species due to their medicinal or nutraceutical significance mentioned in Holy scriptures such as Quran and Hadith. For example, *Ficus* is used as an expectorant and *Ziziphus* as a pain reliever because they are mentioned in Holy texts as sacred plants that can cure a range of human ailments. The combination of traditional and spirtual healing is also reported from other parts of the world as well [67, 68].

This study identifies leaves as the most widely used plant part for medicinal purposes. Our findings are in agreement with surveys conducted previously in surrounding areas in the region [32]. The reason for the extensive use of leaves could be their ease of access and having reserves of active metabolites [69, 70]. Here, we found that the preparation of plant-based remedies in infusions and decoctions is the most common form (37%). Technically, an infusion is different from a decoction in a sense that the former is a liquid preparation in which boiling water is poured on the plant material while the latter is the boiling of plant material in the water [71]. Our findings are supported by previous studies conducted in the Poonch region [32].

Use-value is a basic index that accounts for all possible uses of a plant species. A plant with a high use-value index is often more commonly found and frequently used; examples include *Berberis lycium, Zanthoxylum armatum,* and *Taraxacum officinale*. These findings are generally in agreement with previous studies conducted in the are. For example, *Berberis lycium* has previously been reported as the most valued plant species in the area [8]. Herein, we confirm these findings. *B. lycium* was also reported as a high use value plant from Kel village of Neelum Valley, Azad Kashmir, Pakistan, where the local communities used a paste and poultice made from this plant for the treatment of jaundice and diarrhea [72]. Higher use-values often contradict the novelty of plant uses, as the most commonly found plant is more frequently cited and valued by a large number of informants than scarcely found novel plants.

Our methodology discouraged the use of reports on plant species involving agronomic or commercial use-value; this preserved the indices reflection of traditional ethnobotanical knowledge. The cultural significance of a plant considers preference for it, use frequency, and management [18]. *Mentha arvensis* has the highest CSI value despite having fewer citations due to restricted occurrence along the waterways of high temperate areas. Indeed, local people prefer to use and manage *Mentha arvensis* for medicinal and food purposes.

In recent years, plant use information has been compared by using statistical tools such as the Jaccard index. Relevance of these tools is, however, a topic of debate for ethnobotanical studies. It was noted that if plant use information documented from a particular area is subjected to a robust review and comparison with previously published reports on that taxon, its novelty is largely established [73].

The present study reports novel medicinal uses of 11 plant species (Table 2, in bold). These plant species were subjected to a robust pharmacological review from other parts of the world (Table 3). In our study, for example, *A*. *millefolium* is reported for gum soreness and kidney stones. The pharmacological review of this plant shows its application as an antispasmodic, diuretic, urinary antiseptic, and anticancer therapy [74]. *Dicliptera roxburghiana* is given to cattle for the amelioration of “Takko,” an ailment characterized by severe thirst, dehydration, and lethargy. One of the causes of “Takko” as per the general perception of Gujjar (nomadic) tribesmen is the prolonged exposure of cattle to sunlight. Another novel use is the oral intake of *Elaegnus umbellata* fruit for hepatitis B. Informants in our study considered hepatic disorder as a sign of heating up of the liver and stomach. Therefore, they take fruits of *E*. *umbellata* as a cooling agent that potentially has some anti-inflammatory or antiviral effects. The plant is otherwise known for pharmacological activities such as antimicrobial [75], phytotoxic [76], antioxidant, and antiproliferative effects [77]. Ethnomedicinally, it has been reported to treat cardiac problems, bacterial infections, and as an astringent in Malaysia and Pakistan [39].

**Table 3:** Pharmacological activities (review) of the plant species with novel therapeutic uses

Plant name	Pharmacological activities
*Achillea millefolium*	Antispasmaodic, diuretic, urinary antiseptic, antimutagenic, and in the treatment of hyperpigmentation of the skin [74].
*Dicliptera roxburghiana*	Antioxidant [78].
*Elaeagnus umbellata*	Aantimicrobial [75], phytotoxic [76], and antioxidant and antiprofilative [77].
*Gentiana olivieri*	Against epilepsy [79], radical scavanging [80], and immunomodulatory and hepatoprotective [81].
*Hedera helix*	Antibacterial, antifungal and antioxidant [82], anthelmintic [83], anticancer [84], antiviral [85, 86], and antileishmanicidic [87].
*Nerium oleander*	Antibacterial and anticancer [88].
*Pteris cretica*	Antimicrobial and antioxidant activities [89].
*Pyrus pashia*	Antimicrobial and antioxidant
*Sarcococca saligna*	Antimicrobial, antispasmodic [90], hypertension [36], and cardiosuressant and vassodilator [91].
*Rumex hastatus*	Antipyretic and antiinflammatory [53].
*Verbascum thapsus*	Antiviral [92–94] and anticancer [95].

*Gentiana olivieri* is another plant reported here for a novel use that reduces genital infections and inflammation in male children. The symptoms of this condition include inflammation of the testicles followed by itching and soreness. Fresh flowers are directly squeezed into the mouth in the form of drops as an oral dosage. Additionally, the dried root segments are enveloped in a piece of cloth and advised to be worn around the neck for spiritual healing effects. Previously, it has been reported for hepatoprotective activities in Turkish folk medicine [96]. Pharmacological applications against epilepsy [79], for radical scavenging [80], and as immunomodulatory and hepatoprotective agent [81] have also been reported.

An infusion made from dried leaves of *Hedera helix* is used to treat diabetes. Previously, it has been reported for treating benign warts [25]. Pharmacologically, it has antibacterial, antifungal, antioxidant [82], anthelmintic [83], anticancer [84], antiviral [85, 86], and antileishmanial [87] activities.

*Nerium oleander*, a well-known plant for its poisonous effects due to the presence of cardiac glycosides, was reported for diabetes and oral hygiene. In the traditional treatment of diabetes, leaf powder is mixed with the powder of *Stewia* species to neutralize its toxicity and improve the taste. A young shoot is used as a chewing stick (Miswak) to cure gum infections. Previously, *N. oleander* has been reported for stomach and earache [22]. Anticancer and antimicrobial activities have been reported for this species [88].

*Pteris cretica*, a fern, is locally used to clean the milk utensils. It is considered useful for reducing foul odors, but it is also believed that the plant species is a cleansing agent purging milk utensils in a religious context. It is important to note that milk (particularly cow’s milk) is considered a holy food item in Hindu culture. Hindus have long inhabited the study area, and therefore, imprints of traditional Indian culture are still evident. Previously, this species has been reported for wound healing applications [10], and it exhibits antimicrobial, antioxidant [89], anti-inflammatory, and anticancer activities [97].

*Pyrus pashia* is one of many wild fruit yielding species of Rosaceae family. Young apices (with leaflets) of the plant are ground, and the aqueous extract is taken orally for hepatitis B and C, colloquially known as “black jaundice.” In previous studies, the plant was reported for the treatment of baldness [10]. In vitro bioactivities, such as antimicrobial and antioxidant, have also been reported [89].

*Sarcocca saligna* locally known as “naidroon” is another species reported here for applications in treating toothache and fevers. Roots of the plant are crushed in water, and the filtered extract is used in the form of drops onto the affected teeth. In fever, particularly one that of enteric origin (Typhoid), the extract is diluted and taken orally. Previously the plant has been reported for treating hypertension [36]. Pharmacological reports include antimicrobial, antispasmodic [90], cardio suppressant, vasodilatory [91], and hyperglycemic effects [98].

A leaf infusion of *Rumax hastatus* is reported here for liver ailments, particularly jaundice, owing to its cooling effect. Local healers consider jaundice a sign of an “internal heating” and a forthcoming indication of chronic hepatic disorders. Therefore, *R. hastatus* is taken as a chilling agent that “cools down” the body and helps the liver to alleviate the damage. The plant is also reported as an antidote for allergic reactions to weeds such as *Parthenium hysterophorus*. Here, fresh leaves of *R. hastatus* are reported to be rubbed on the affected body parts. *R*. *hastatus* has been previously reported for antirheumatic activity, as a tonic, laxative [55], and as an antioxidant, antitumor, and antimicrobial agent [56, 57].

Leaves of *Verbascum thapsus* are reported here for healing physical trauma; briefly, the fresh leaf is coated with salt sprinkles and wrapped around the injury site to as an analgesic and to heal contusions. *V*. *thapsus* is commonly known as a wild form of tobacco and smoked at times with a recreational sense. It has also been reported in the region for asthma, sore throat, and inflammation [65]. It is also reported to have anti-lice and flea activity and used for earache [99]. Pharmacological evaluation has consistently established antiviral activities of *V*. *thapsus* [92–94]. The plant is also reported for anticancer activity [95].

On the other hand, uses reported from other parts of the world are similar to what we report here, such as the antimalarial use of *Melia azedarach * [45, 46], indicating a global consensus. This phenomenon allows for different cultures to learn from each other about further medicinal uses of their plants and reveals to researchers more bioactivities that some phytochemicals may exerte. Lastly, we conclude that the new traditional ethnopharmacological applications reported here for 11 plant species should be pursued within the framework of ethnobotanical drug discovery. Significant antibacterial activity of plant species including *Z. armatum*, *A. capillus venaris*, *M. annua*, and *A*. *absintium* has already been reported by our research group [100].

## Conclusions

This is the first detailed ethnobotanical study conducted in District Sudhnoti of Azad Kashmir. A total of 88 plants belonging to 47 families were reported, verifying the high biodiversity of medicinal species in Sudhnoti and, therefore, the need to preserve it. Moreover, 11 plant species were found to have novel uses not reported elsewhere. Information concerning the ethnomedicinal uses of plants, specific plant parts, and application methods is critical to both the preservation of traditional knowledge and provides a basis for future drug discovery activities. In this context, the current study enriches our knowledge basis of the medicinal plant potential and traditional knowledge of District Sudhnoti. Future ethnobotanical and phytochemical research will be fundamental to edxploring the pharmacological potential of these and other botanical traditions.

## Supplementary Information

**Additional file 1.** Digital images of the collected herbarium specimens.

## Data Availability

All data generated or analyzed during this study are included in this published article and its supplementary information files.
