# Supplementary material for: An ethnopharmacological survey and comparative analysis of plants from the Sudhnoti District, Azad Jammu and Kashmir, Pakistan
Source: J Ethnobiol Ethnomed. 2021 Mar 20;17:14. doi: 10.1186/s13002-021-00435-2 (PMC7980561; doi:10.1186/s13002-021-00435-2)
Supplement: Supplementary file 1 — Additional file 1. Digital images of the collected herbarium specimens. [file 13002_2021_435_MOESM1_ESM.pdf]

EMORY UNIVERSITY
022194
HERBARIUM

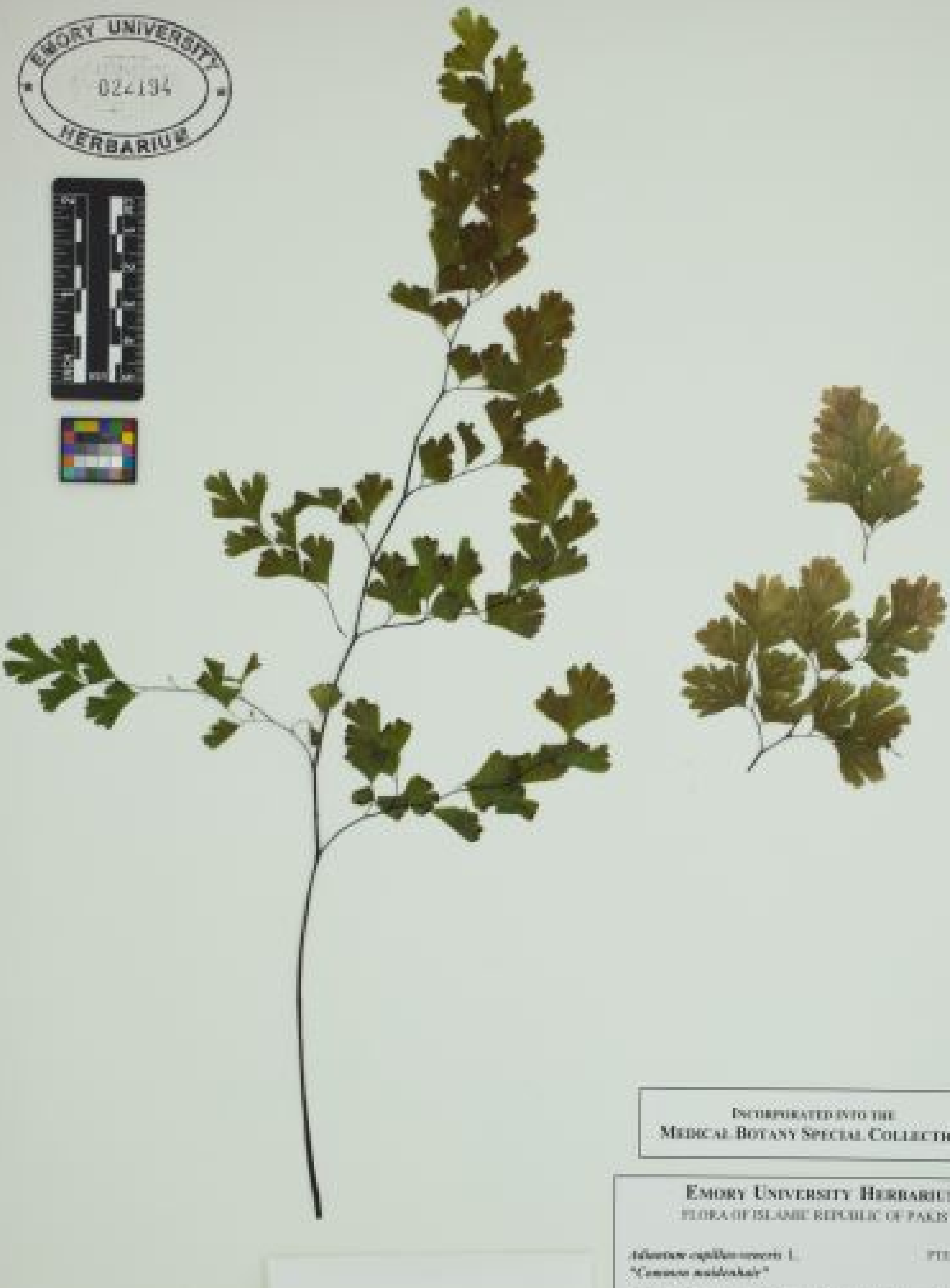

INCORPORATED INTO THE
MEDICAL BOTANY SPECIAL COLLECTION

**EMORY UNIVERSITY HERBARIUM**
FLORA OF ISLAMIC REPUBLIC OF PAKISTAN

*Adiantum capillus-veneris* L. PTERIDACEAE
*"Common maidenhair"*

Garidu, Sudhanoti, Azad Kashmir, Islamic Republic of Pakistan.

shady

shiny, glabrous

Fronds are ground and used to treat urinary tract infection common name: Kakwai

Muhammad Faraz Khan FK-108 6 June 2017

T.K. 108

Michelle Lim 25 September 2017

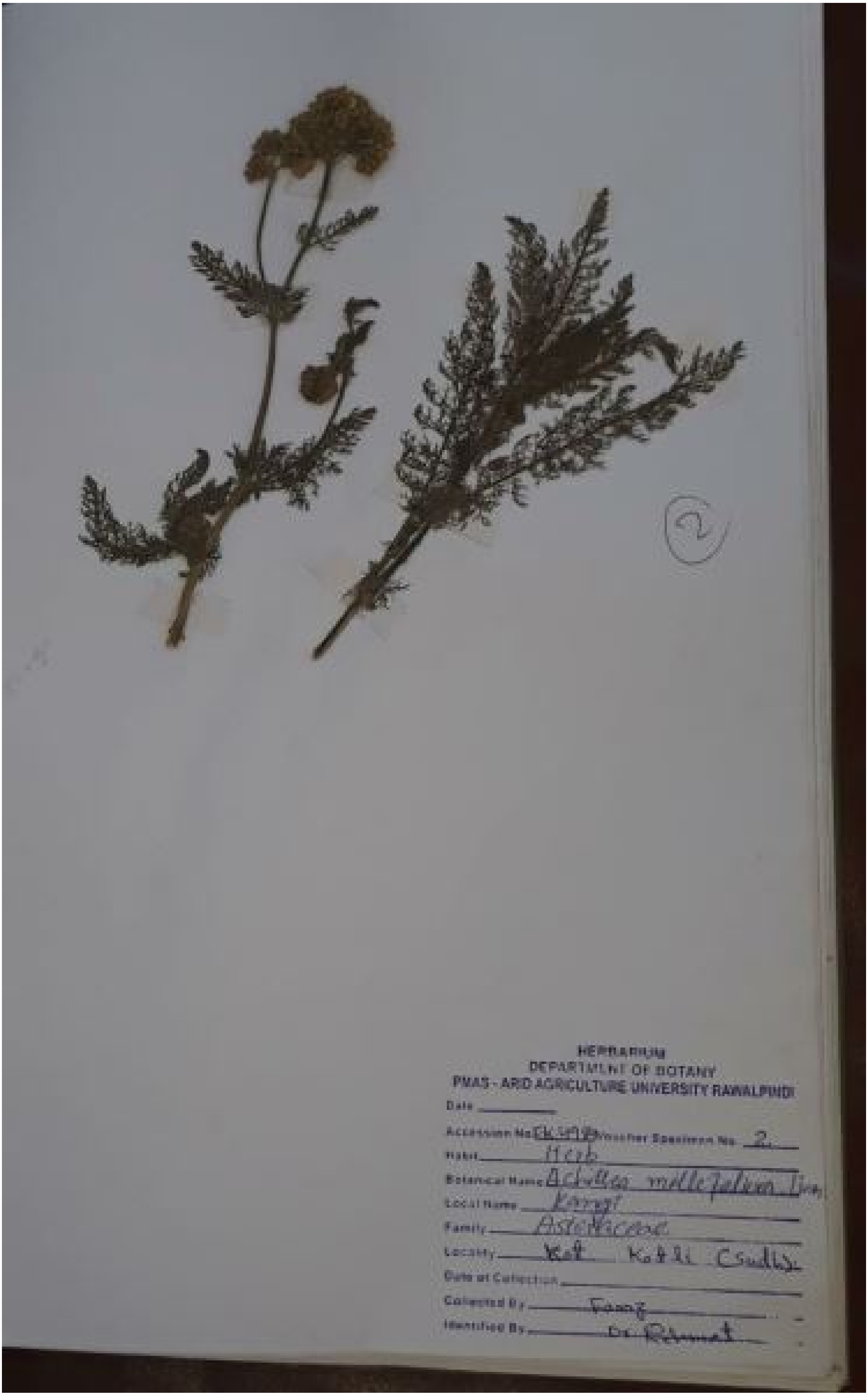
HERBARIUM
DEPARTMENT OF BOTANY
PMAS - ARID AGRICULTURE UNIVERSITY RAWALPINDI
Date
Accession No. [illegible] Voucher Specimen No. 2
Habit Herb
Botanical Name Achillea millefolium Linn.
Local Name Kangi
Family Asteraceae
Locality Kot Kotli [illegible]
Date of Collection
Collected By [illegible]
Identified By Dr. Rehmat

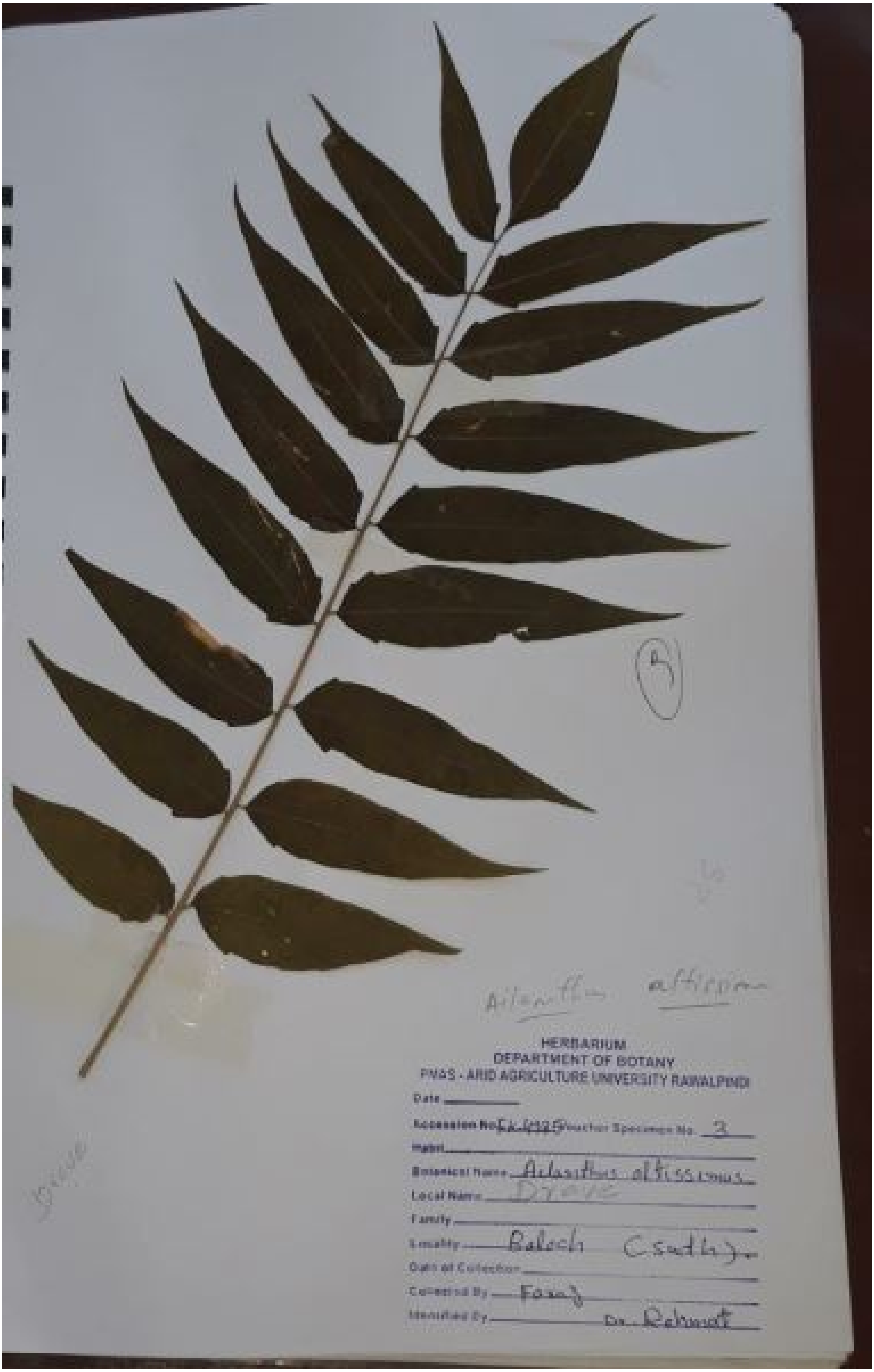
HERBARIUM
DEPARTMENT OF BOTANY
PMAS - ARID AGRICULTURE UNIVERSITY RAWALPINDI
Date
Voucher Specimen No. 3
Botanical Name
Local Name
Family
Locality
Date of Collection
Collected By
Identified By

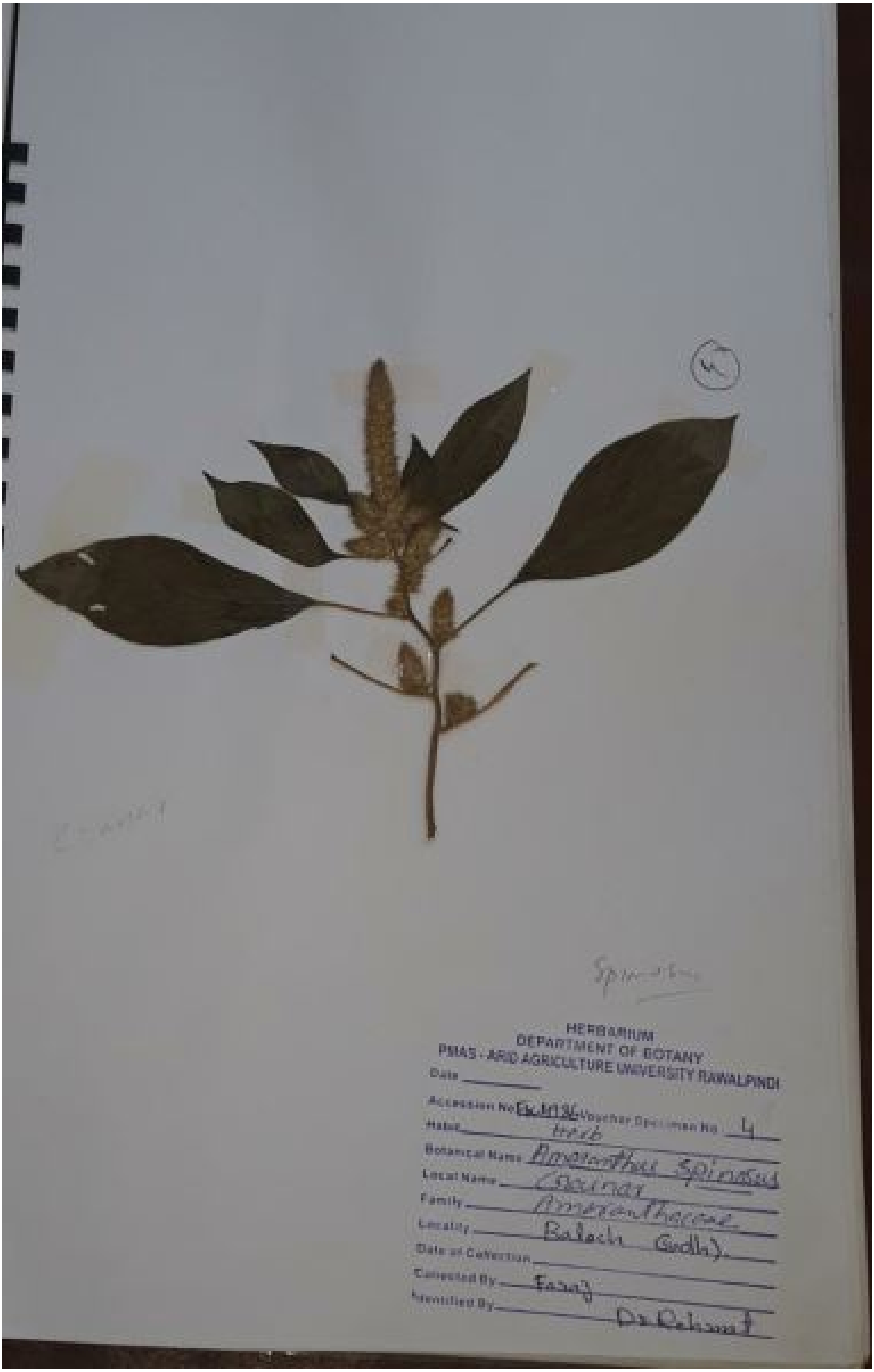
HERBARIUM
DEPARTMENT OF BOTANY
PMAS - ARID AGRICULTURE UNIVERSITY RAWALPINDI
Date
Accession No. [illegible] Voucher Specimen No. 4
Habit Herb
Botanical Name Amaranthus spinosus
Local Name Cholai
Family Amaranthaceae
Locality Baloch (Gadh)
Date of Collection
Collected By Fazal
Identified By Dr. Rahmat

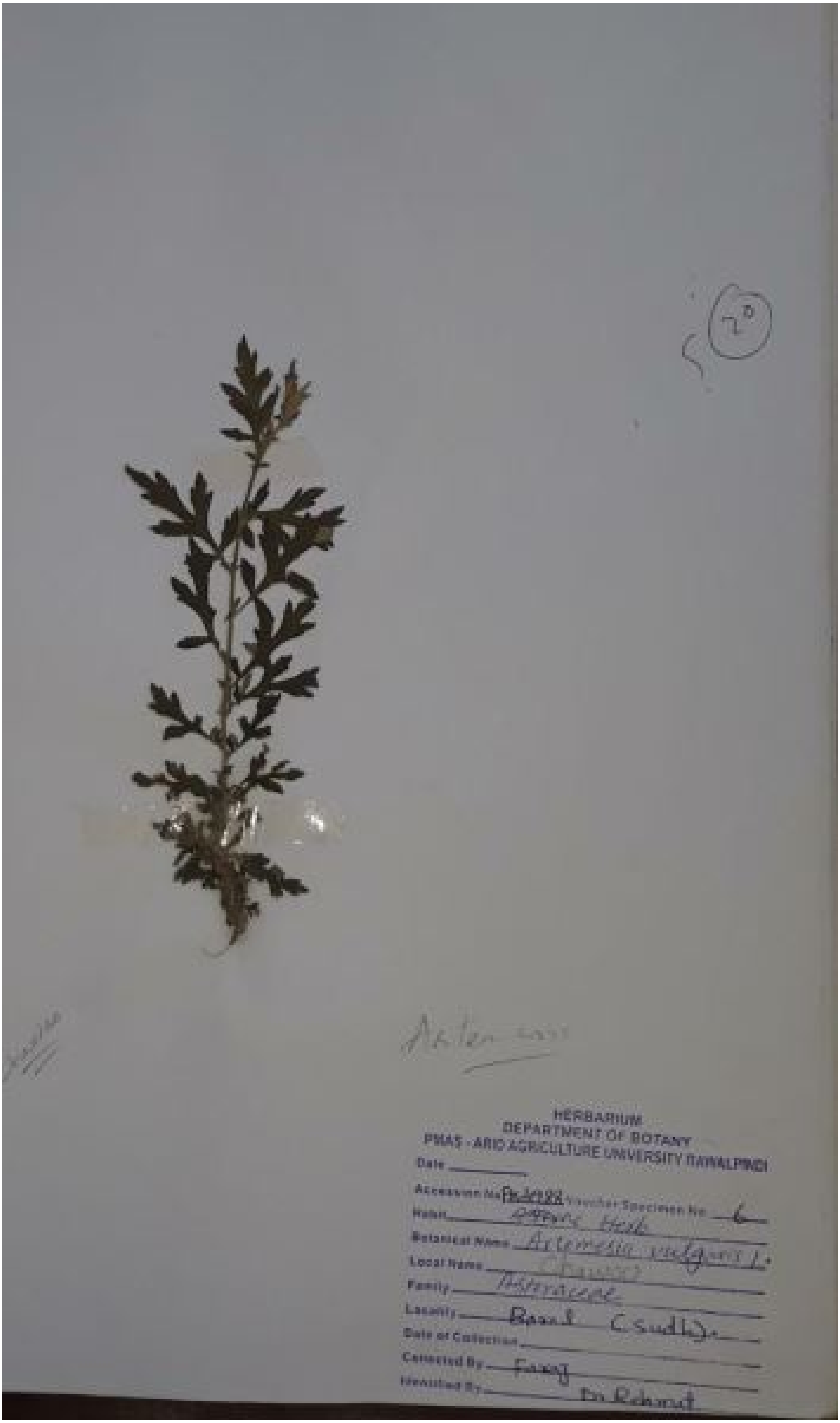
HERBARIUM
DEPARTMENT OF BOTANY
PMAS - ARID AGRICULTURE UNIVERSITY RAWALPINDI
Date
Accession No. Voucher Specimen No. 6
Habit Herb
Botanical Name Artemisia vulgaris L.
Local Name
Family Asteraceae
Locality
Date of Collection
Collected By
Identified By

EMORY UNIVERSITY
022195
HERBARIUM

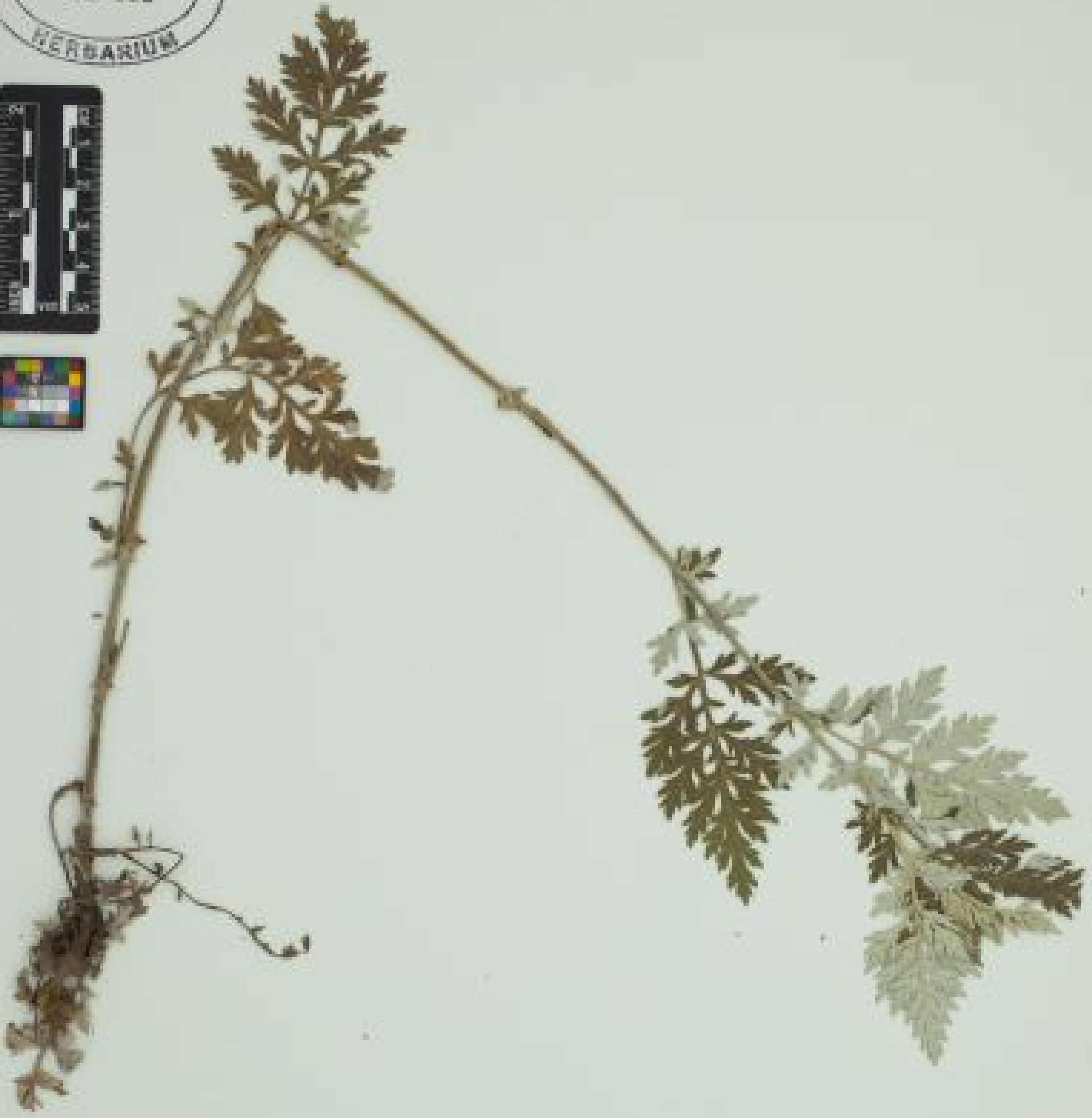

INCORPORATED INTO THE
MEDICAL BOTANY SPECIAL COLLECTION

FK-106

**EMORY UNIVERSITY HERBARIUM**
FLORA OF ISLAMIC REPUBLIC OF PAKISTAN

*Artemisia absinthium* L. ASTERACEAE
"*absinthium*"

Draka, Poonch, Azad Kashmir, Islamic Republic of Pakistan

wild herb in moist habitat

herb, flowers whitish yellow, stem hollow

Whole plant used for digestive disorders and to treat hyperglycaemia
local name: [illegible]

Muhammad Faraz Khan FK-106 5 June 2017

[illegible] 26-September-2017

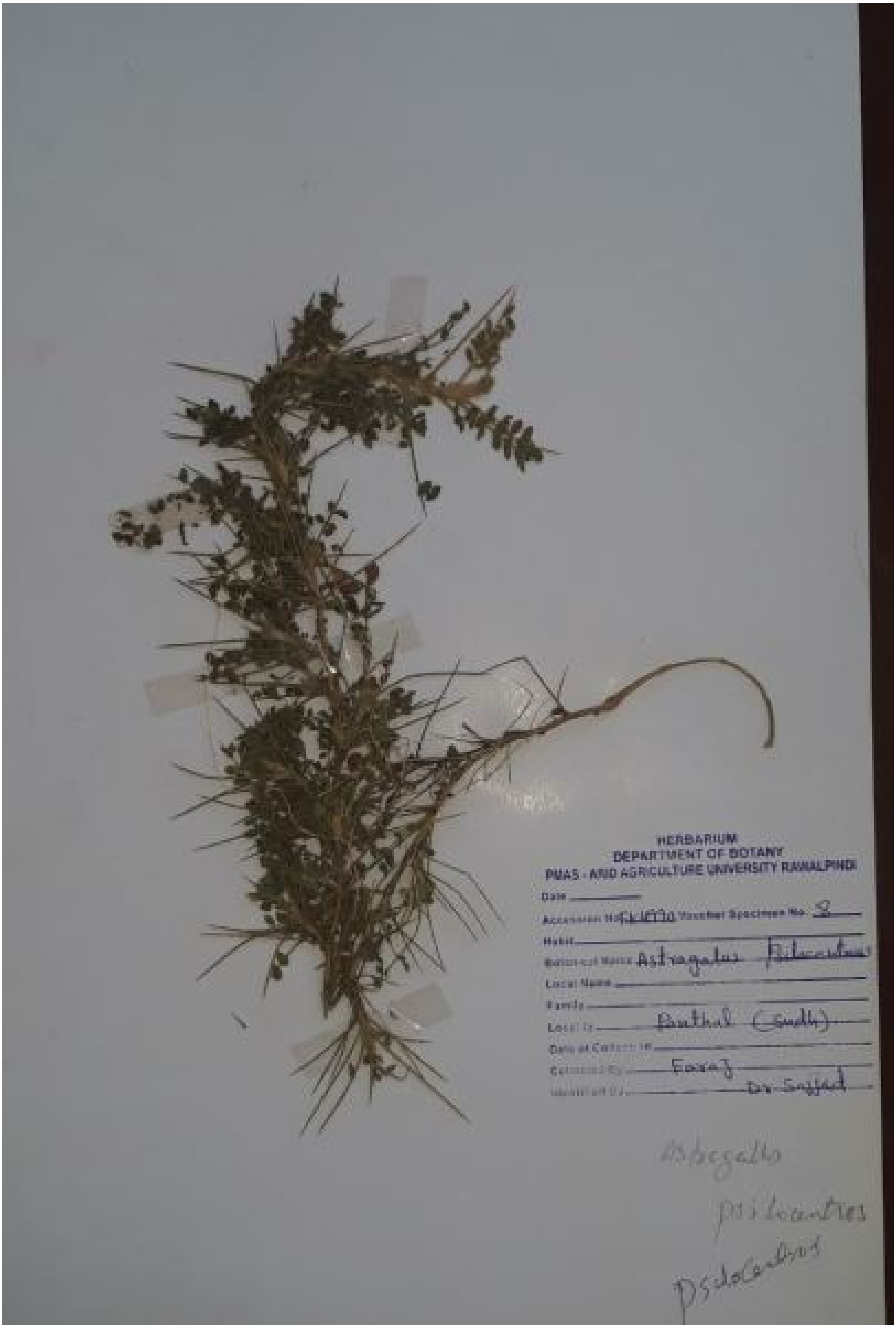
HERBARIUM
DEPARTMENT OF BOTANY
PMAS - ARID AGRICULTURE UNIVERSITY RAWALPINDI
Date
Voucher Specimen No.
Habit
Astragalus
Local Name
Family
Faraz
Dr. Sajjad

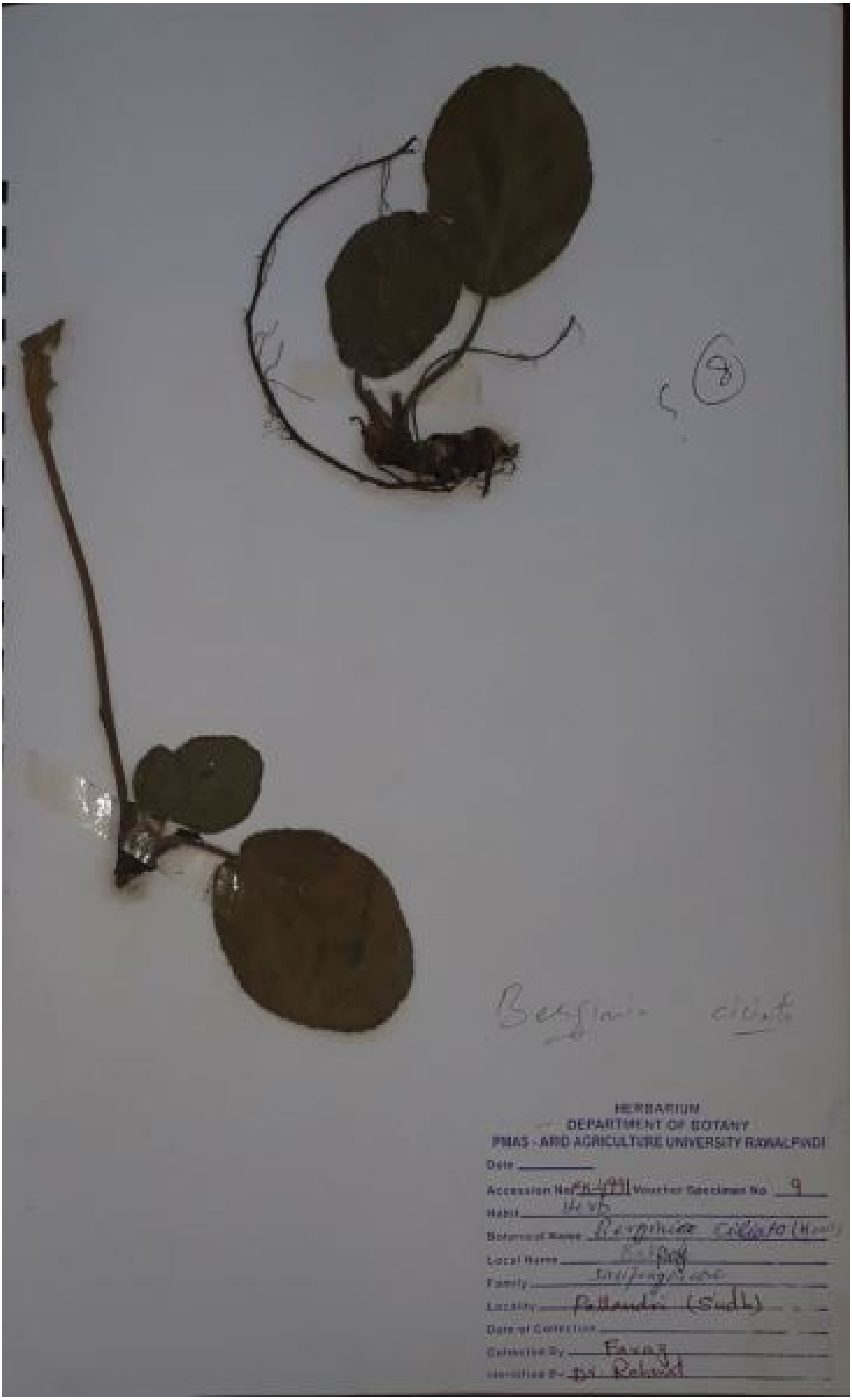
HERBARIUM
DEPARTMENT OF BOTANY
PMAS - ARID AGRICULTURE UNIVERSITY RAWALPINDI
Date
Accession No
Voucher Specimen No 9
Habit
Botanical Name
Local Name
Family
Locality
Date of Collection
Collected By
Identified By

EMORY UNIVERSITY
022196
HERBARIUM

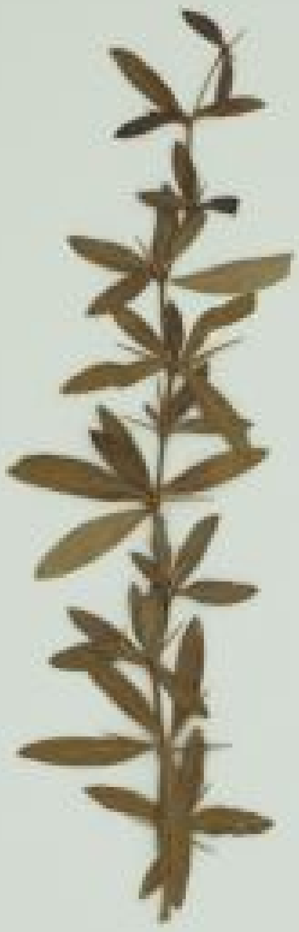

INCORPORATED INTO THE
MEDICAL BOTANY SPECIAL COLLECTION

FK-105

**EMORY UNIVERSITY HERBARIUM**
FLORA OF ISLAMIC REPUBLIC OF PAKISTAN

*Berberis lycium* Royle — BERBERIDACEAE

Gorah, Sudhnoti, Azad Kashmir, Islamic Republic of Pakistan

common in dry habitat

flowers yellow in color stem prickly

root decoction is used to treat diabetes, piles and jaundice, enteric infections local name: Sumbal

Muhammad Faraz Khan FK-105 — 5 June 2017

Michelle Lin — 21-September-2017

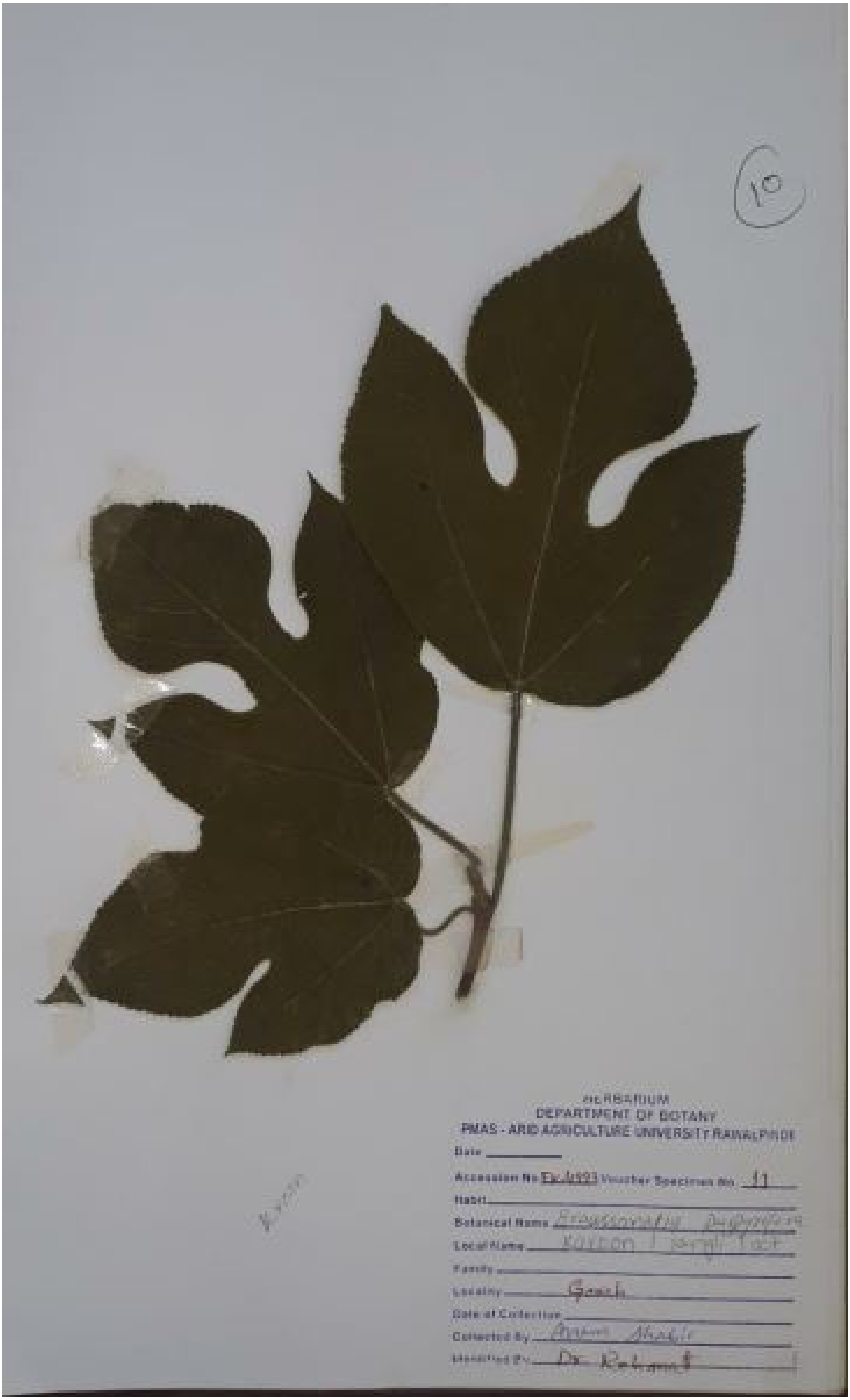
10
HERBARIUM
DEPARTMENT OF BOTANY
PMAS - ARID AGRICULTURE UNIVERSITY RAWALPINDI
Date
Accession No
Voucher Specimen No. 11
Habit
Botanical Name
Local Name
Family
Locality Gosh
Date of Collection
Collected By
Identified By Dr. Rahmat

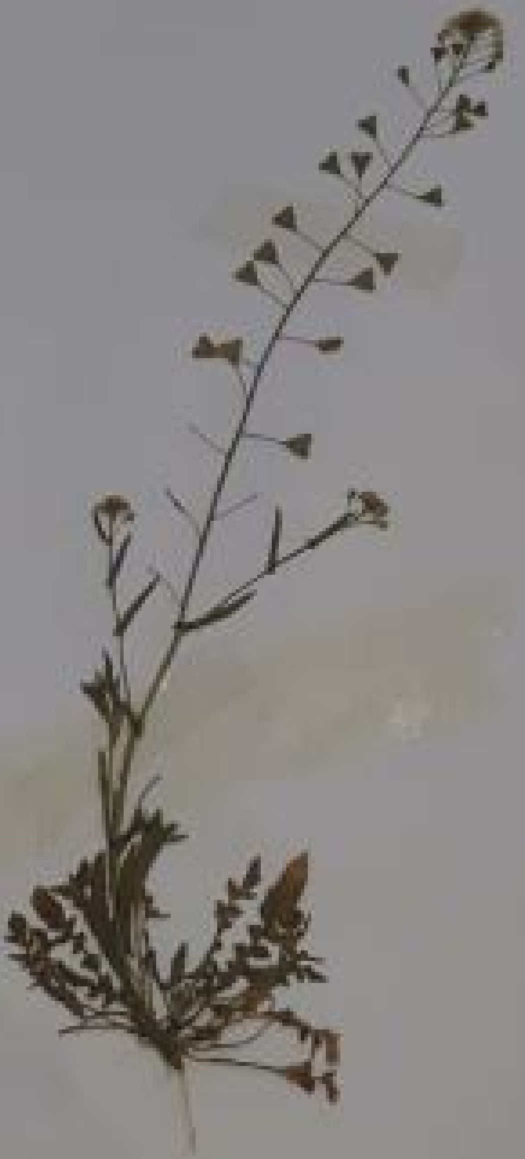

32

**HERBARIUM**
**DEPARTMENT OF BOTANY**
**PMAS - ARID AGRICULTURE UNIVERSITY RAWALPINDI**

Date ________

Accession No. [illegible] Voucher Specimen No. 12

Habit ________

Botanical Name capsella - bursa - pastoris

Local Name ________

Family Brassicaceae

Locality Pallandri (Sudh)

Date of Collection ________

Collected By Faraz

Identified By Dr. Rehmat

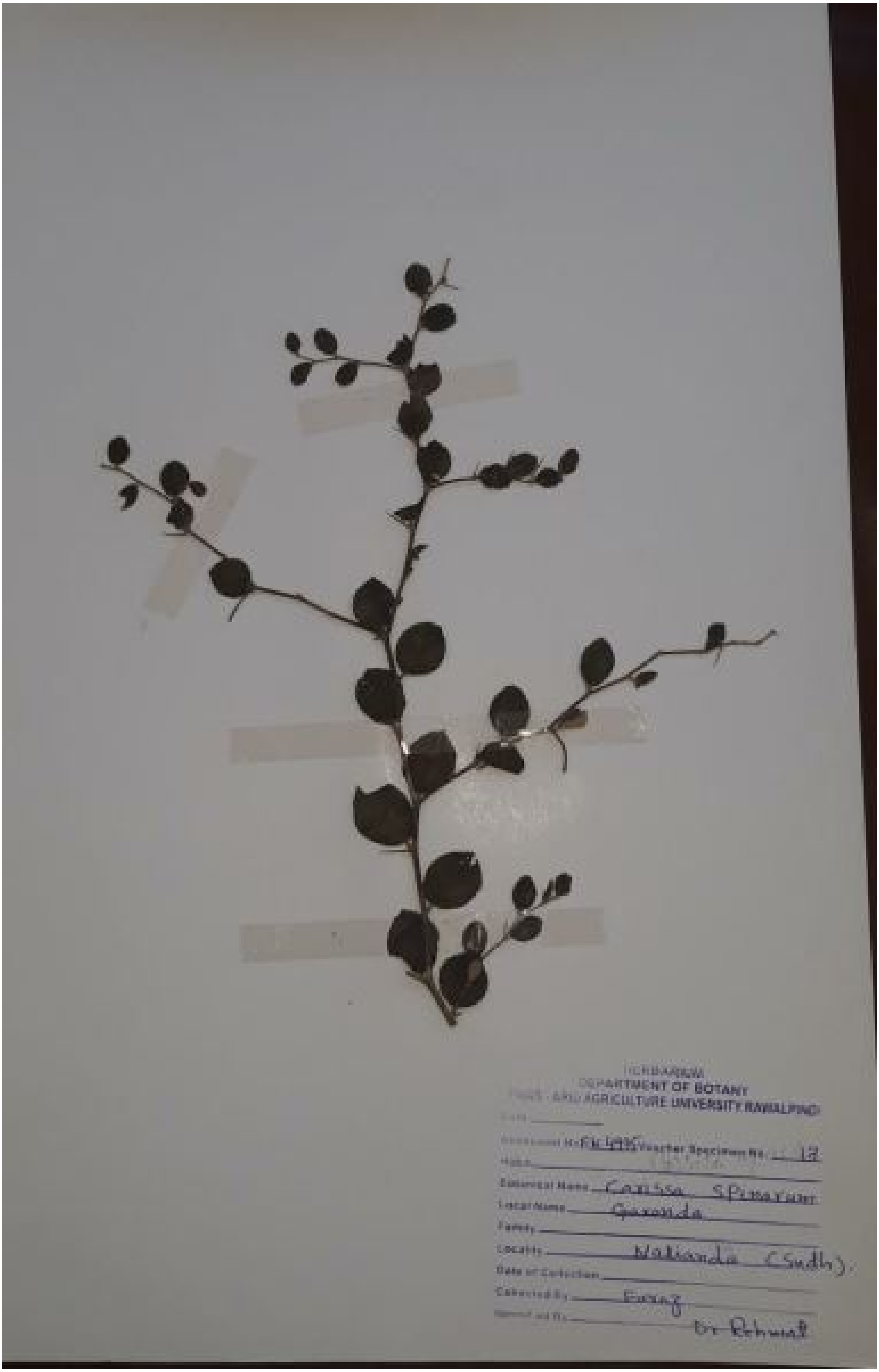
DEPARTMENT OF BOTANY
AGRICULTURE UNIVERSITY RAWALPINDI
Voucher Specimen No. 12
Carissa spinarum
Local Name Garanda
Family
Date of Collection
Dr Rehmat

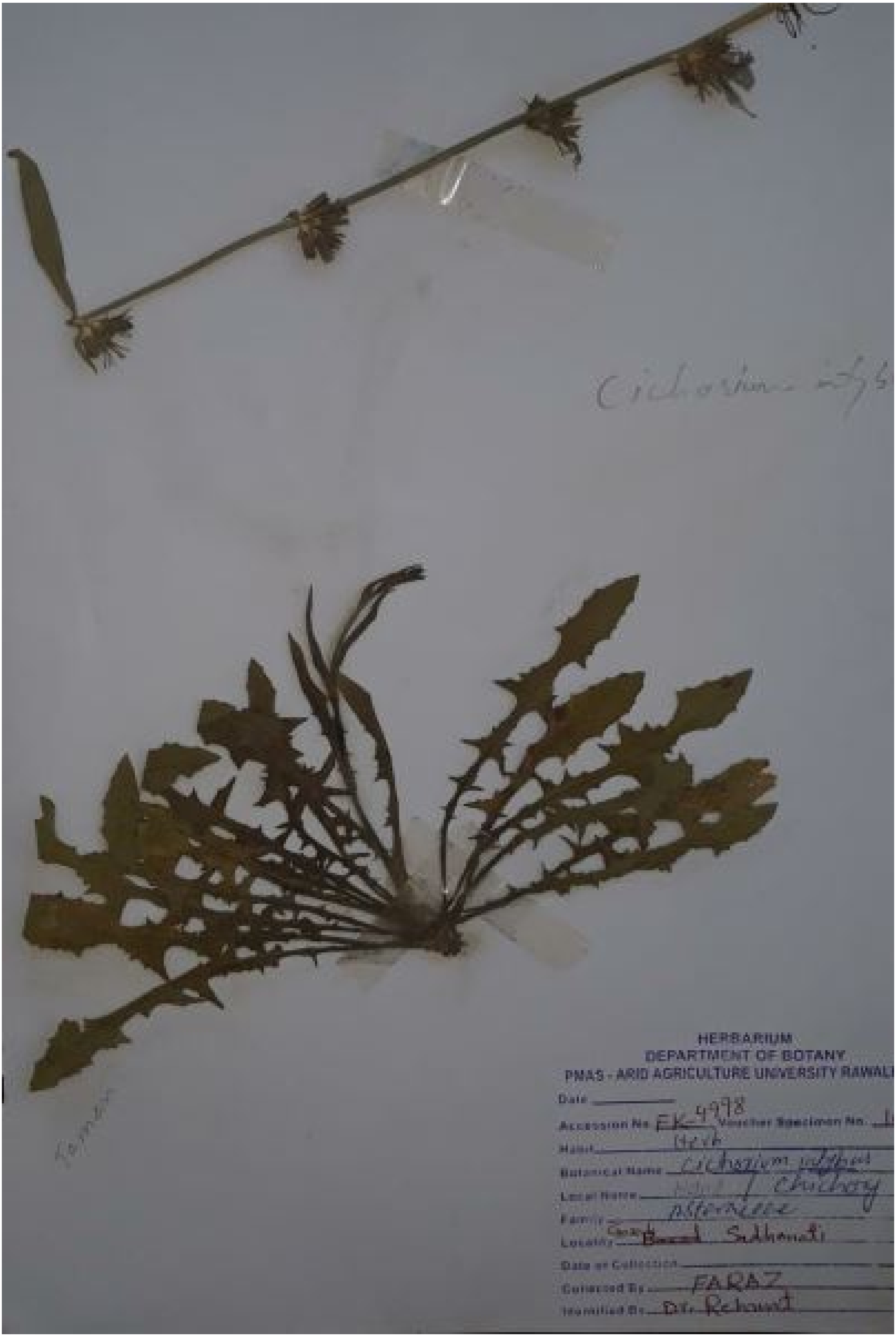
HERBARIUM
DEPARTMENT OF BOTANY
PMAS - ARID AGRICULTURE UNIVERSITY RAWAL
Date
Accession No. EK-4998 Voucher Specimen No.
Habit Herb
Botanical Name
Local Name
Family
Locality
Date of Collection
Collected By FARAZ
Identified By Dr.

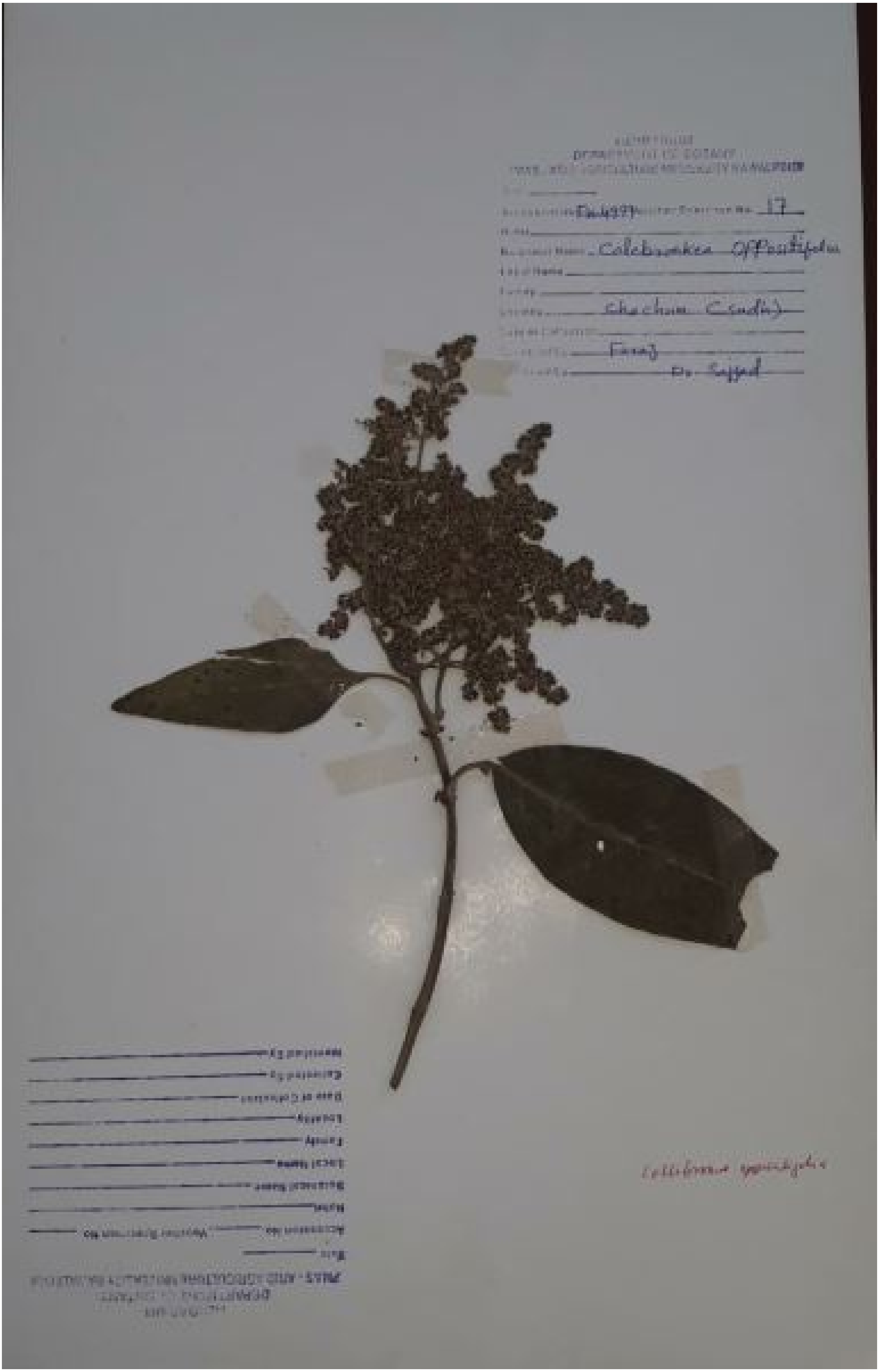
17
Colebrookea Oppositifolia

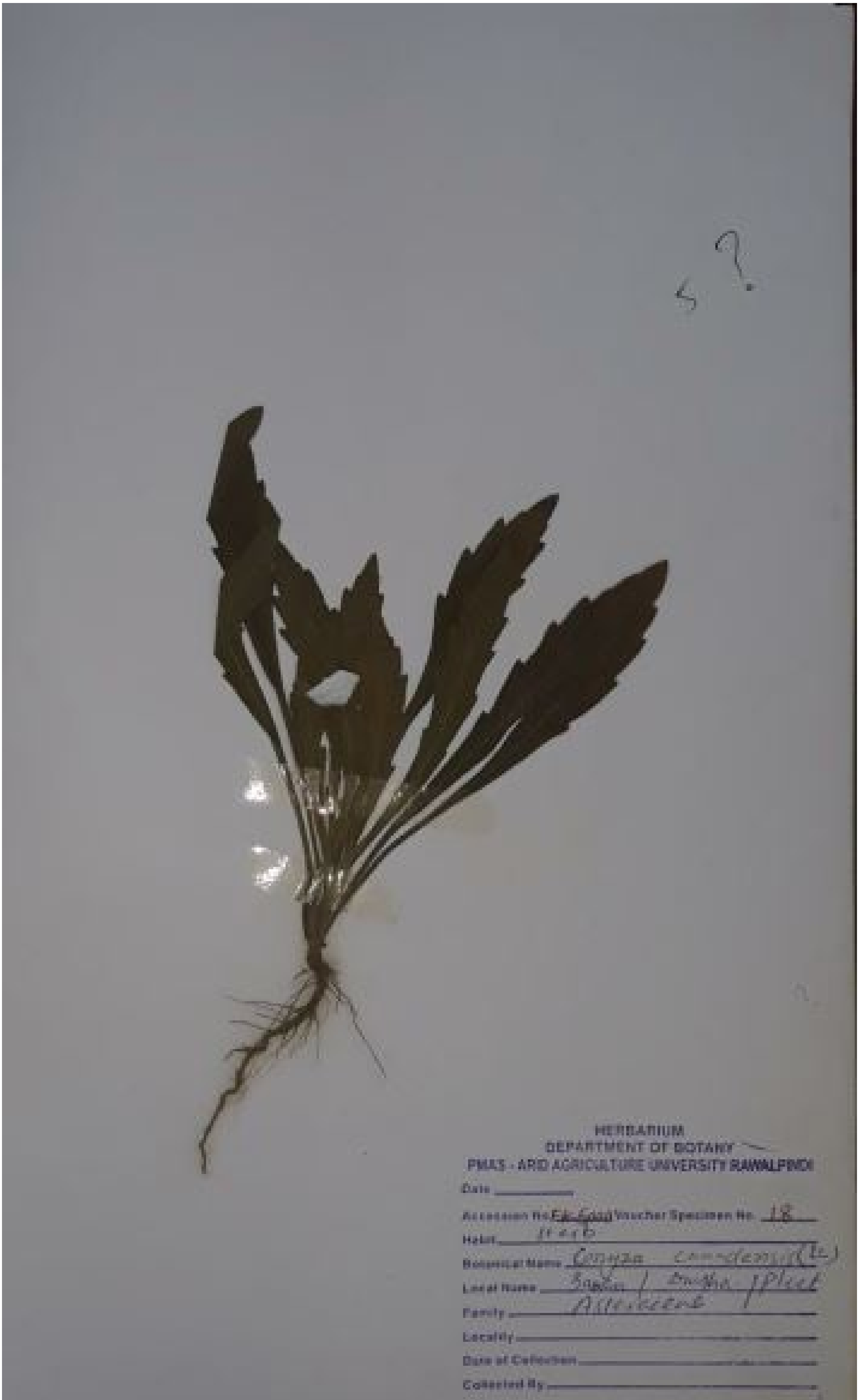
HERBARIUM
DEPARTMENT OF BOTANY
PMAS - ARID AGRICULTURE UNIVERSITY RAWALPINDI
Date
Accession No./Voucher Specimen No. 18
Habit
Botanical Name Conyza canadensis (L.)
Local Name
Family Asteraceae
Locality
Date of Collection
Collected By
Identified By

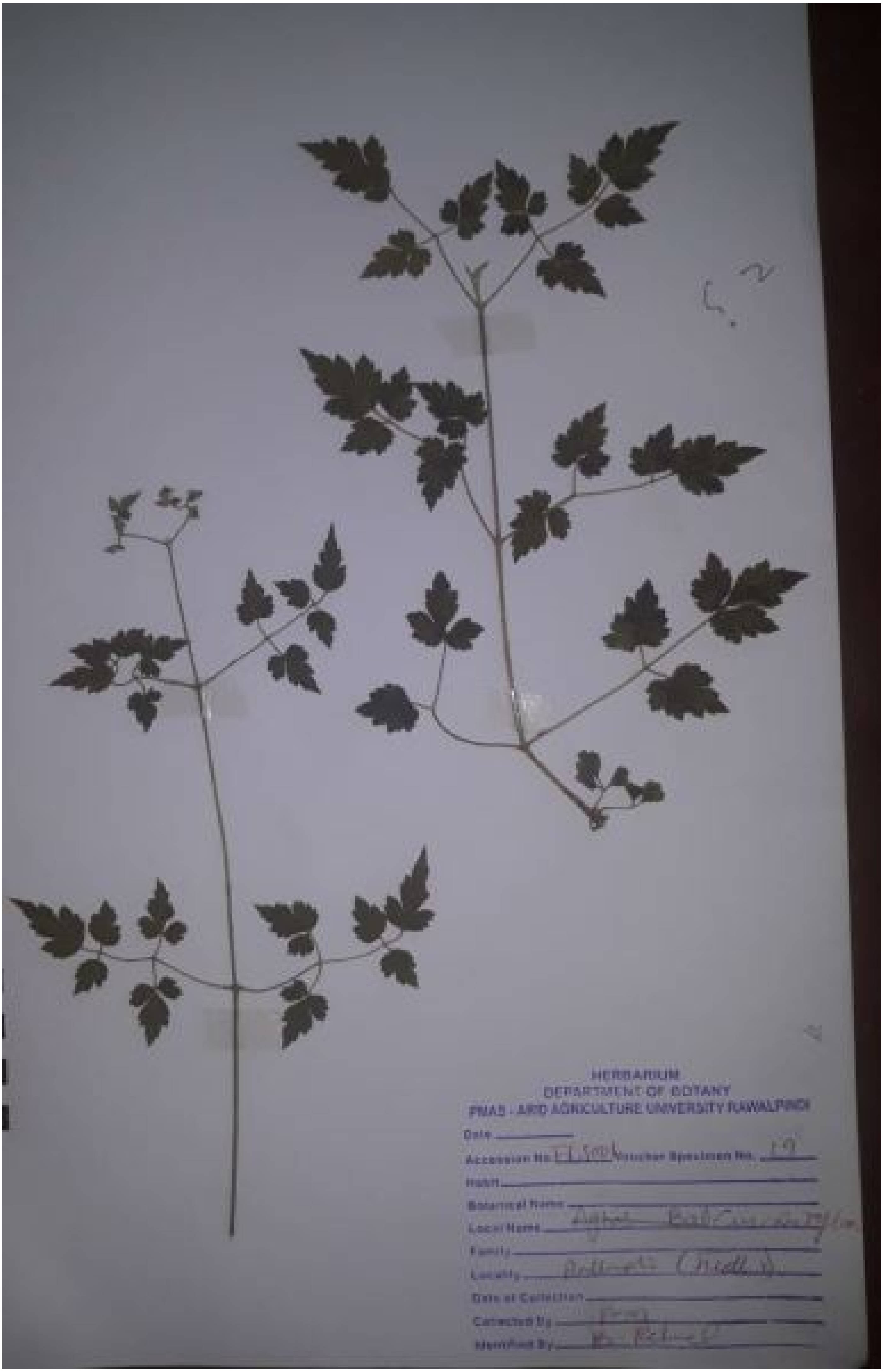
HERBARIUM
DEPARTMENT OF BOTANY
PMAS - ARID AGRICULTURE UNIVERSITY RAWALPINDI
Date
Accession No
Voucher Specimen No.
Habit
Botanical Name
Local Name
Family
Locality
Date of Collection
Collected By
Identified By

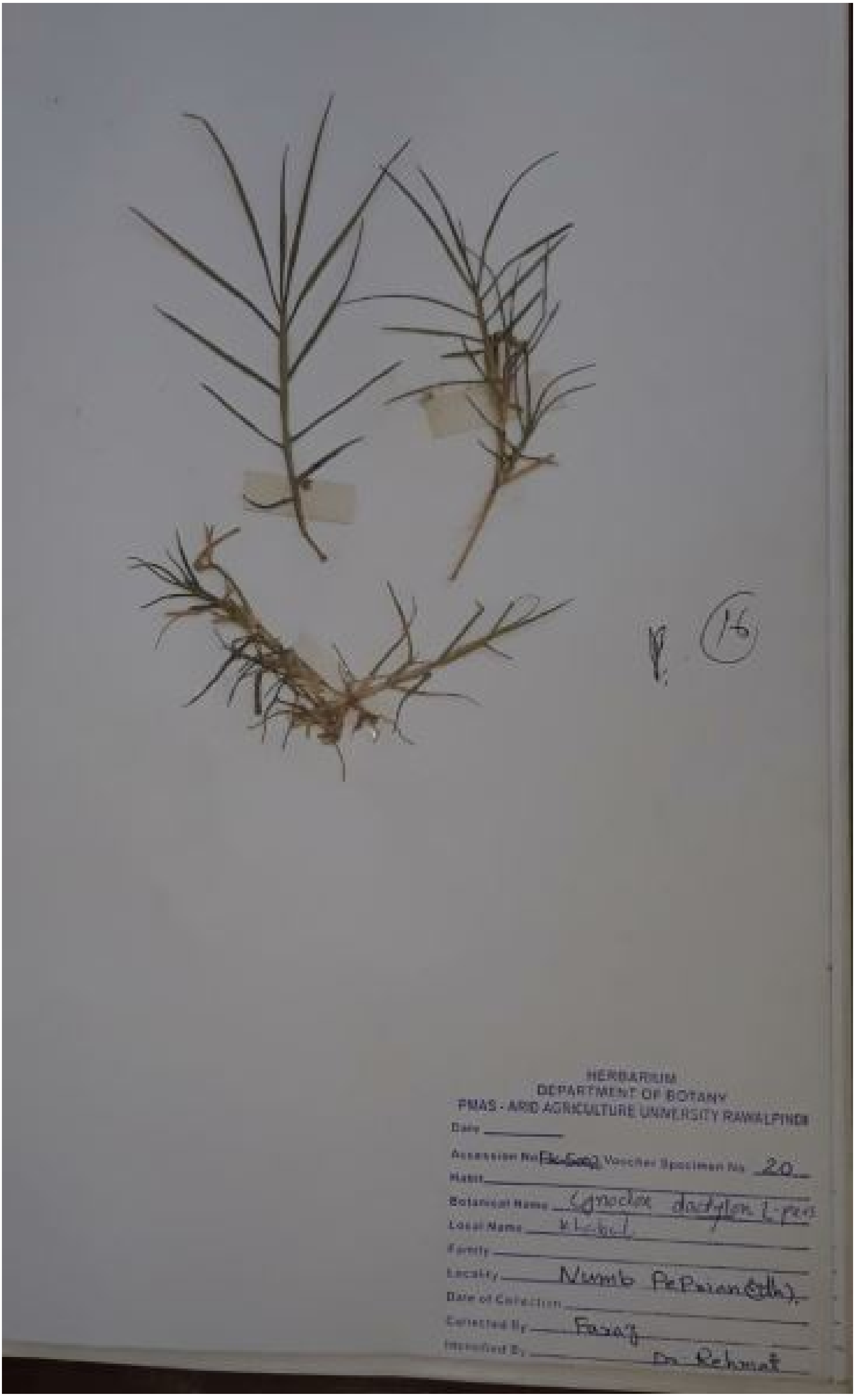
HERBARIUM
DEPARTMENT OF BOTANY
PMAS - ARID AGRICULTURE UNIVERSITY RAWALPINDI
Date
Voucher Specimen No. 20
Habit
Botanical Name Cynodon dactylon L. pers
Family
Locality Numb Pepran
Date of Collection
Collected By Faraz
Identified By Dr. Rehmat

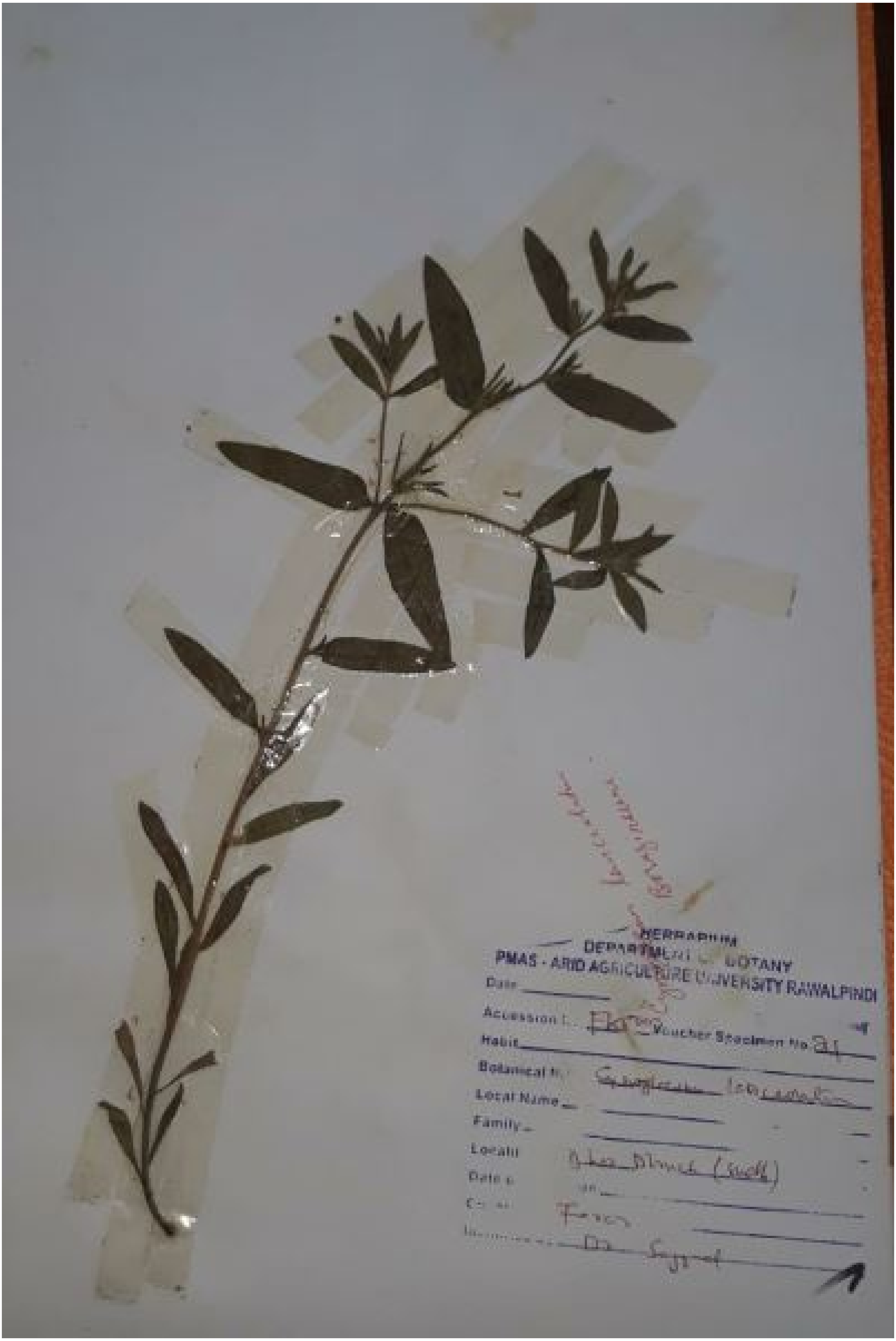
PMAS - ARID AGRICULTURE UNIVERSITY RAWALPINDI
Voucher Specimen No
Habit
Local Name
Family

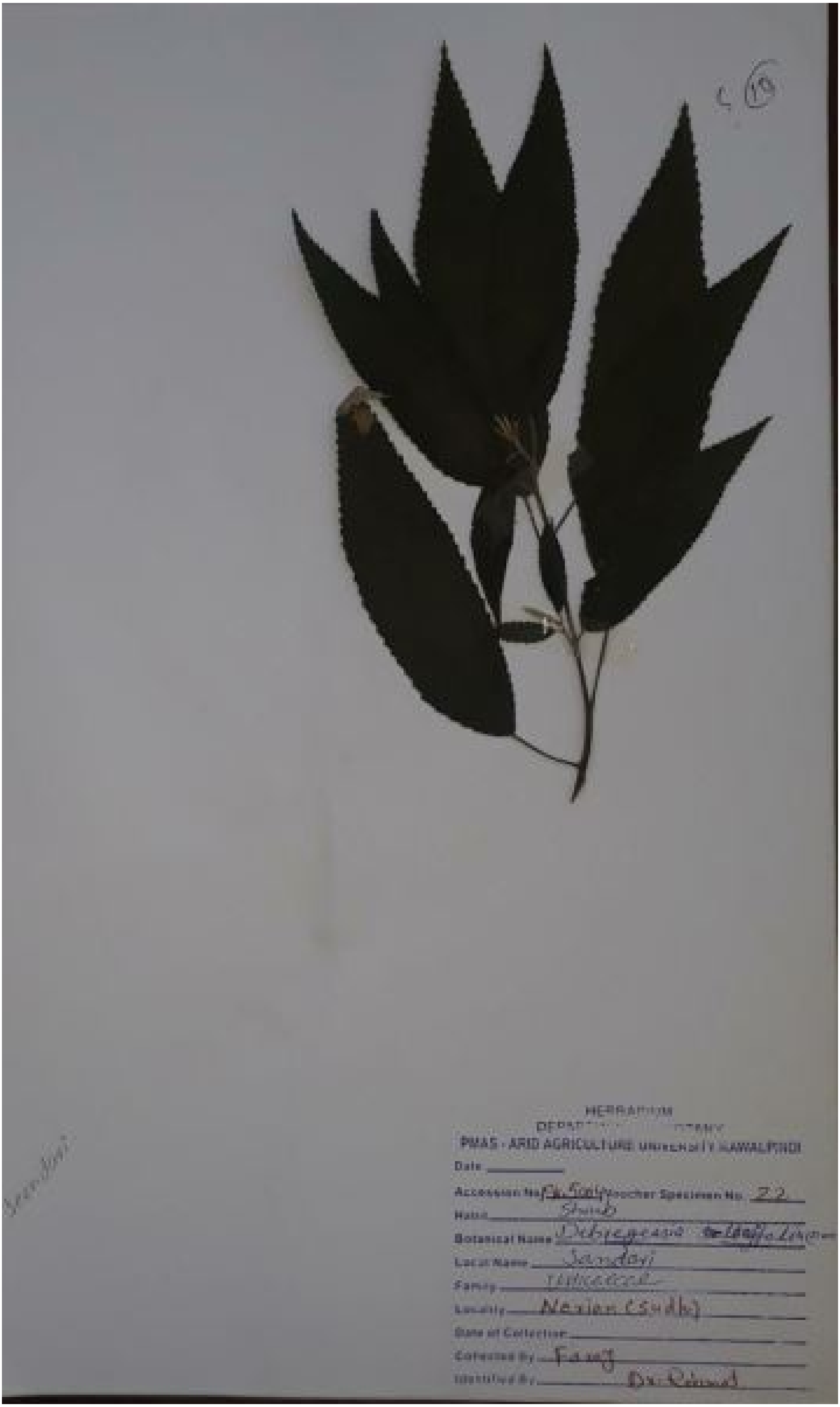

HERBARIUM
PMAS - ARID AGRICULTURE UNIVERSITY RAWALPINDI
Date
Accession No. Voucher Specimen No. 22
Habit Shrub
Botanical Name
Local Name Sandori
Family
Locality Nezian (Sudh)
Date of Collection
Collected By Faraz
Identified By

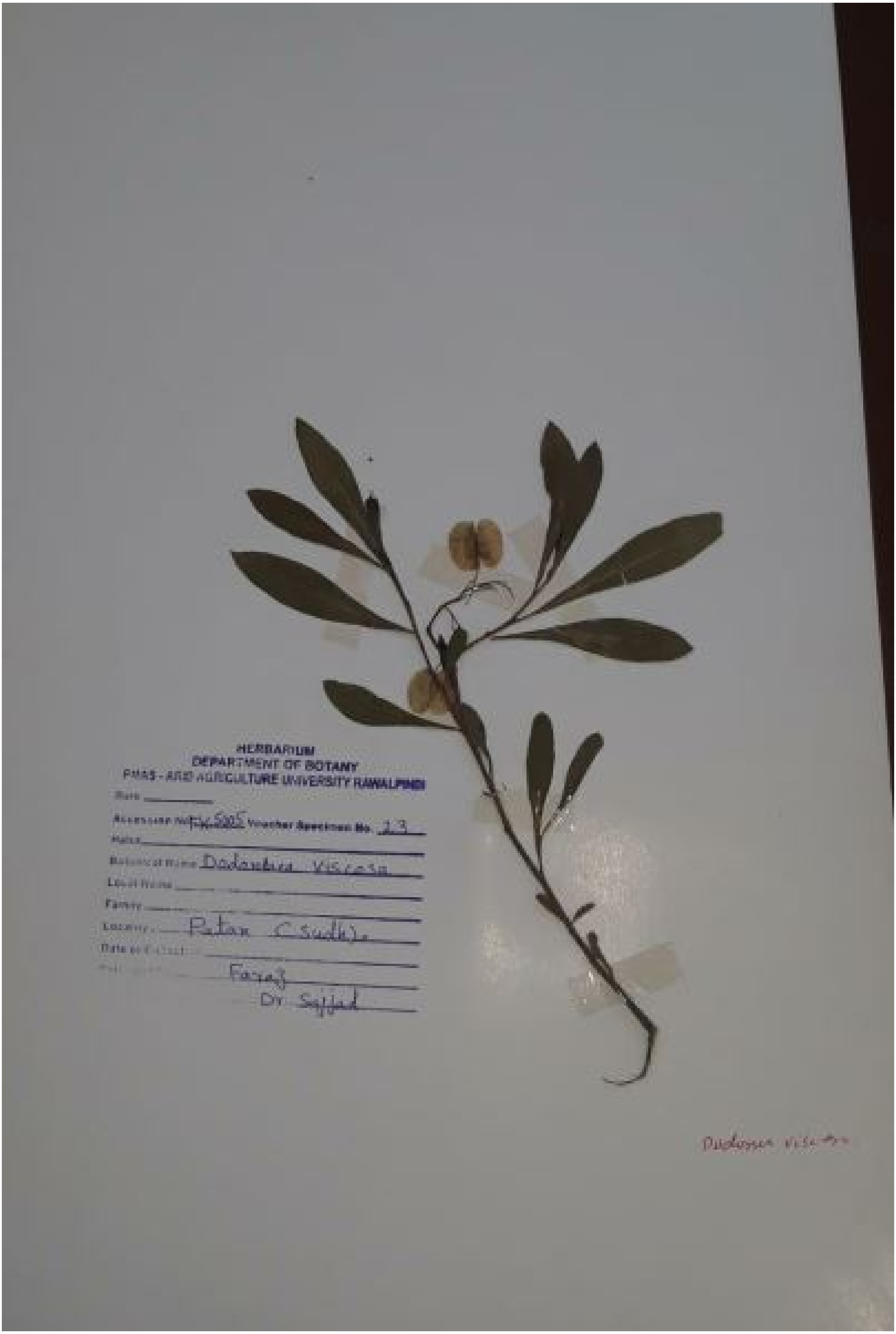
HERBARIUM
DEPARTMENT OF BOTANY
PMAS - ARID AGRICULTURE UNIVERSITY RAWALPINDI
Voucher Specimen No. 23
Dodonaea viscosa
Faraz
Dr. Sajjad

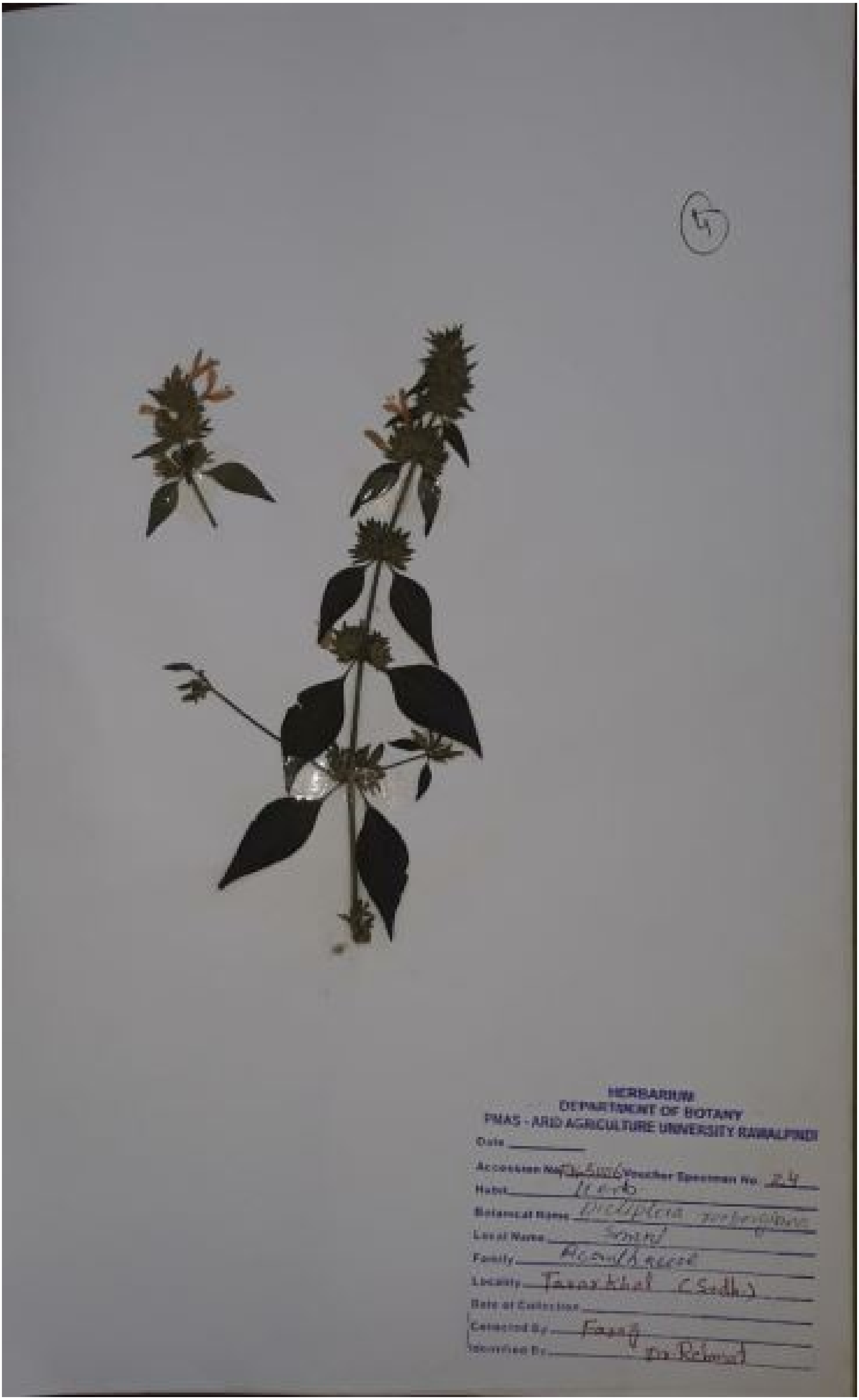
HERBARIUM
DEPARTMENT OF BOTANY
PMAS - ARID AGRICULTURE UNIVERSITY RAWALPINDI
Date
Accession No.
Voucher Specimen No. 24
Habit Herb
Botanical Name Dicliptera
Local Name
Family Acanthaceae
Locality
Date of Collection
Collected By
Identified By

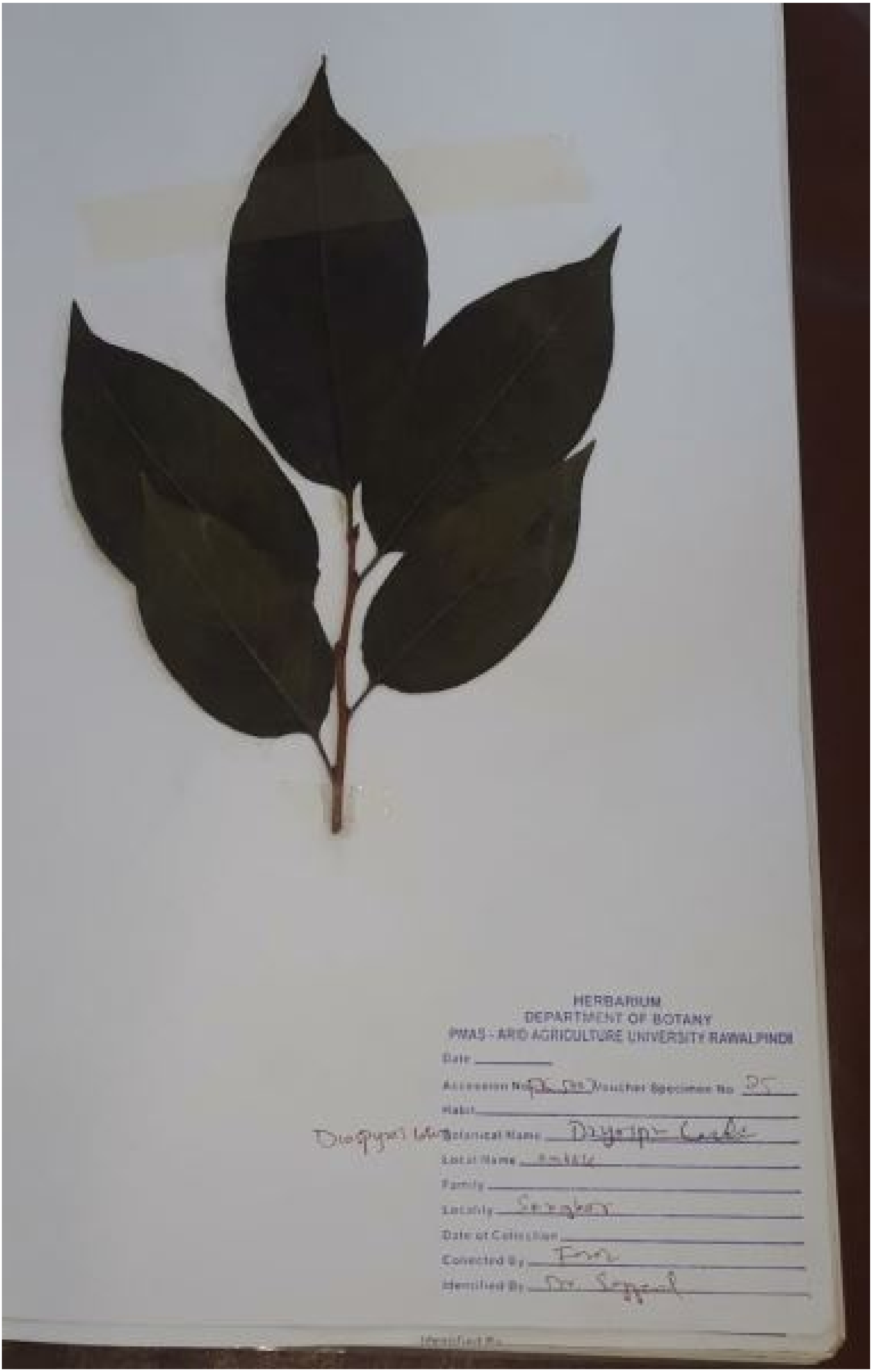
HERBARIUM
DEPARTMENT OF BOTANY
PMAS - ARID AGRICULTURE UNIVERSITY RAWALPINDI
Date
Accession No
Voucher Specimen No
Habit
Botanical Name
Local Name
Family
Locality
Date of Collection
Collected By
Identified By

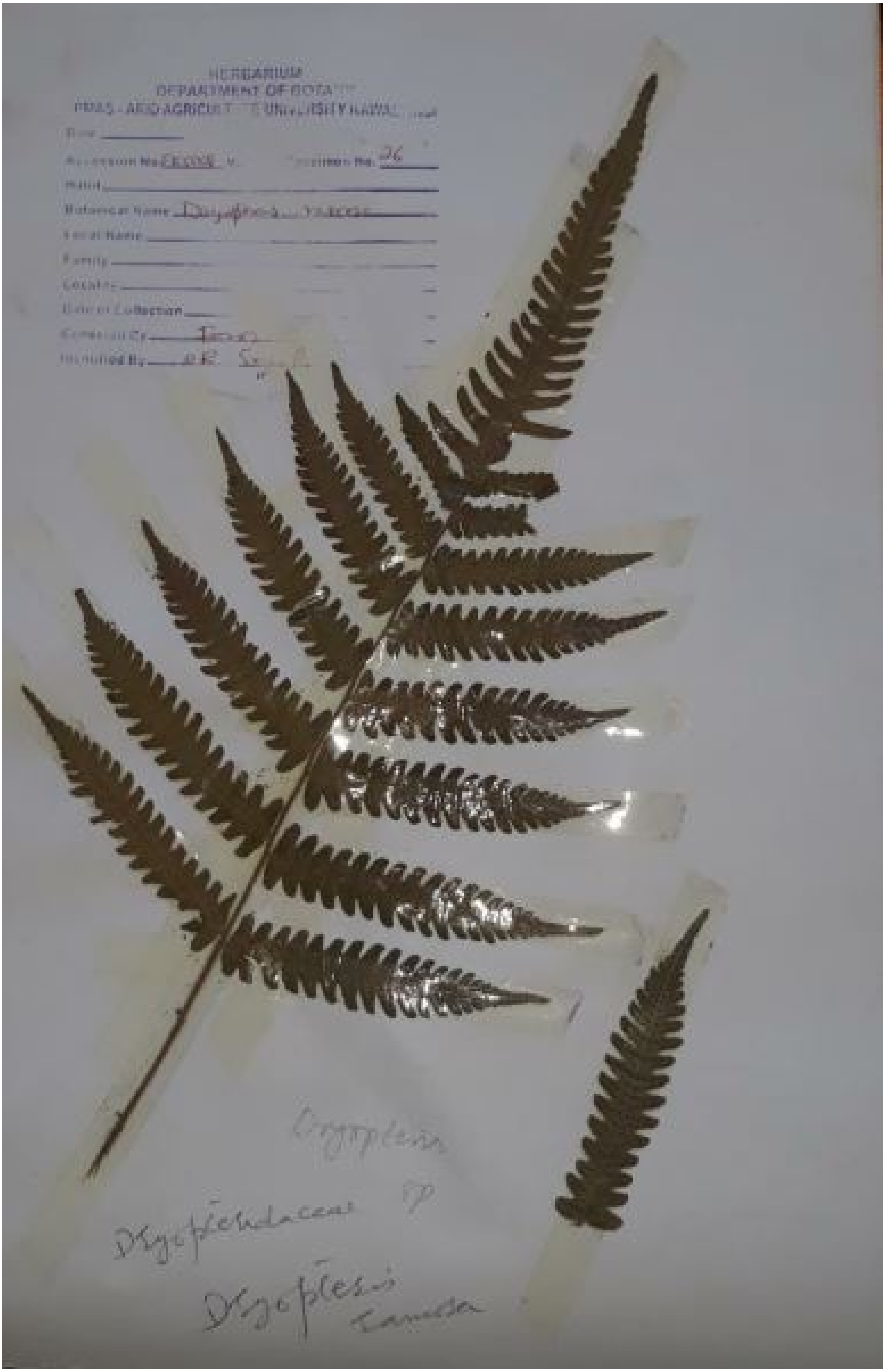
HERBARIUM
Botanical Name
Local Name
Family
Date of Collection
Dryopteridaceae

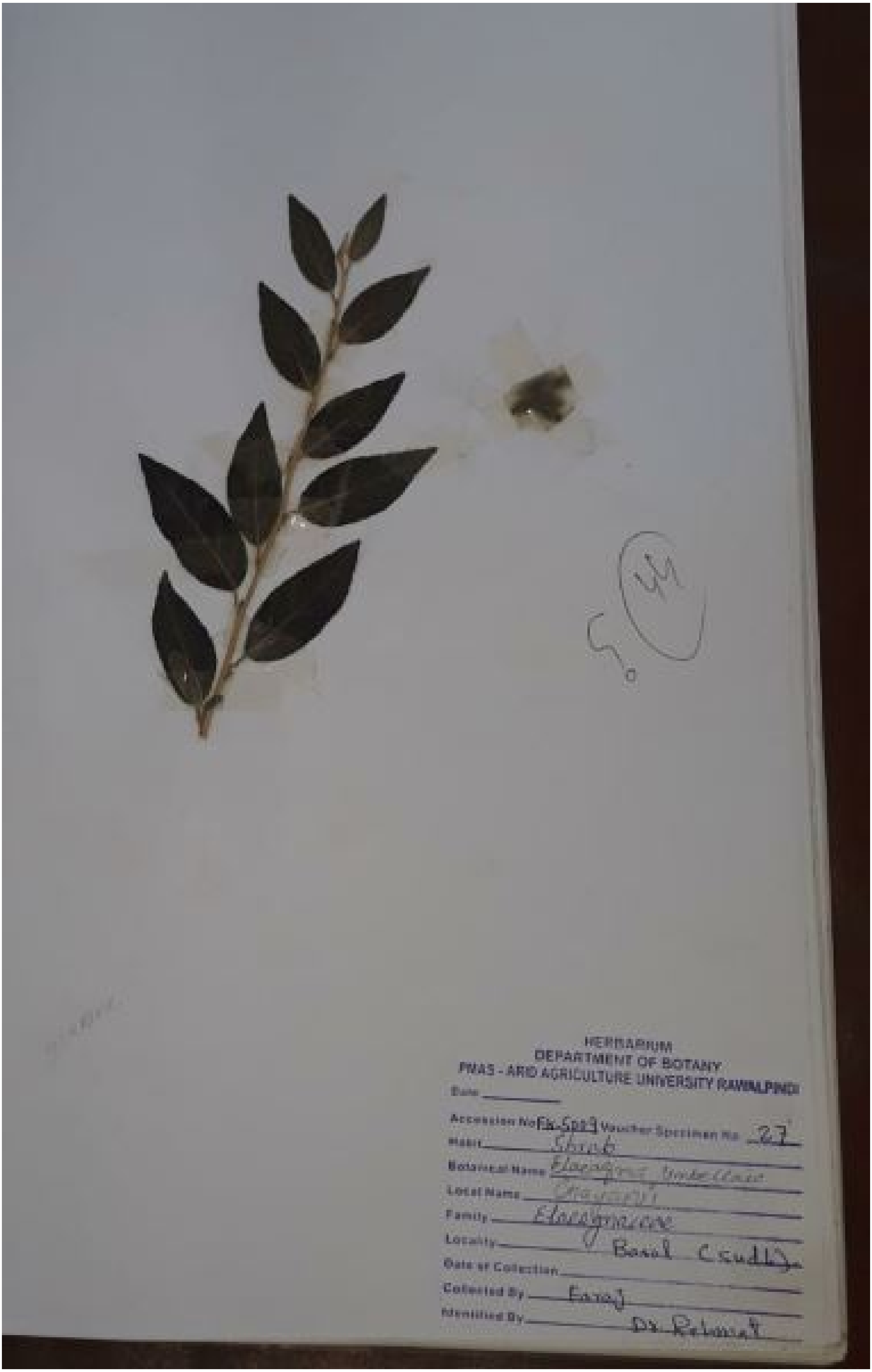
HERBARIUM
DEPARTMENT OF BOTANY
PMAS - ARID AGRICULTURE UNIVERSITY RAWALPINDI
Date
Accession No
Voucher Specimen No. 27
Habit Shrub
Botanical Name
Local Name
Family Elaeagnaceae
Locality
Date of Collection
Collected By Faraj
Identified By Dr. Rahmat

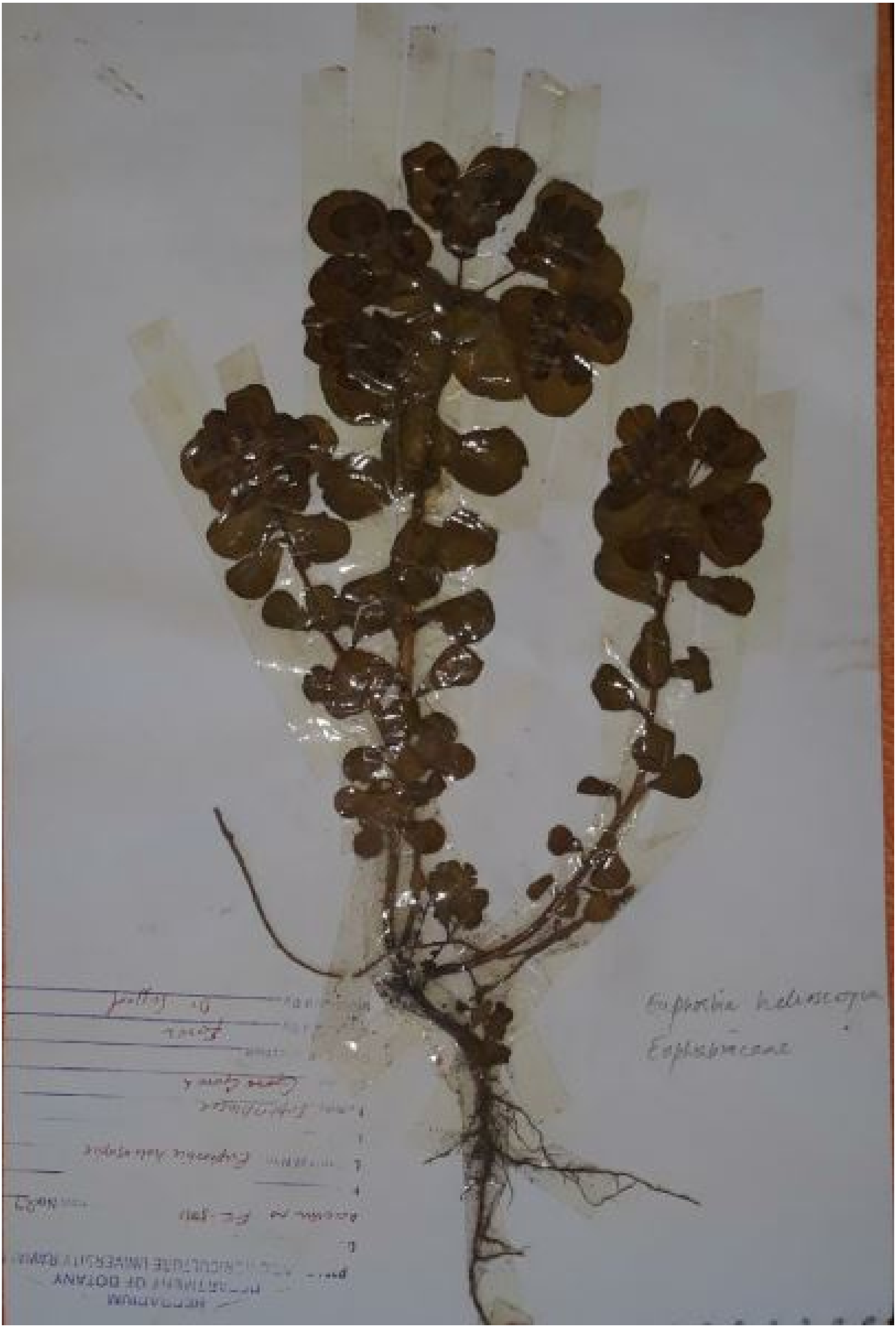
Euphorbia helioscopia
Euphorbiaceae

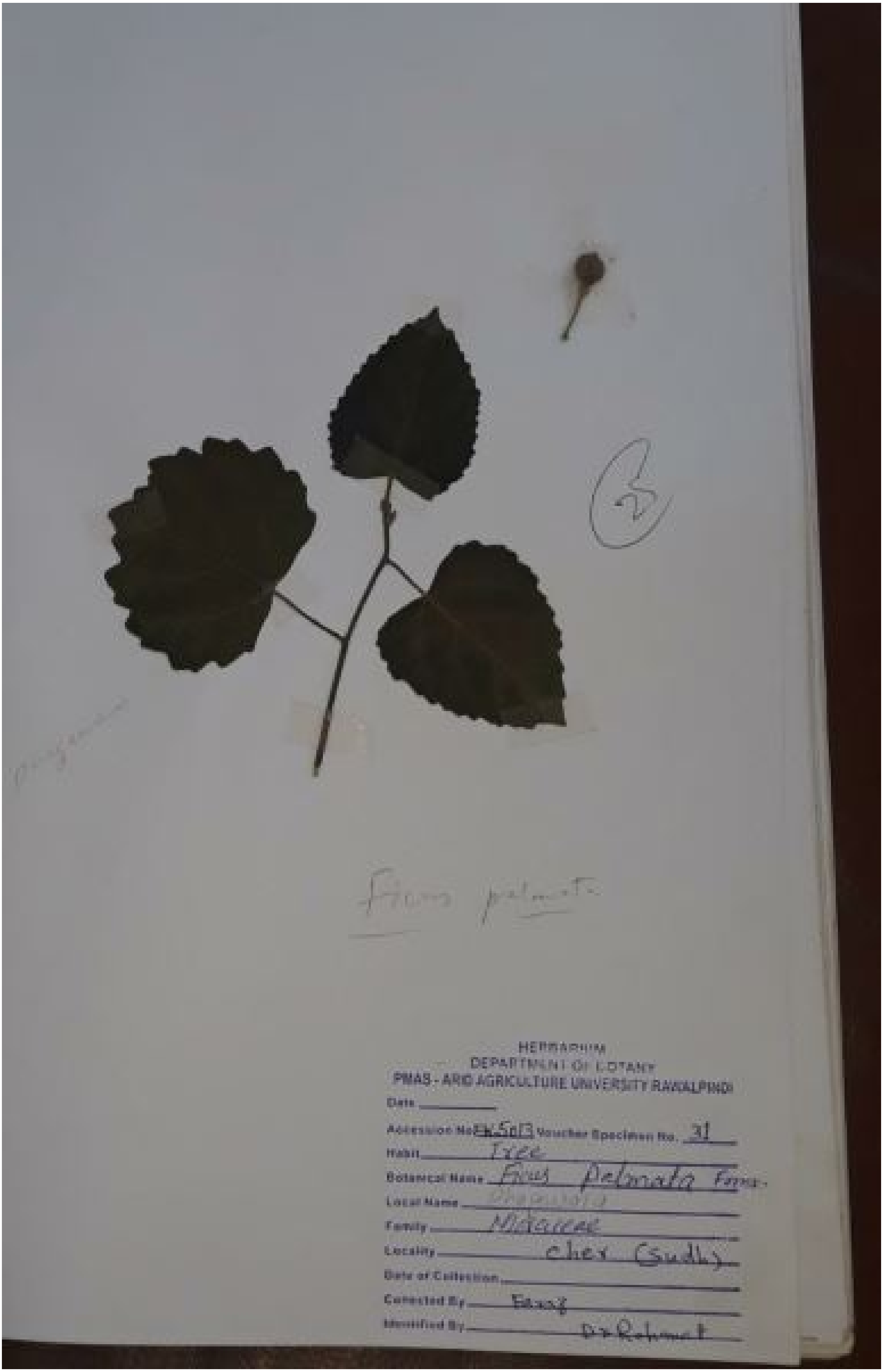
HERBARIUM
DEPARTMENT OF BOTANY
PMAS - ARID AGRICULTURE UNIVERSITY RAWALPINDI
Date
Accession No.
Voucher Specimen No. 31
Habit Tree
Botanical Name Ficus Palmata Forss.
Local Name
Family Moraceae
Locality
Date of Collection
Collected By
Identified By

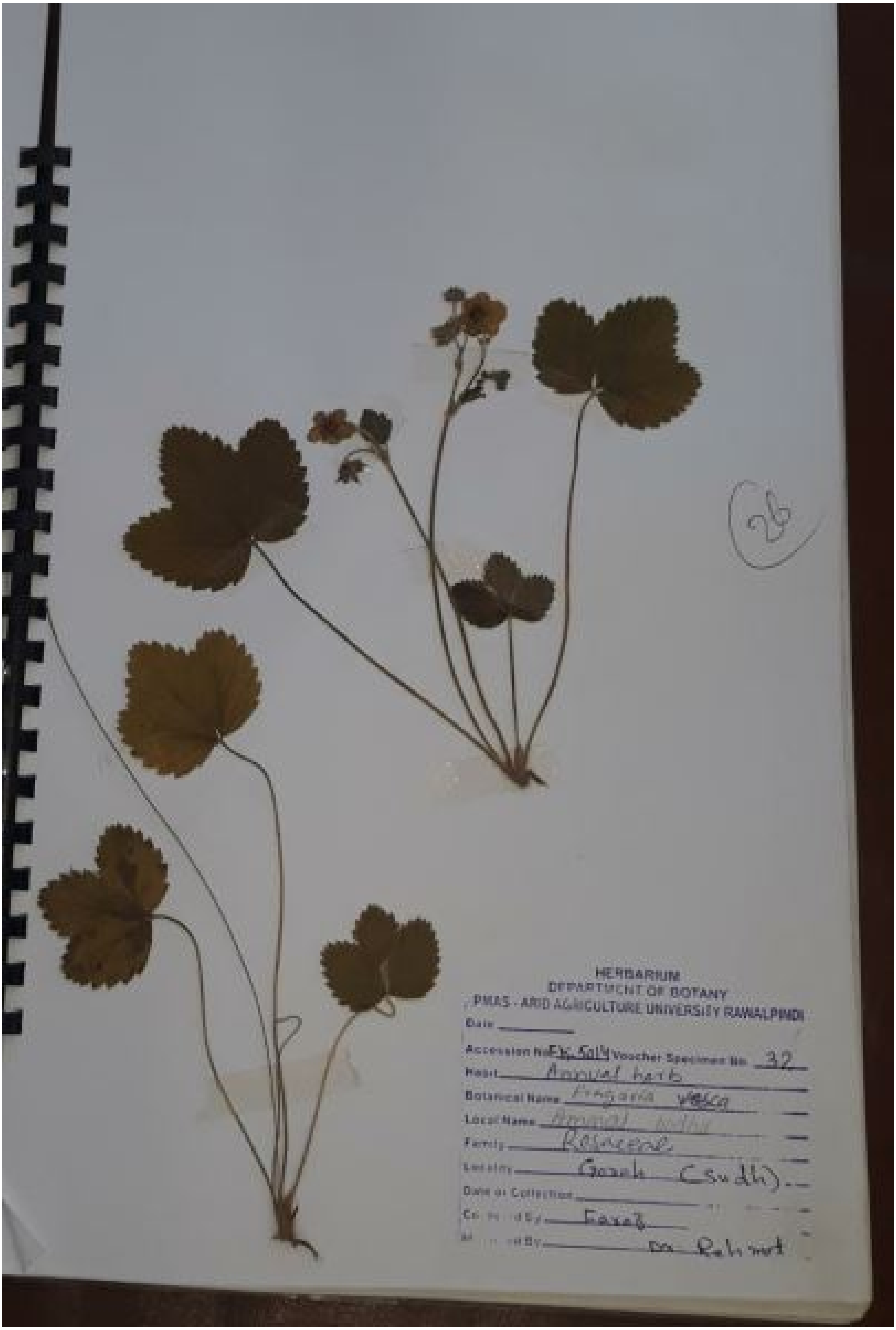
26
HERBARIUM
DEPARTMENT OF BOTANY
PMAS - ARID AGRICULTURE UNIVERSITY RAWALPINDI
Date
Voucher Specimen No. 32
Habit Annual herb
Botanical Name Fragaria vesca
Family Rosaceae
Locality Gozah (Sudh)
Date of Collection
Collected By Fazal
Dr. Rahmat

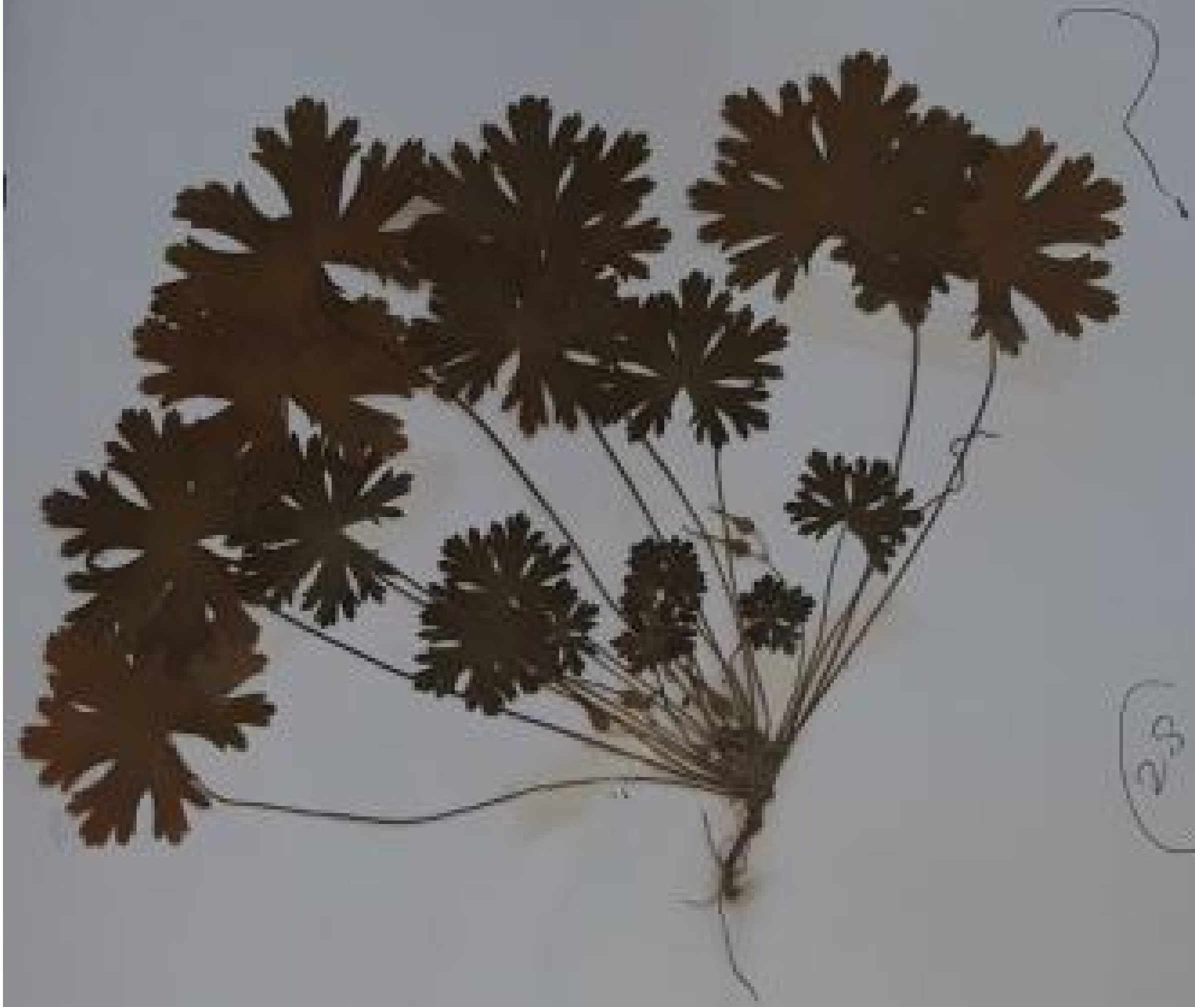

23

HERBARIUM
DEPARTMENT OF BOTANY
PMAS - ARID AGRICULTURE UNIVERSITY RAWALPINDI

Date ____________

Accession No. [illegible] Voucher Specimen No. 93

Habit Herb

Botanical Name Geranium wallichianum

Local Name [illegible]

Family Geraniaceae

Locality [illegible] Khal [illegible]

Date of Collection ____________

Collected By [illegible]

Identified By Dr. Rahmat [illegible]

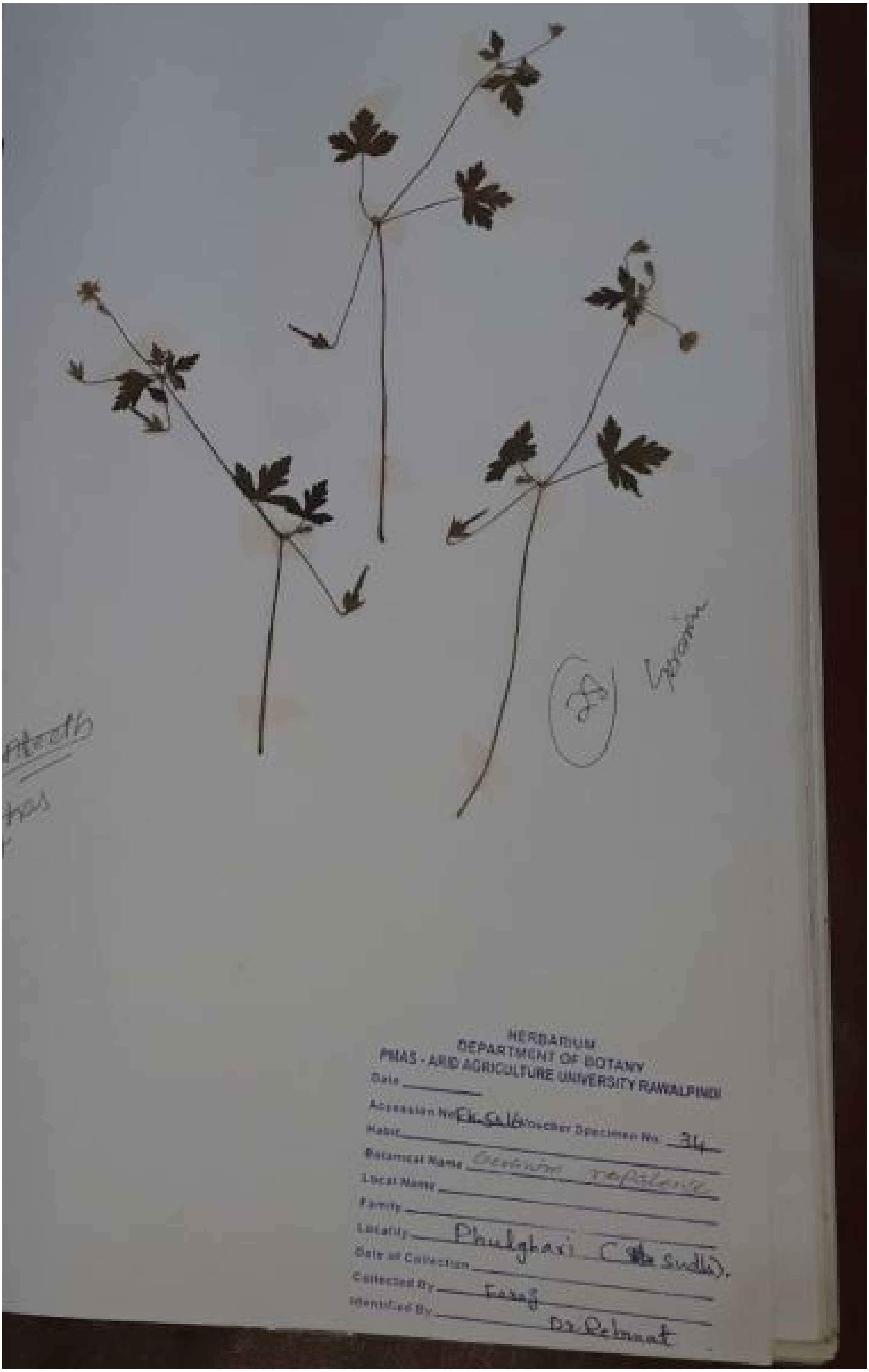
HERBARIUM
DEPARTMENT OF BOTANY
PMAS - ARID AGRICULTURE UNIVERSITY RAWALPINDI
Date
Accession No.
Voucher Specimen No. 34
Habit
Botanical Name Geranium nepalense
Local Name
Family
Locality Phulghari
Date of Collection
Collected By
Identified By Dr. Rehmat

EMORY UNIVERSITY
022197
HERBARIUM

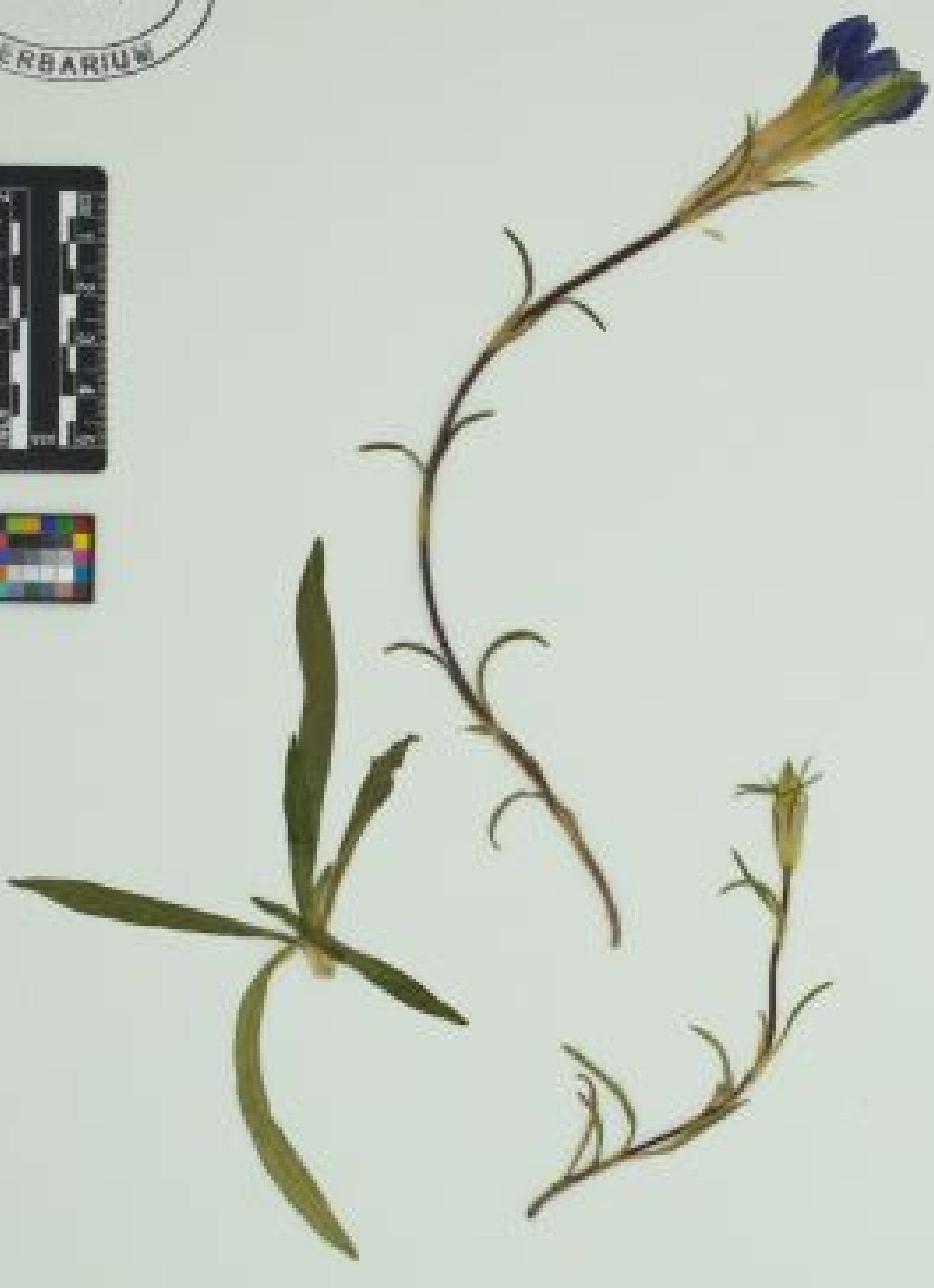

INCORPORATED INTO THE
MEDICAL BOTANY SPECIAL COLLECTION

FK-104

**EMORY UNIVERSITY HERBARIUM**
FLORA OF ISLAMIC REPUBLIC OF PAKISTAN

*Gentiana olivieri* Griseb. GENTIANACEAE

Goraih, Sudhanoti, Azad Kashmir, Islamic Republic of Pakistan.

common, dry wild habitat

flowers blue in color, showy

anti-infection, hypoglycemic, used for renal retention local name: Neel Kanth

Muhammad Faraz Khan FK-104 5 June 2017

Michelle Lin 25-September-2017

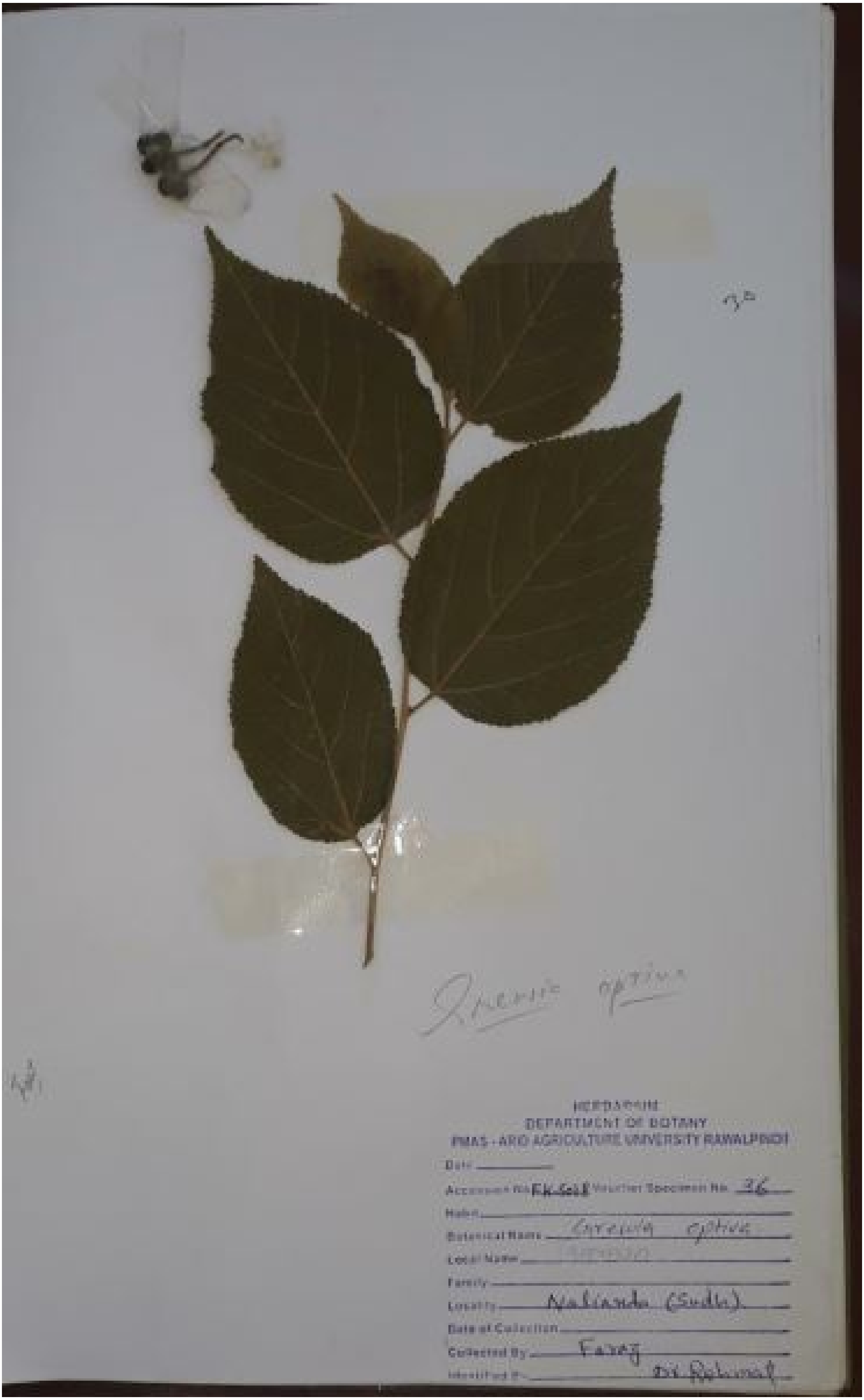
HERBARIUM
DEPARTMENT OF BOTANY
PMAS - ARID AGRICULTURE UNIVERSITY RAWALPINDI
Date
Accession No. Voucher Specimen No. 36
Habit
Botanical Name
Local Name
Family
Locality Nakianda (Sudh).
Date of Collection
Collected By Faraz
Identified By

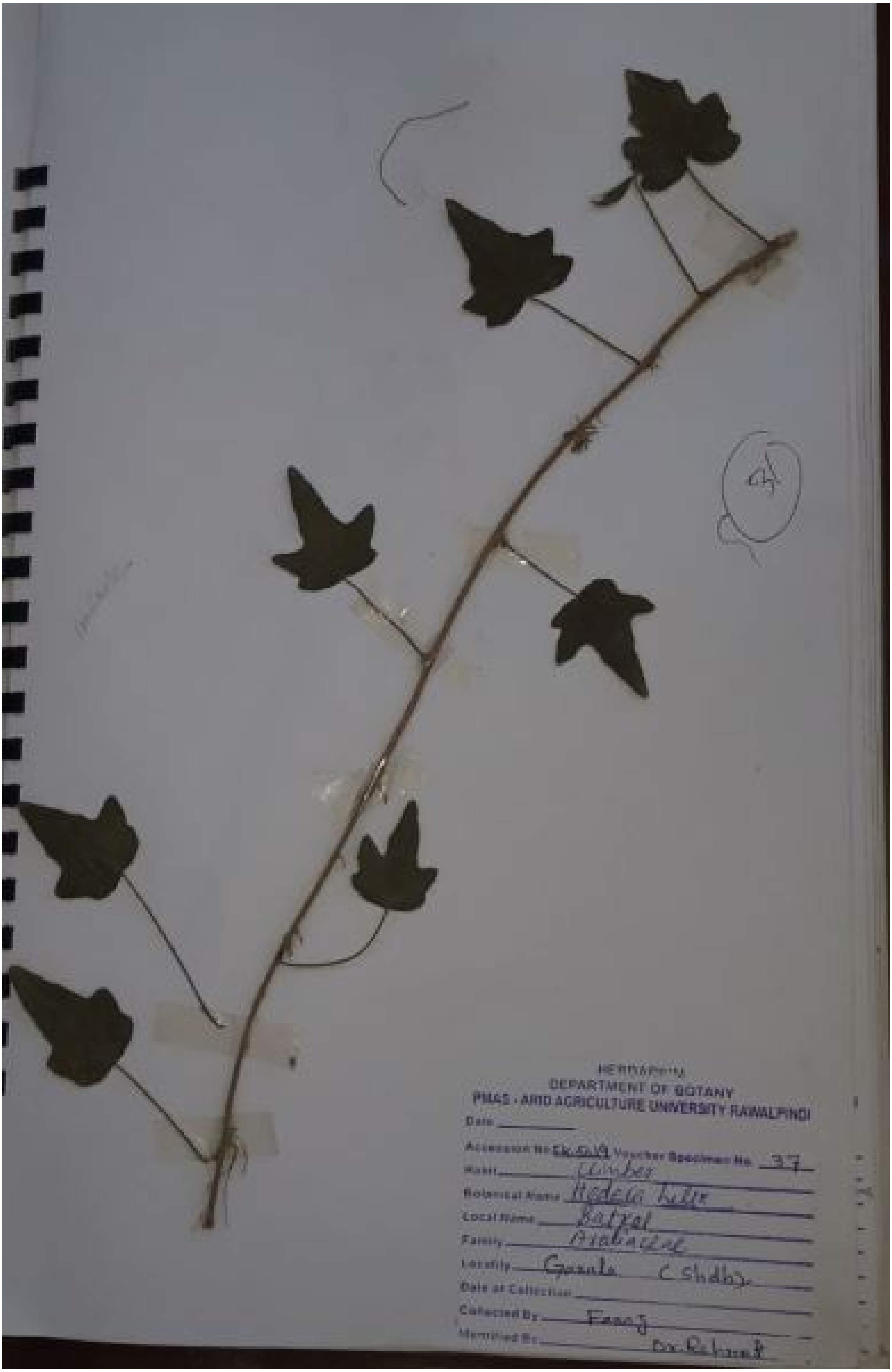
HERBARIUM
DEPARTMENT OF BOTANY
PMAS - ARID AGRICULTURE UNIVERSITY RAWALPINDI
Date
Accession No
Voucher Specimen No. 37
Habit
Botanical Name
Local Name
Family
Locality
Date of Collection
Collected By
Identified By

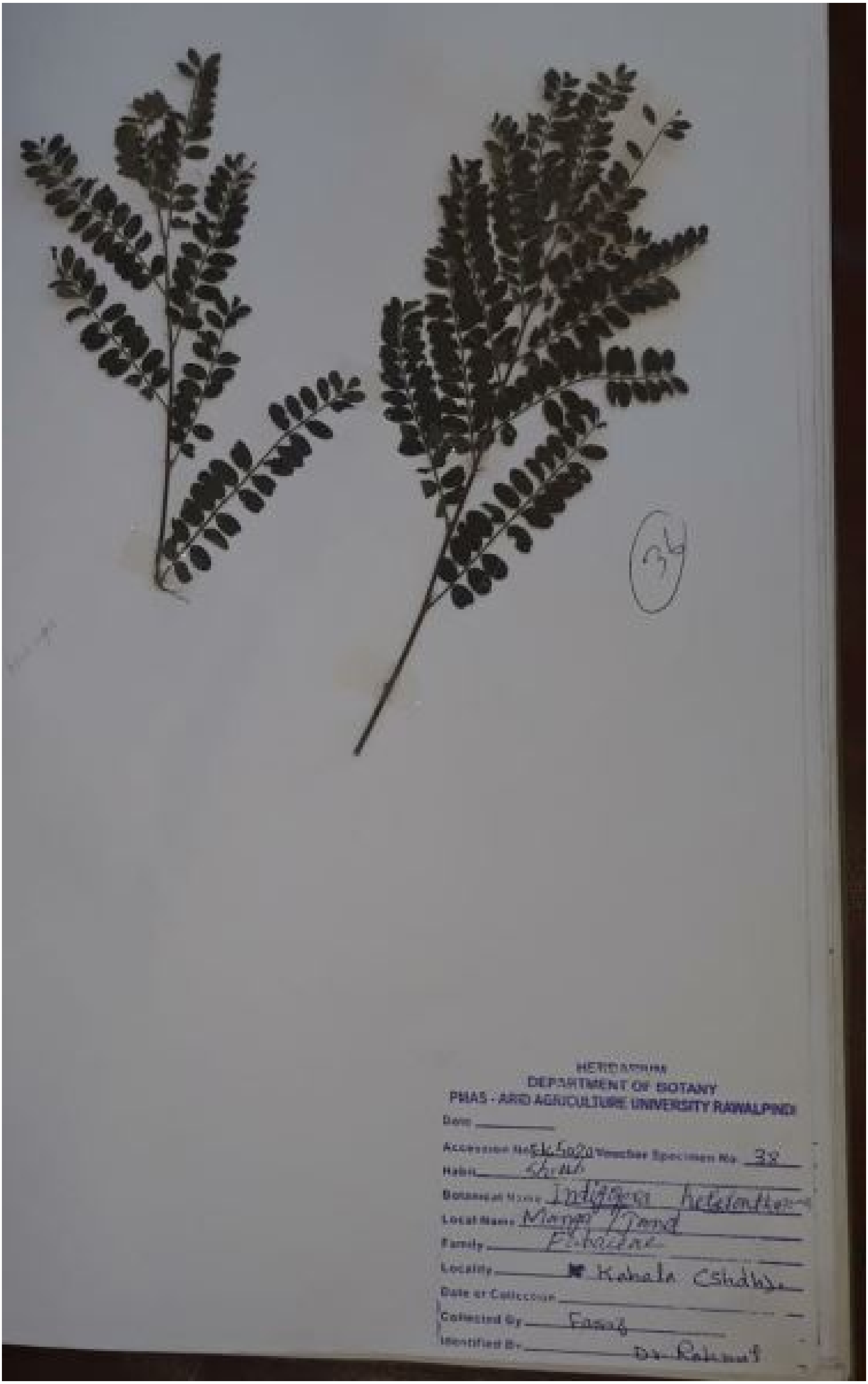
DEPARTMENT OF BOTANY
PMAS - ARID AGRICULTURE UNIVERSITY RAWALPINDI
Date
Accession No. Voucher Specimen No. 32
Habit Shrub
Botanical Name Indigofera heterantha
Local Name
Family Fabaceae
Locality
Date of Collection
Collected By
Identified By

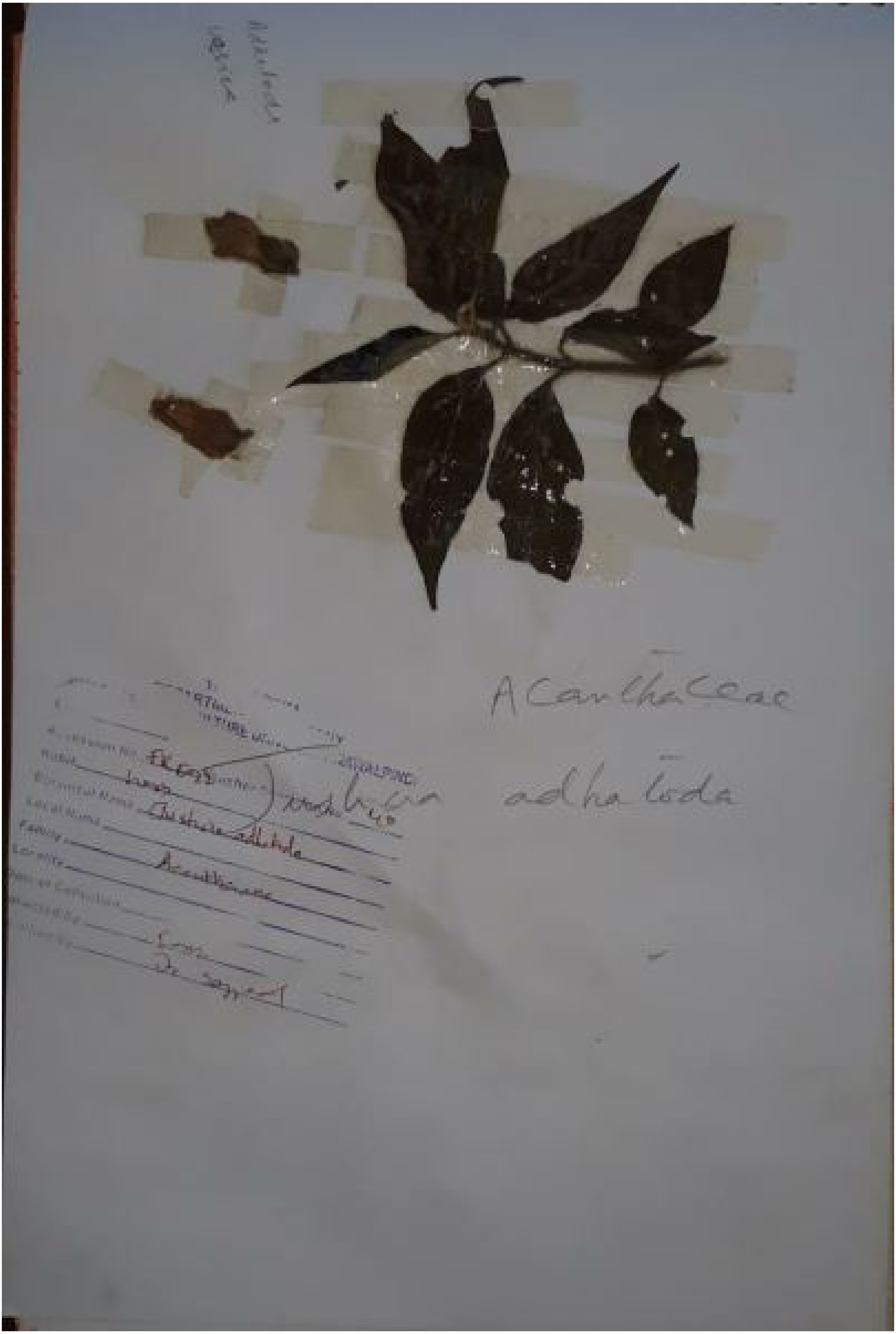
Acanthaceae
adhatoda

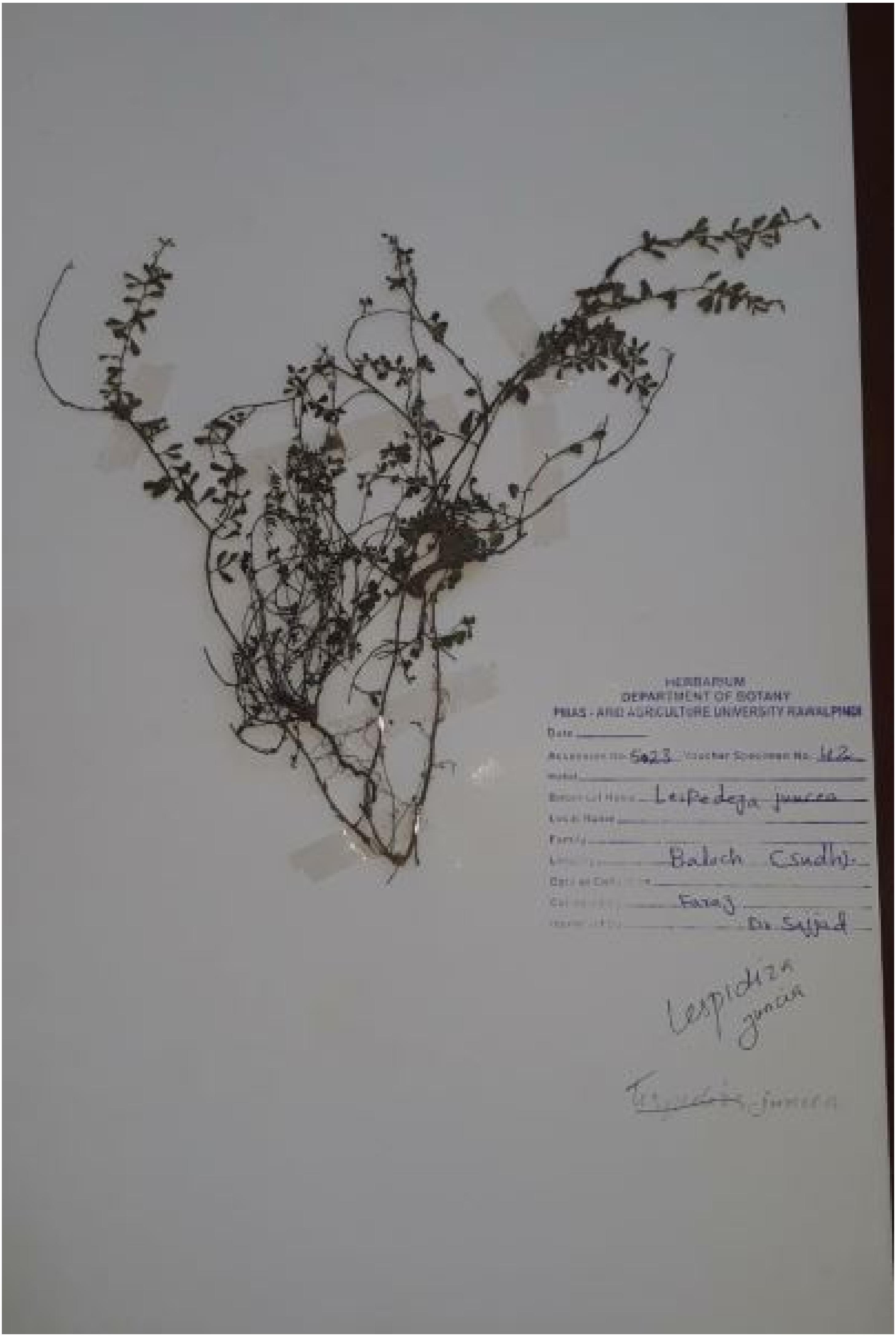
HERBARIUM
DEPARTMENT OF BOTANY
PMAS - ARID AGRICULTURE UNIVERSITY RAWALPINDI
Date
Accession No. 5023 Voucher Specimen No. 162
Lespedeza juncea
Family
Baloch (sudhi)
Faraz
Dr. Sajjad
Lespideza juncia

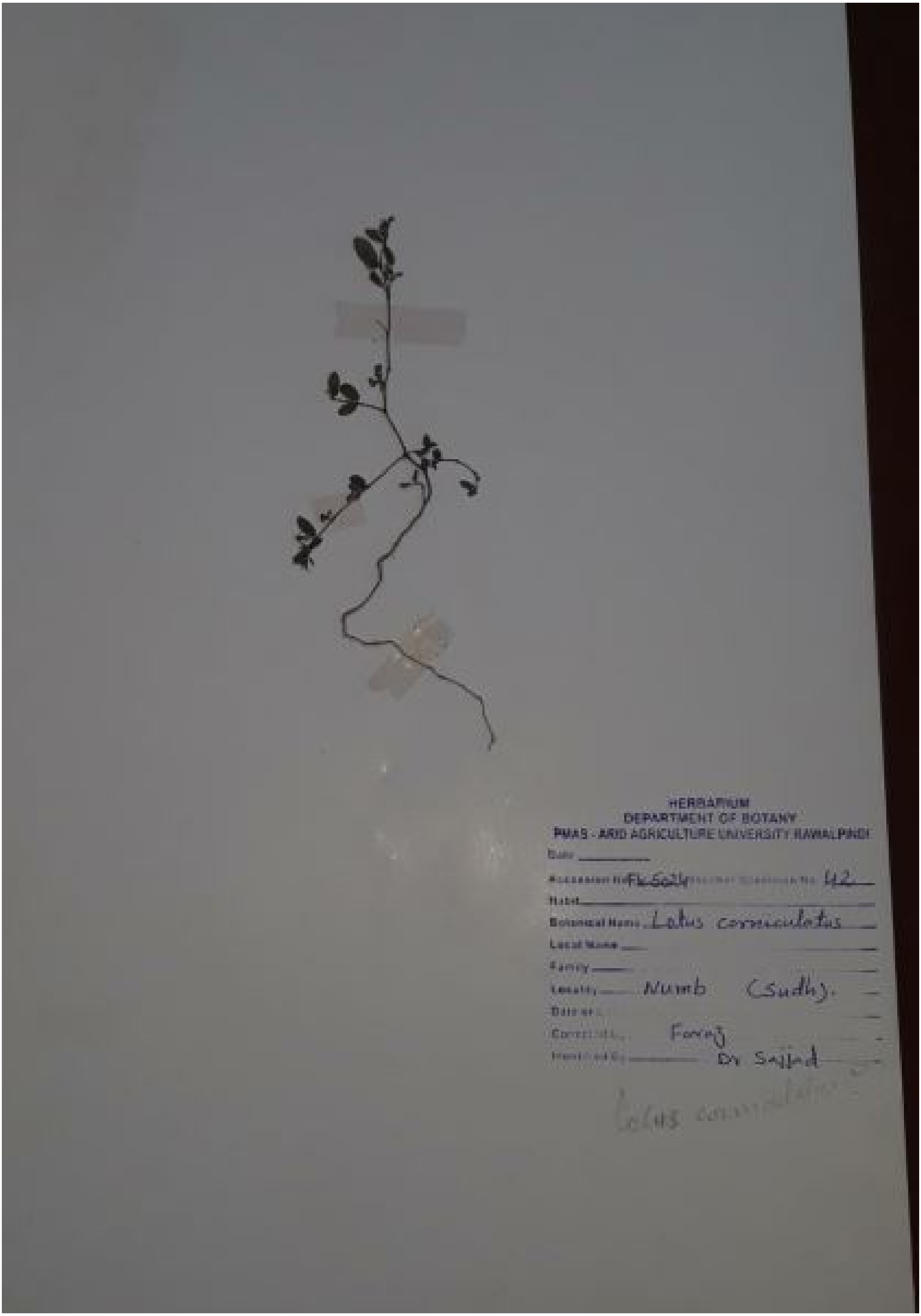
HERBARIUM
DEPARTMENT OF BOTANY
PMAS - ARID AGRICULTURE UNIVERSITY RAWALPINDI
Date
42
Botanical Name Lotus corniculatus
Local Name
Family
Numb (Sudh).
Farraj
Dr Sajjad

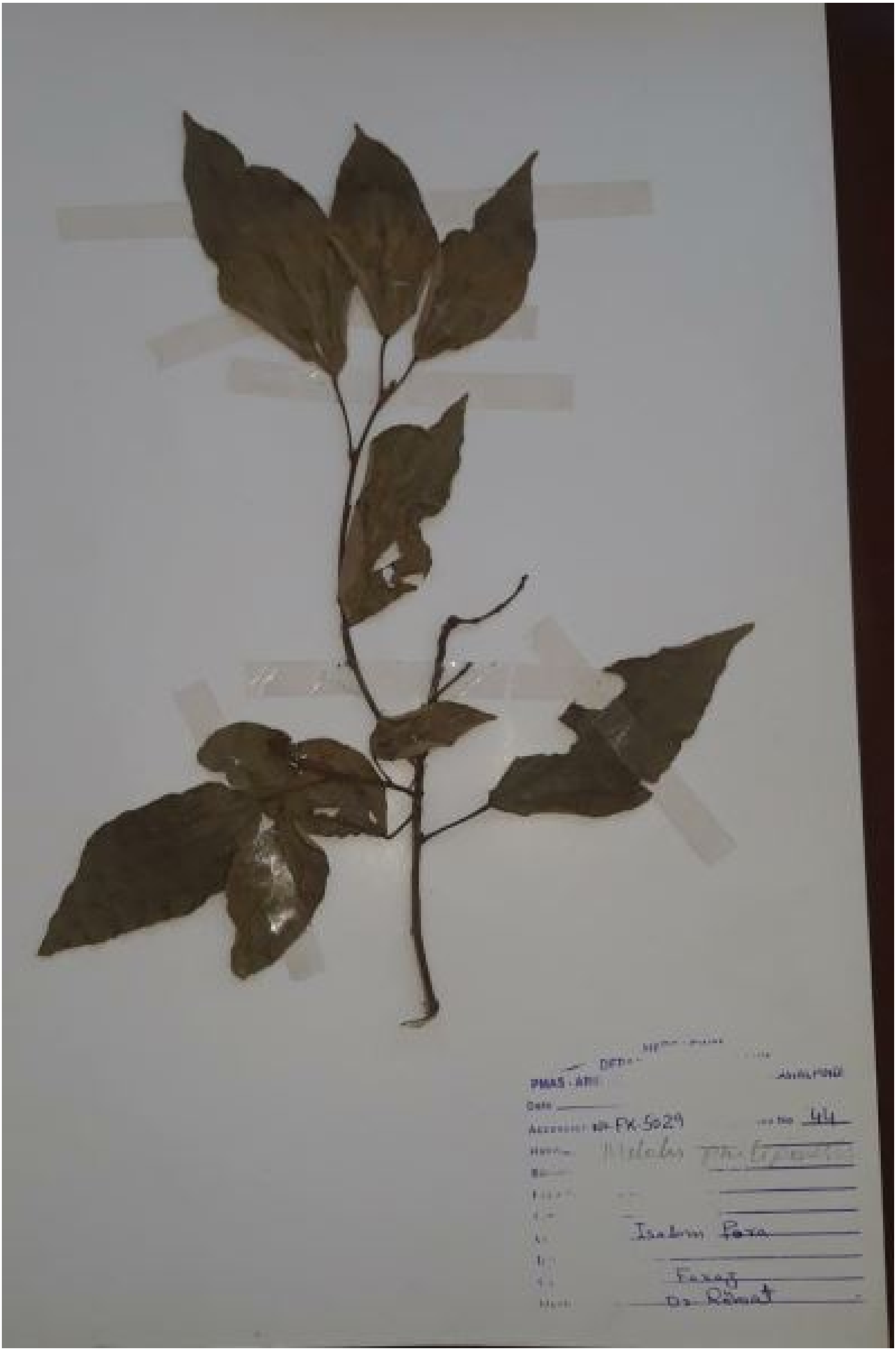
PMAS - AR
Date
FK-5029
44
Faraz

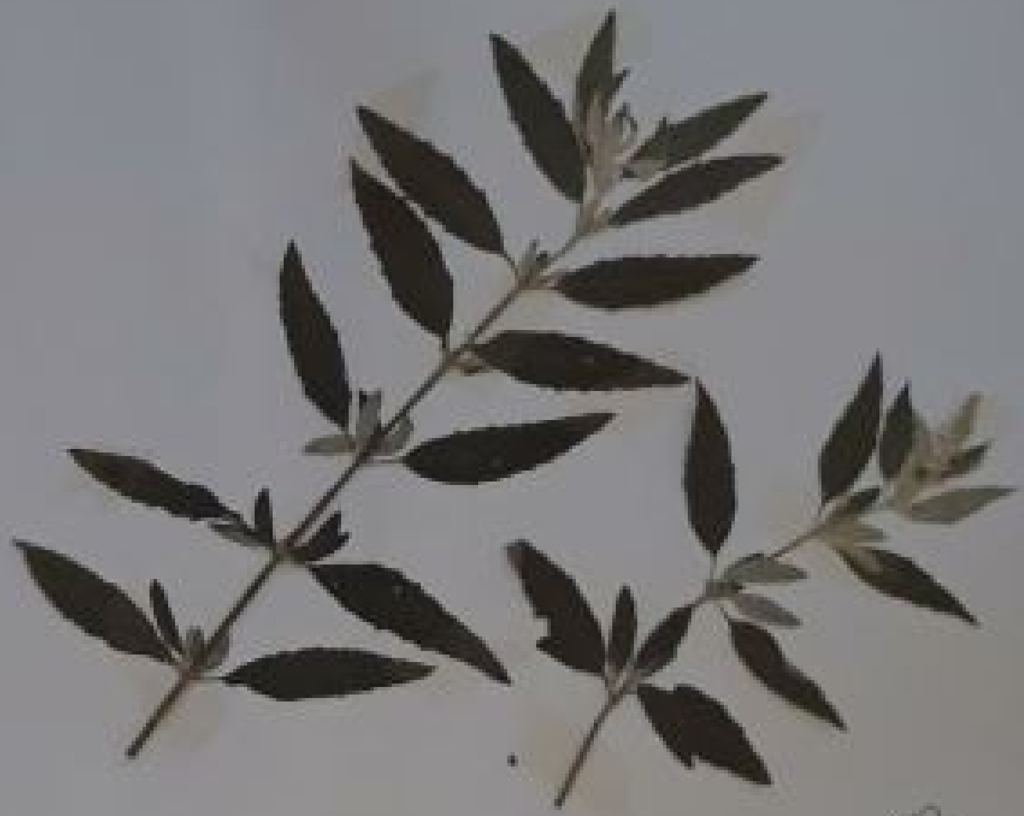

Mentha longifolia

HERBARIUM
DEPARTMENT OF BOTANY
PMAS - ARID AGRICULTURE UNIVERSITY RAWALPINDI

Date ________

Accession No. Rk.5031 Voucher Specimen No. 47

Habit: Herb

Botanical Name: Mentha longifolia L.

Local Name: Chotta [illegible]

Family: Lamiaceae

Locality: Baikan (Sindh)

Date of Collection: ________

Collected By: Faraz

Identified By: Dr. Rehmat

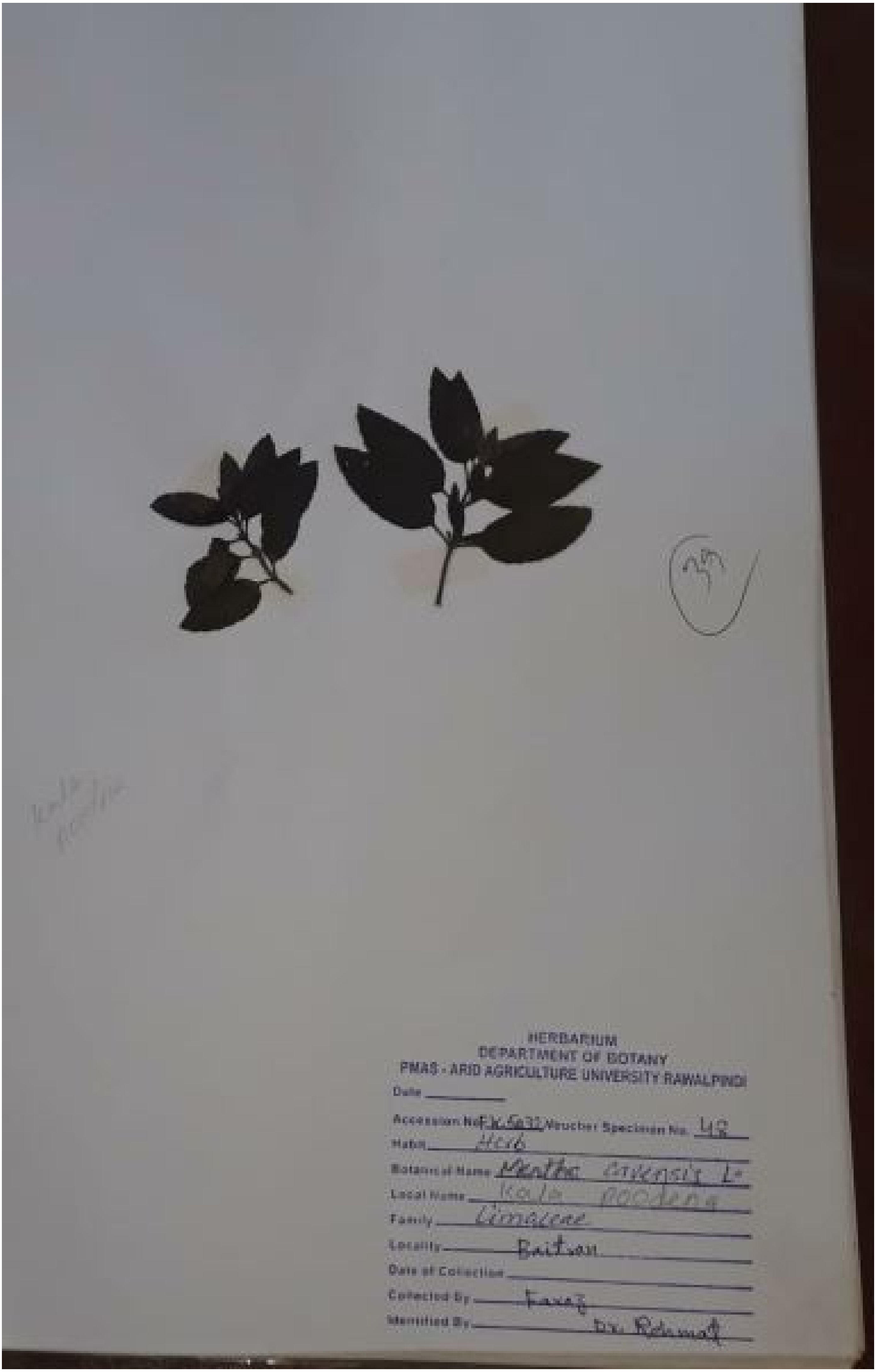
HERBARIUM
DEPARTMENT OF BOTANY
PMAS - ARID AGRICULTURE UNIVERSITY RAWALPINDI
Date
Voucher Specimen No. 42
Habit Herb
Botanical Name Mentha arvensis L.
Local Name Kala podena
Family Limaceae
Locality Baitsan
Date of Collection
Collected By Faraz
Identified By Dr. Rahmat

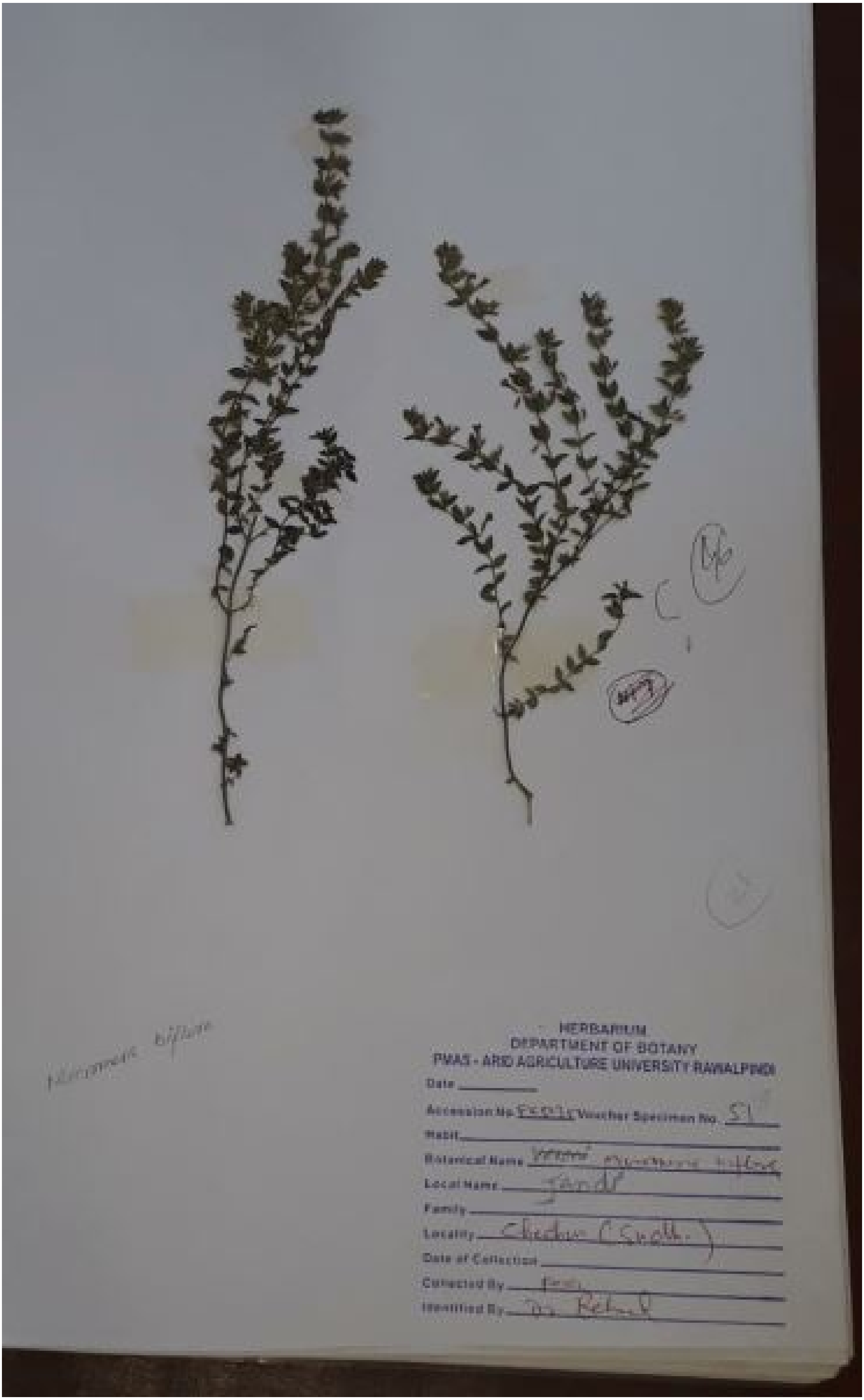
HERBARIUM
DEPARTMENT OF BOTANY
PMAS - ARID AGRICULTURE UNIVERSITY RAWALPINDI
Date
Accession No. Voucher Specimen No. 51
Habit
Botanical Name
Local Name
Family
Locality
Date of Collection
Collected By
Identified By

EMORY UNIVERSITY
022133
HERBARIUM

INCORPORATED INTO THE
MEDICAL BOTANY SPECIAL COLLECTION

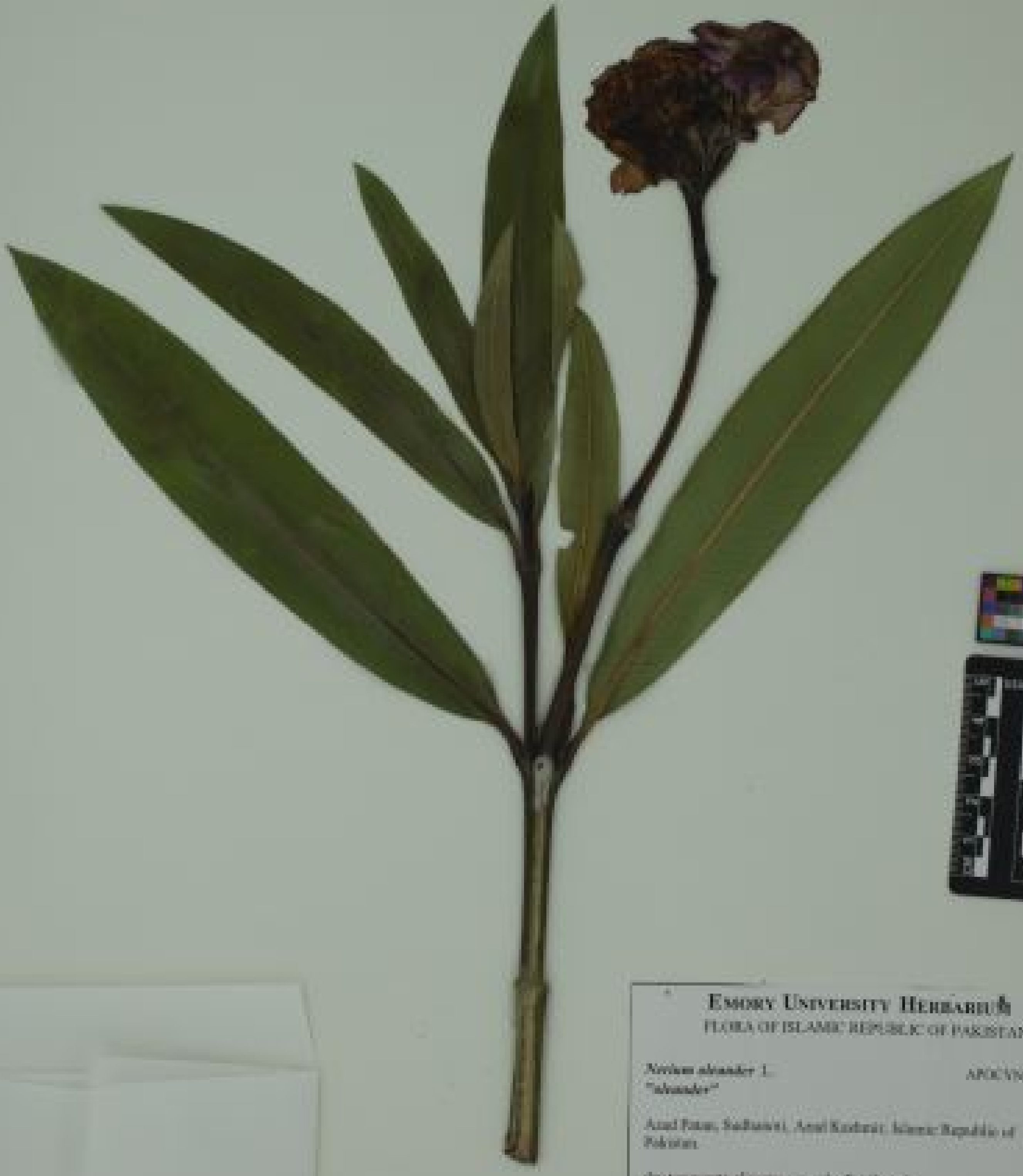

FR102

**EMORY UNIVERSITY HERBARIUM**
FLORA OF ISLAMIC REPUBLIC OF PAKISTAN

*Nerium oleander* L. — APOCYNACEAE
*"oleander"*

Azad Pattan, Sudhanoti, Azad Kashmir, Islamic Republic of Pakistan.

dry temperate climate; sparsely distributed

shrub, flowers conspicuous pink; plants with green leaves bitter in taste.

Used for toothache as a stick brush to cure infection and pain; powder used for skin problems local name: Kaneera

Muhammad Faraz Khan PK-182 — 13 July 2017

Emory University Herbarium

[illegible]

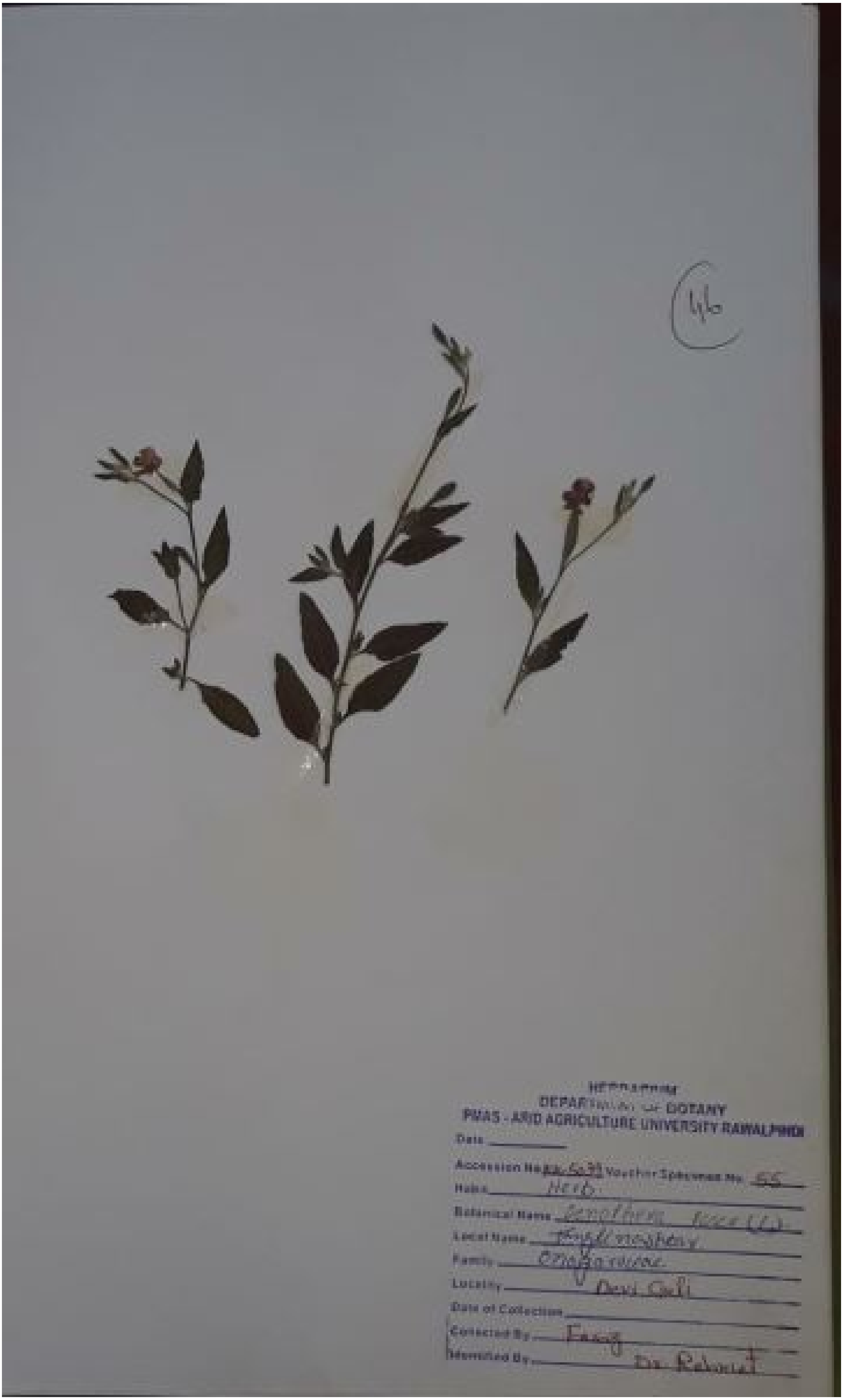

46
DEPARTMENT OF BOTANY
PMAS - ARID AGRICULTURE UNIVERSITY RAWALPINDI
Date
Accession No.
Voucher Specimen No. 55
Habit Herb.
Botanical Name Oenothera rosea (L.)
Local Name
Family Onagraceae
Locality Devi Gali
Date of Collection
Collected By
Identified By Dr. Rahmat

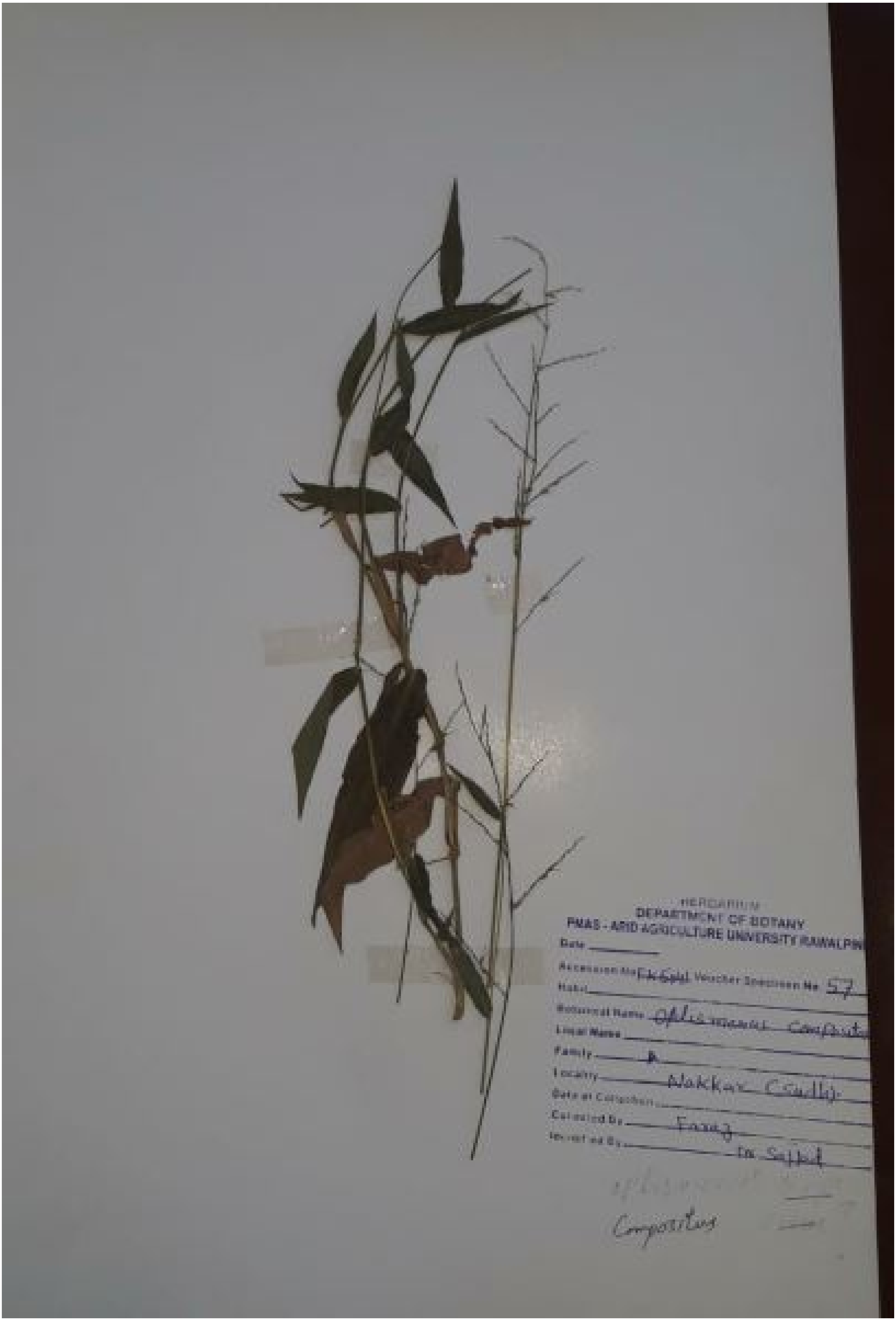
HERBARIUM
DEPARTMENT OF BOTANY
PMAS - ARID AGRICULTURE UNIVERSITY RAWALPI
Date
Voucher Specimen No 57
Botanical Name
Local Name
Family
Locality Nakkar
Date of Collection
Collected By Fazaj
Compositus

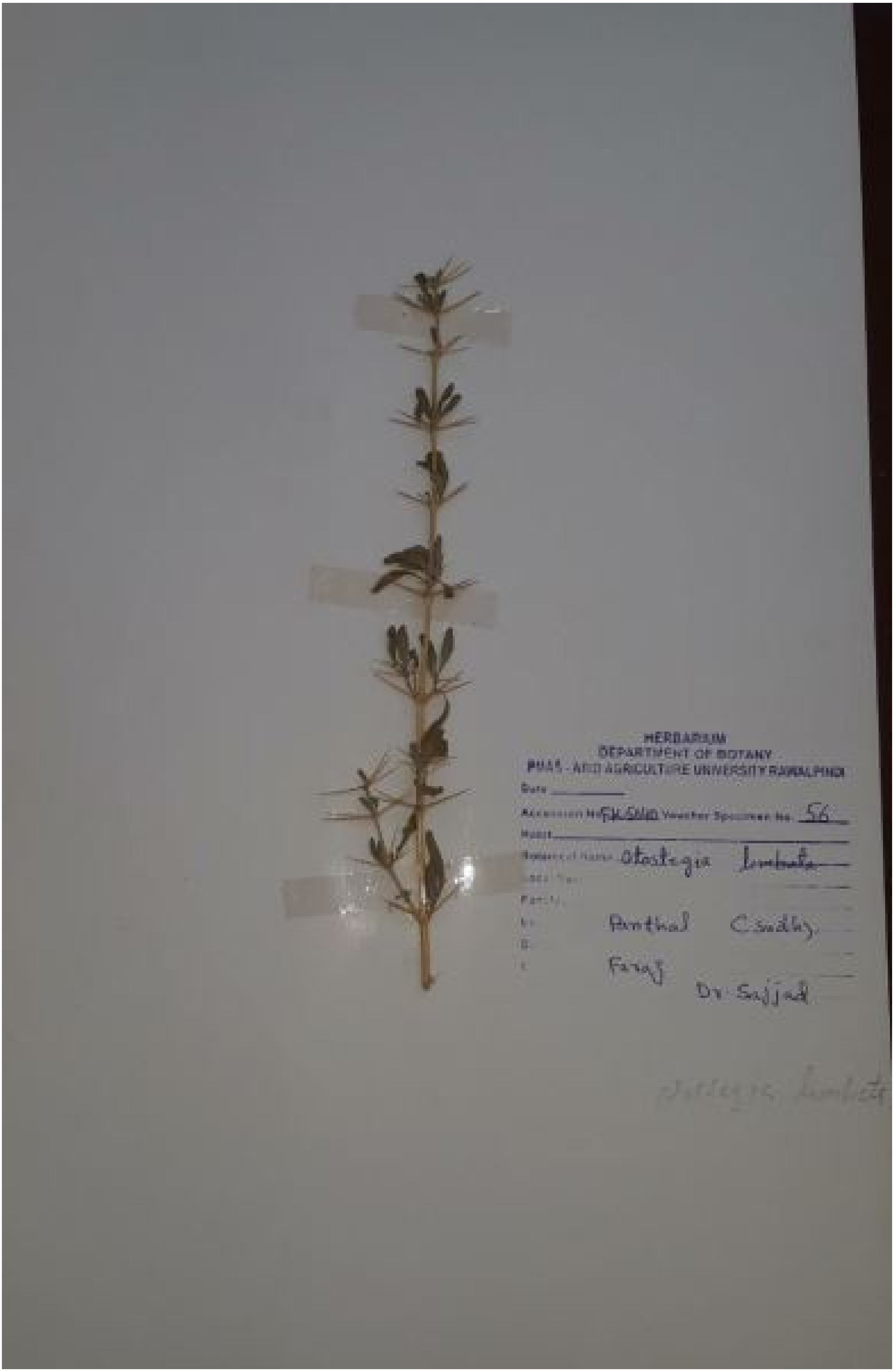
HERBARIUM
DEPARTMENT OF BOTANY
PMAS - ARID AGRICULTURE UNIVERSITY RAWALPINDI
Date
Voucher Specimen No. 56
Otostegia
Panthal
Faraj
Dr. Sajjad

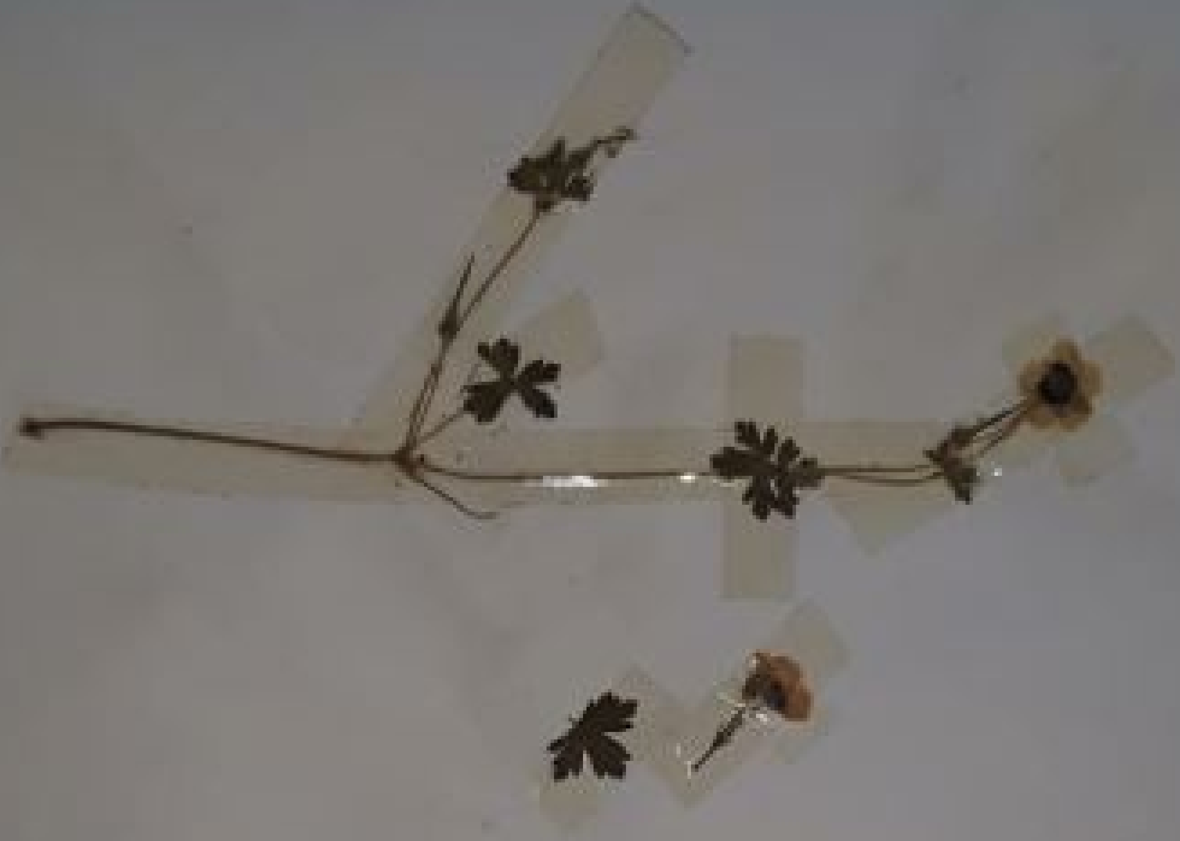

Geraniaceae

Geranium [illegible]

HERBARIUM
[illegible] DEPA[illegible] BOTANY
[illegible]ULTURE [illegible] RAWALPINDI

[illegible]ession No. [illegible] Voucher Specimen No. [illegible]
Habit
Botanical Name [illegible]
Local Name
Family
Locality [illegible] ([illegible])
Date of Collection
Collected By [illegible]
Identified by [illegible]

~~Ranunculus~~
~~Ranunculaceae~~

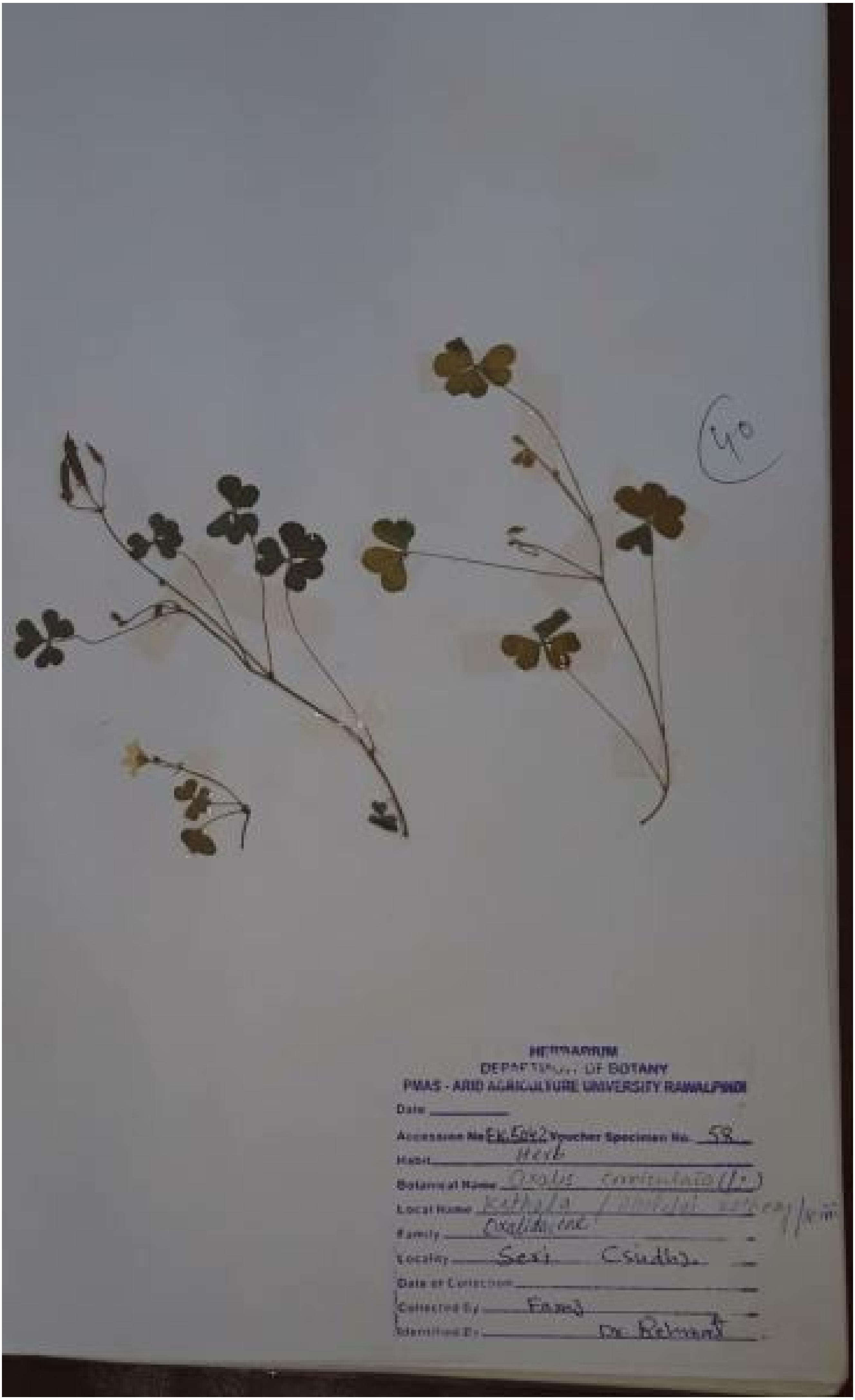
DEPARTMENT OF BOTANY
PMAS - ARID AGRICULTURE UNIVERSITY RAWALPINDI
Date
Accession No. EK 5042 Voucher Specimen No. 58
Habit Herb
Botanical Name Oxalis corniculata (L.)
Local Name
Family Oxalidaceae
Locality
Date of Collection
Collected By
Identified By Dr. Rehman

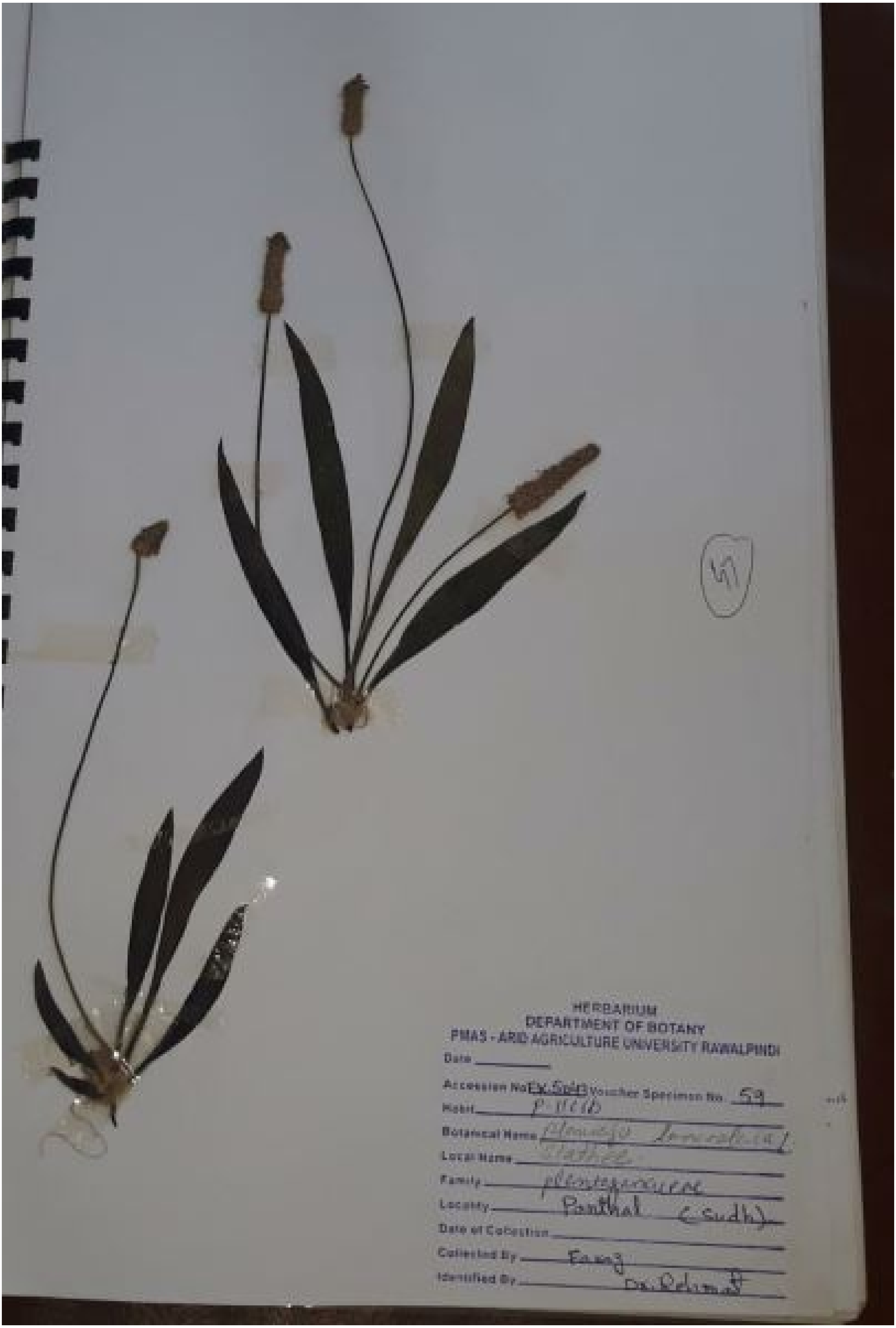
HERBARIUM
DEPARTMENT OF BOTANY
PMAS - ARID AGRICULTURE UNIVERSITY RAWALPINDI
Date
Accession No. Voucher Specimen No. 59
Habit
Botanical Name
Local Name
Family
Locality Panthal (Sudh)
Date of Collection
Collected By
Identified By

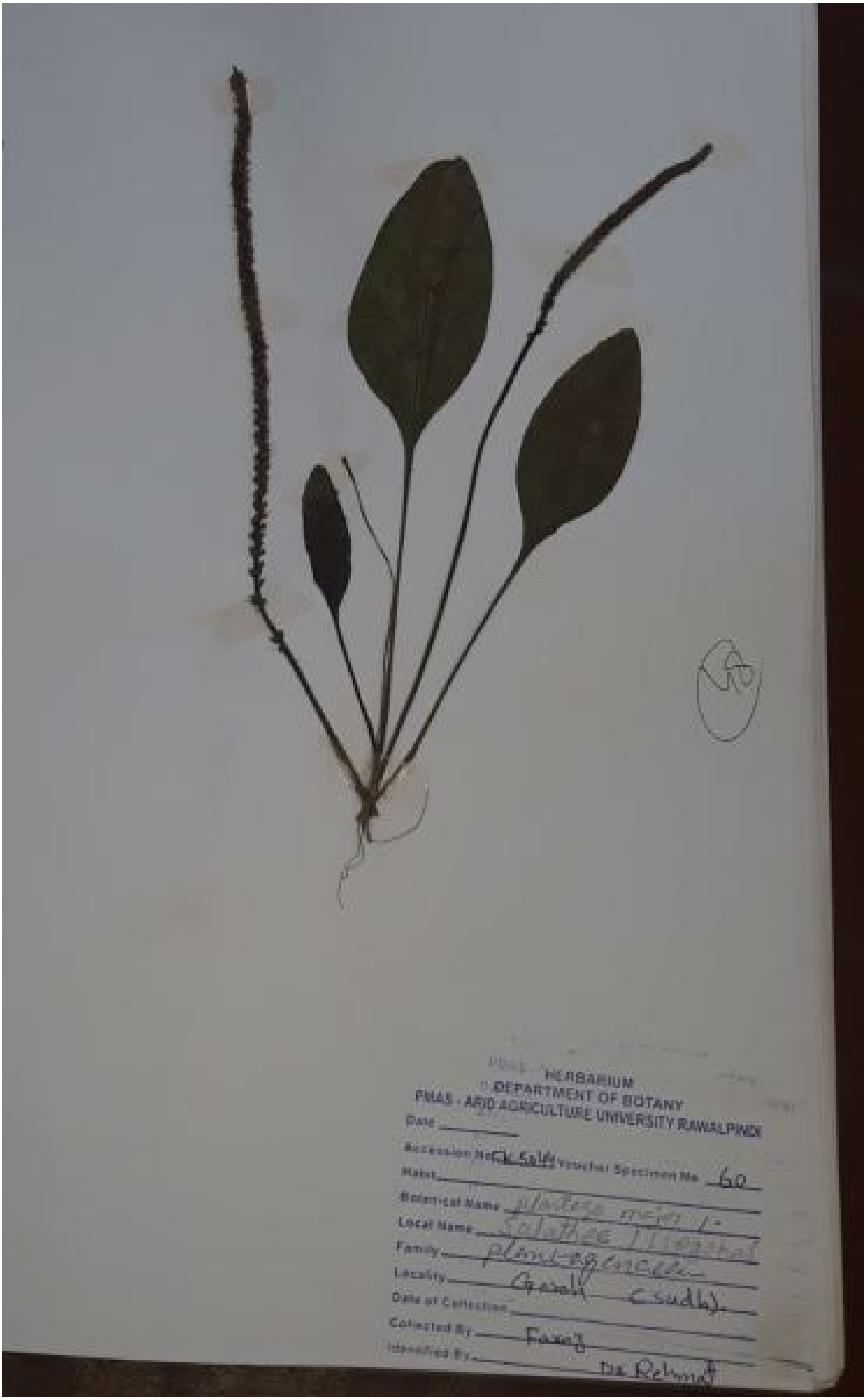
HERBARIUM
DEPARTMENT OF BOTANY
PMAS - ARID AGRICULTURE UNIVERSITY RAWALPINDI
Date
Accession No.
Voucher Specimen No. 60
Habit
Botanical Name
Local Name
Family
Locality
Date of Collection
Collected By
Identified By

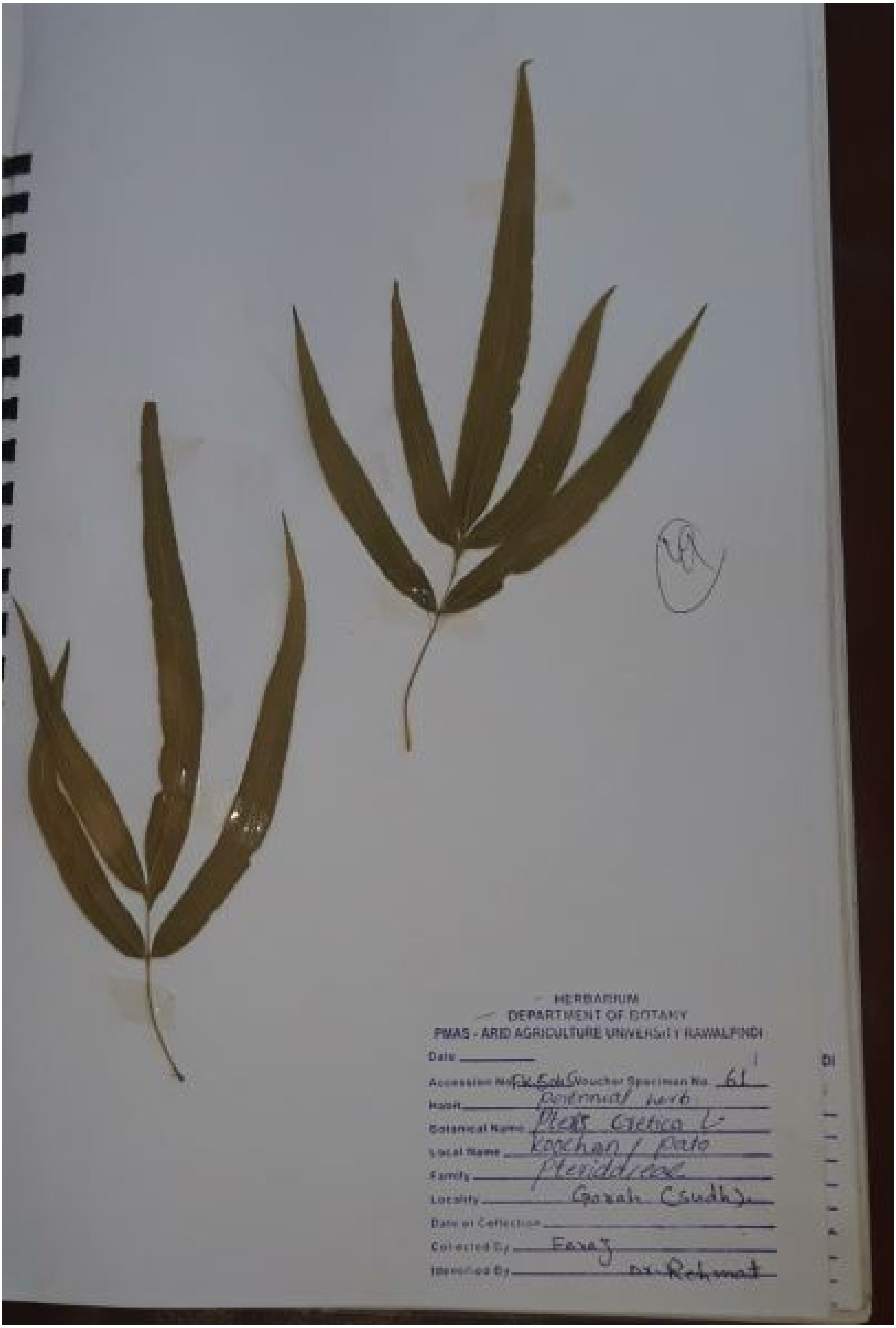
HERBARIUM
DEPARTMENT OF BOTANY
PMAS - ARID AGRICULTURE UNIVERSITY RAWALPINDI
Date
Accession No. Voucher Specimen No. 61
Habit perennial herb
Botanical Name Pteris cretica L.
Local Name Koochan / pato
Family Pteridaceae
Locality Gozah (Sudh)
Date of Collection
Collected By Faraz
Identified By Dr. Rehmat

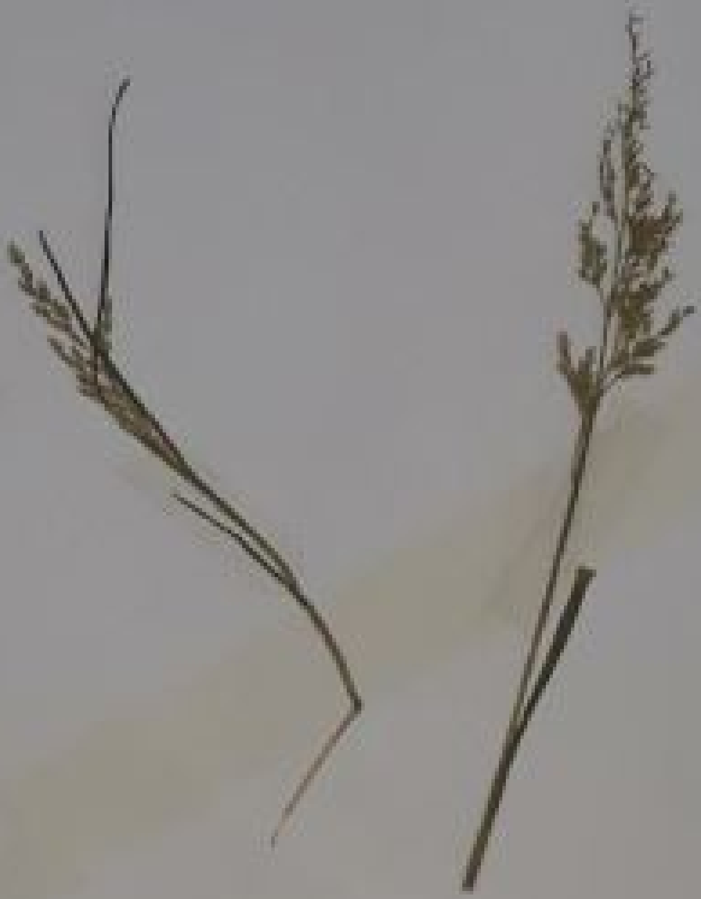

50

HERBARIUM
DEPARTMENT OF BOTANY
PMAS - ARID AGRICULTURE UNIVERSITY RAWALPINDI

Date ________

Accession No. PK-50[illegible] Voucher Specimen No. 62

Habit ________

Botanical Name Poa annua L.

Local Name [illegible] / malla

Family Poaceae

Locality Gorah (Sudh).

Date of Collection ________

Collected By Faraz

Identified By Dr. Rahmat

51

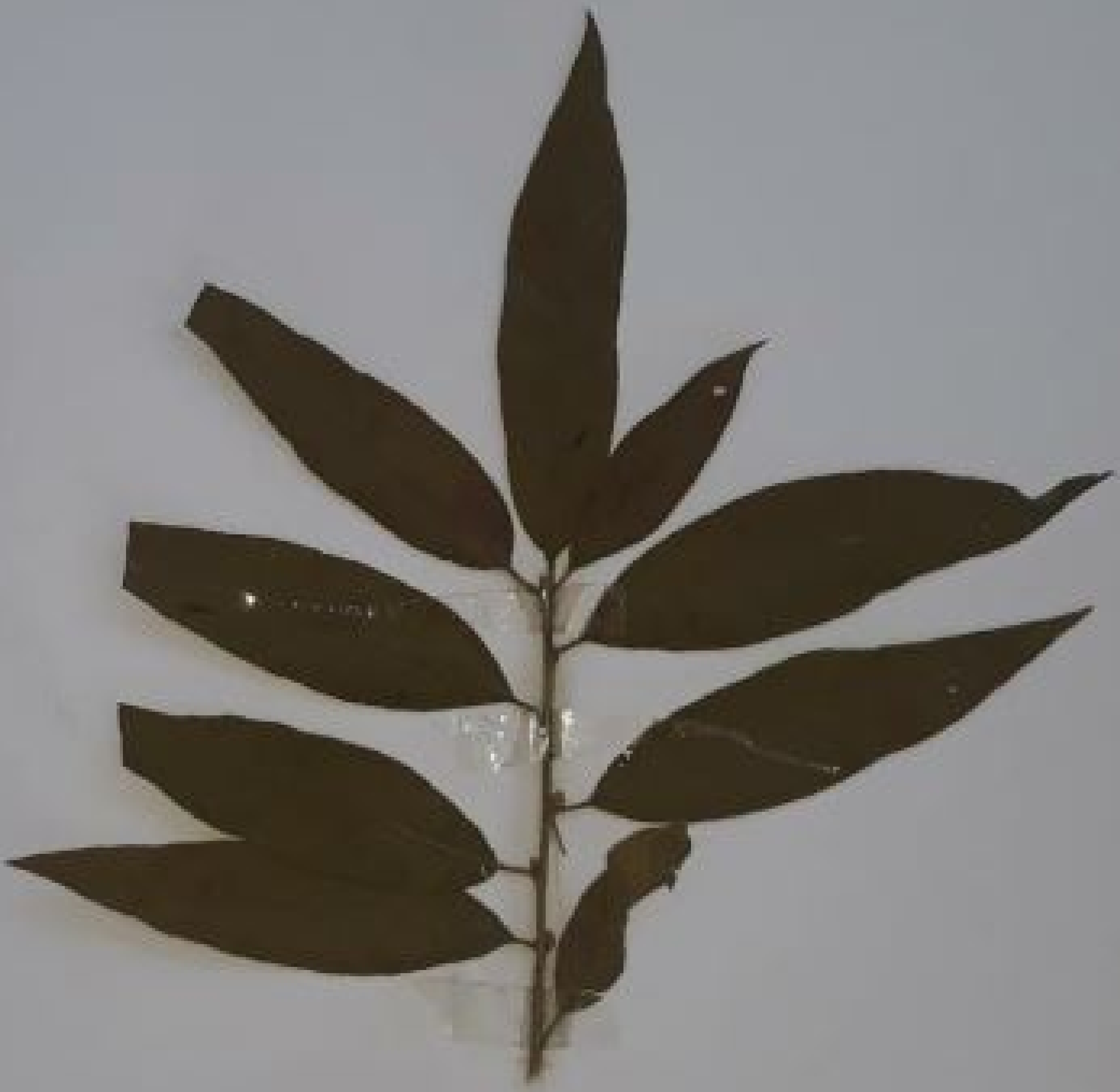

HERBARIUM
DEPARTMENT OF BOTANY
PMAS - ARID AGRICULTURE UNIVERSITY RAWALPINDI

Date ________

Accession No. [illegible] Voucher Specimen No. 63

Habit Tree

Botanical Name prunus persica

Local Name [illegible]

Family Rosaceae

Locality [illegible] ([illegible])

Date of Collection ________

Collected By [illegible]

Identified By Dr. [illegible]

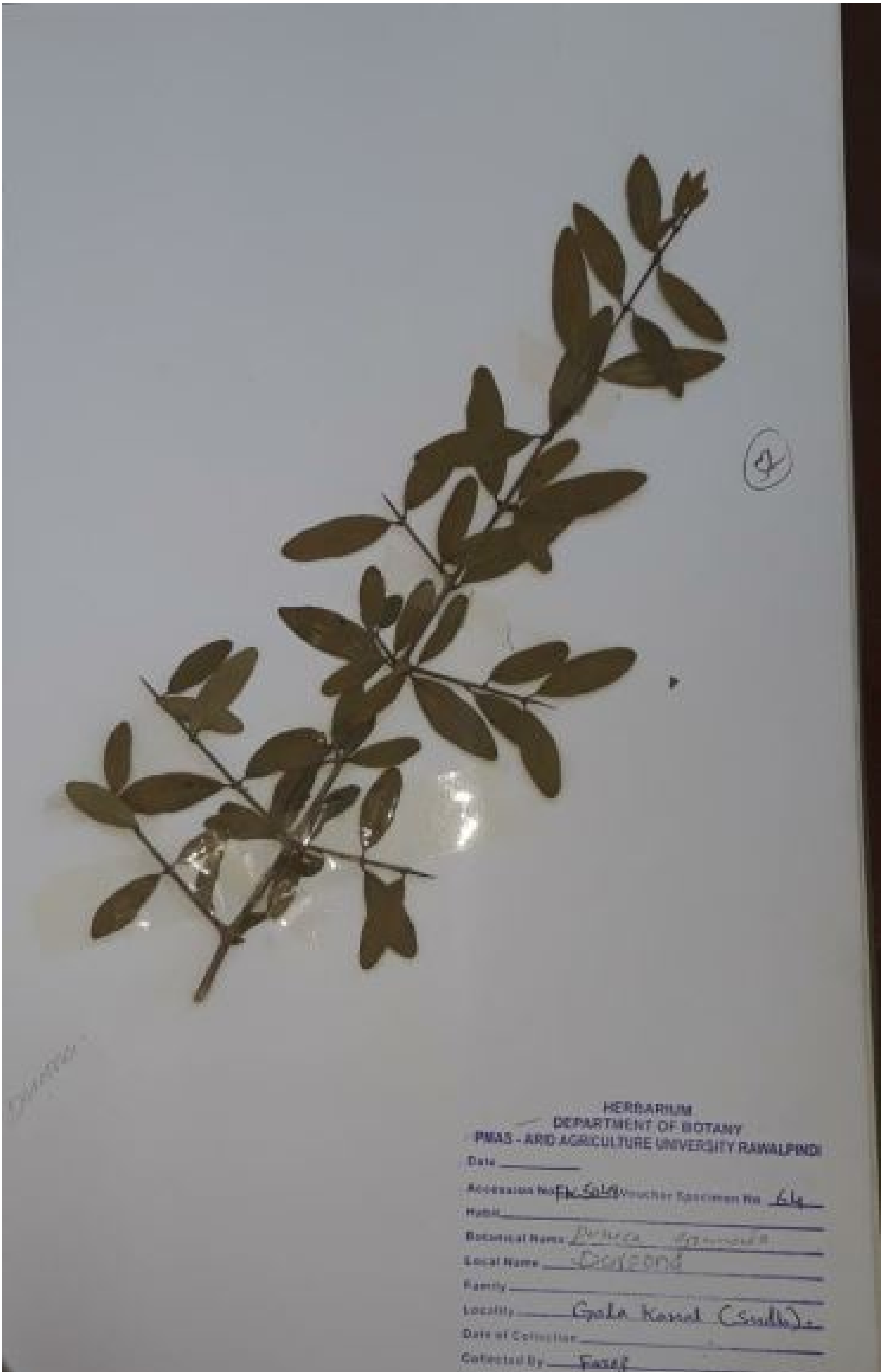
HERBARIUM
DEPARTMENT OF BOTANY
PMAS - ARID AGRICULTURE UNIVERSITY RAWALPINDI
Date
Accession No
Voucher Specimen No
Habit
Botanical Name
Local Name
Family
Locality
Date of Collection
Collected By
Identified By

Incorporated into the
Medical Botany Special Collection

EMORY UNIVERSITY HERBARIUM
024200

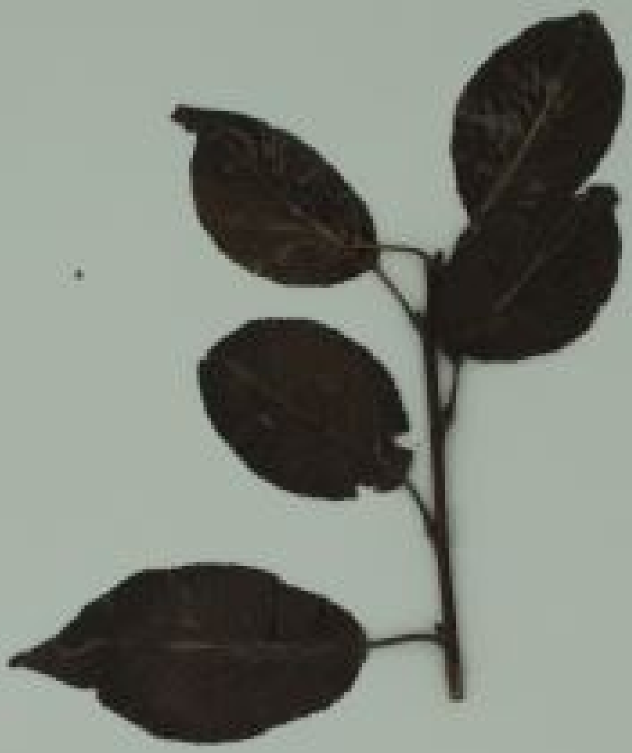

FK-101

## Emory University Herbarium
FLORA OF ISLAMIC REPUBLIC OF PAKISTAN

*Pyrus pashia* Buch.-Ham. ex D. Don ROSACEAE
*"wild Himalayan pear"*

Gorah, Sudhanoti, Azad Kashmir, Islamic Republic of Pakistan

moist temperate

tree, flowers white, fruits edible, round in shape

fruits edible, young shoots are ground into paste and use to cure psoriasis and skin infections; fruits are ground, dried used for liver problems, Hepatitis B local name: tangi

Muhammad Faraz Khan FK-101 13 July 2015

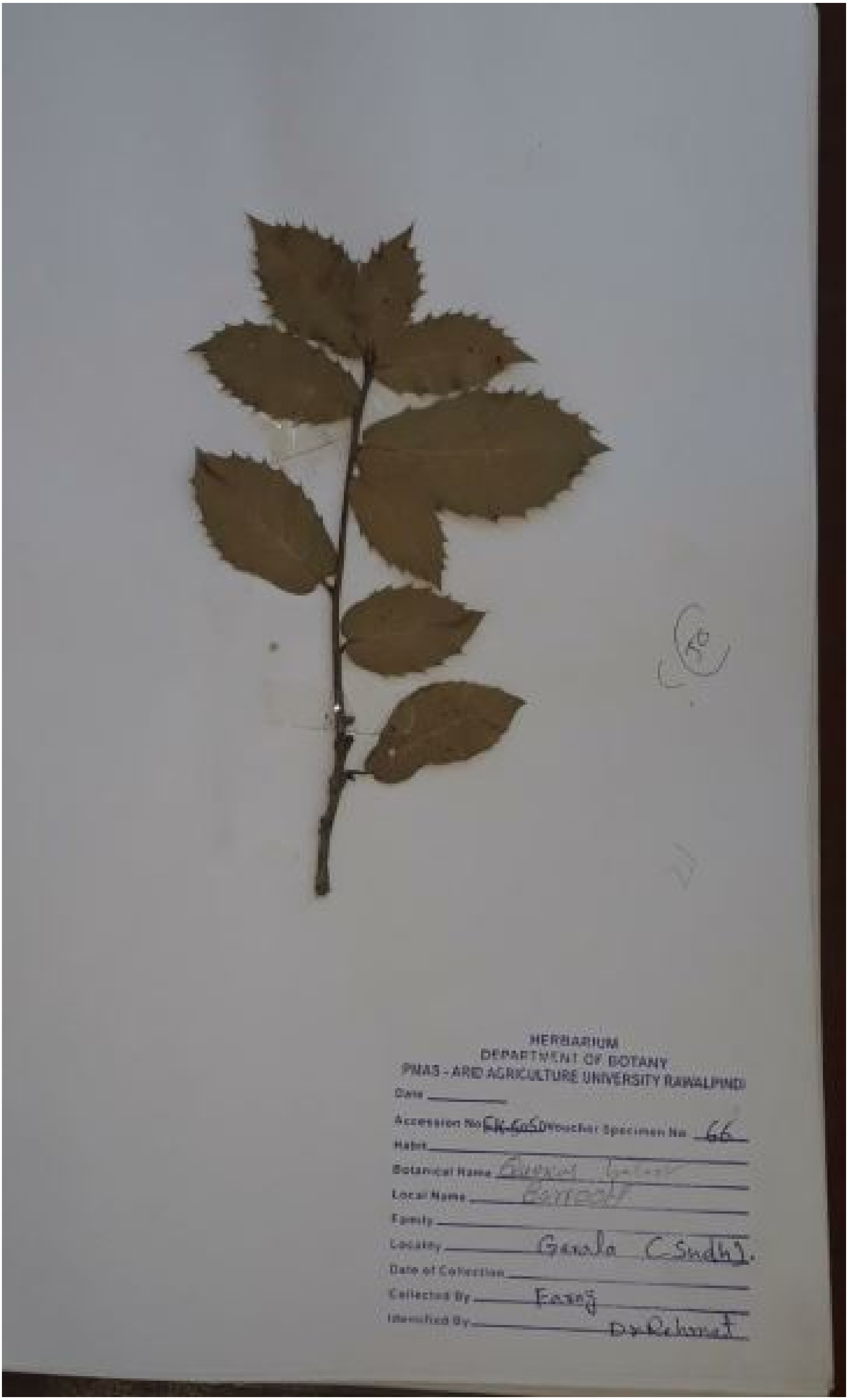
HERBARIUM
DEPARTMENT OF BOTANY
PMAS - ARID AGRICULTURE UNIVERSITY RAWALPINDI
Date
Accession No
Voucher Specimen No 66
Habit
Botanical Name
Local Name
Family
Locality Garala (Sudh).
Date of Collection
Collected By Faraz
Identified By Dr Rehmat

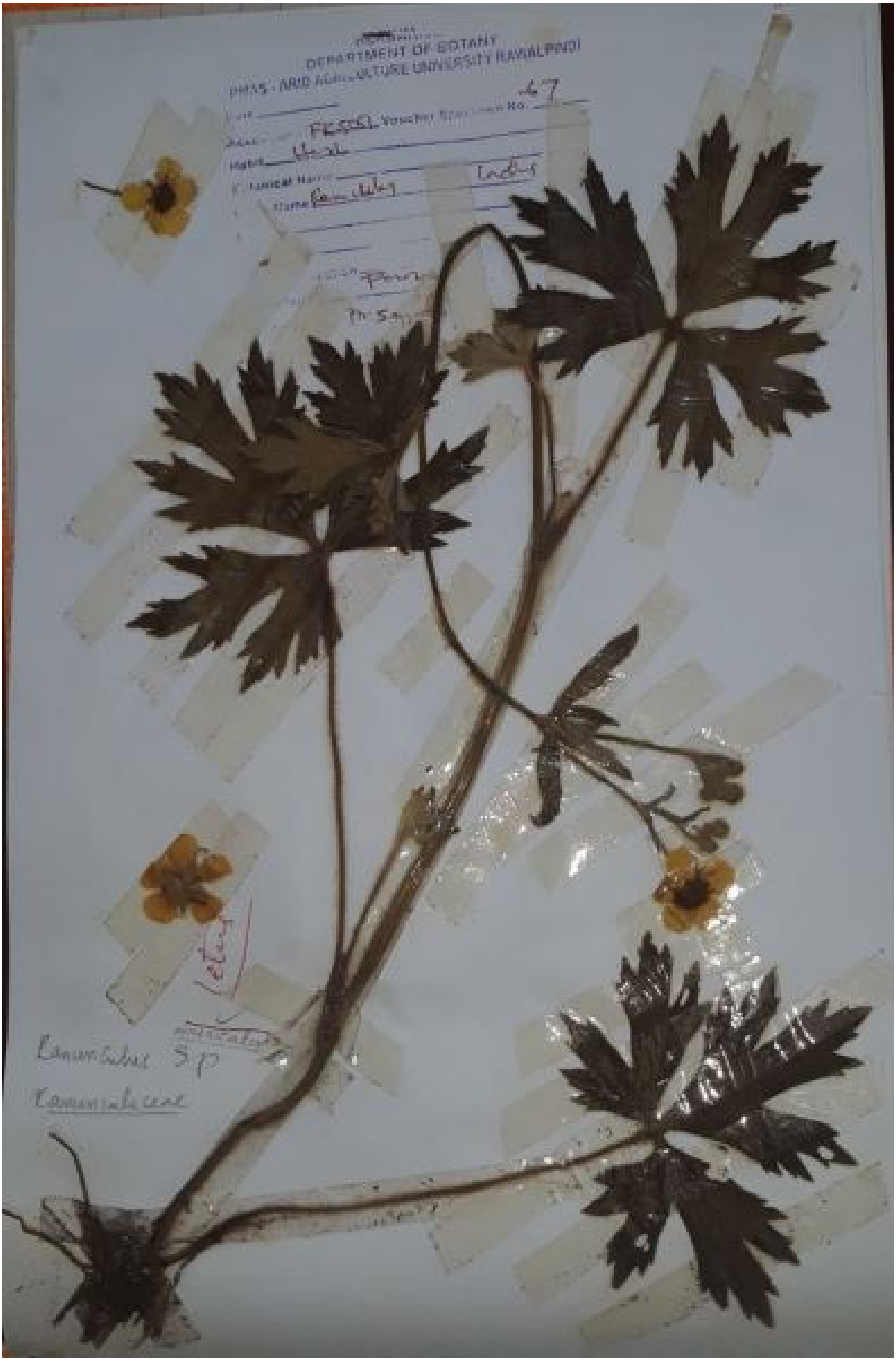
DEPARTMENT OF BOTANY
PMAS - ARID AGRICULTURE UNIVERSITY RAWALPINDI
67
Ranunculus Sp
Ranunculaceae

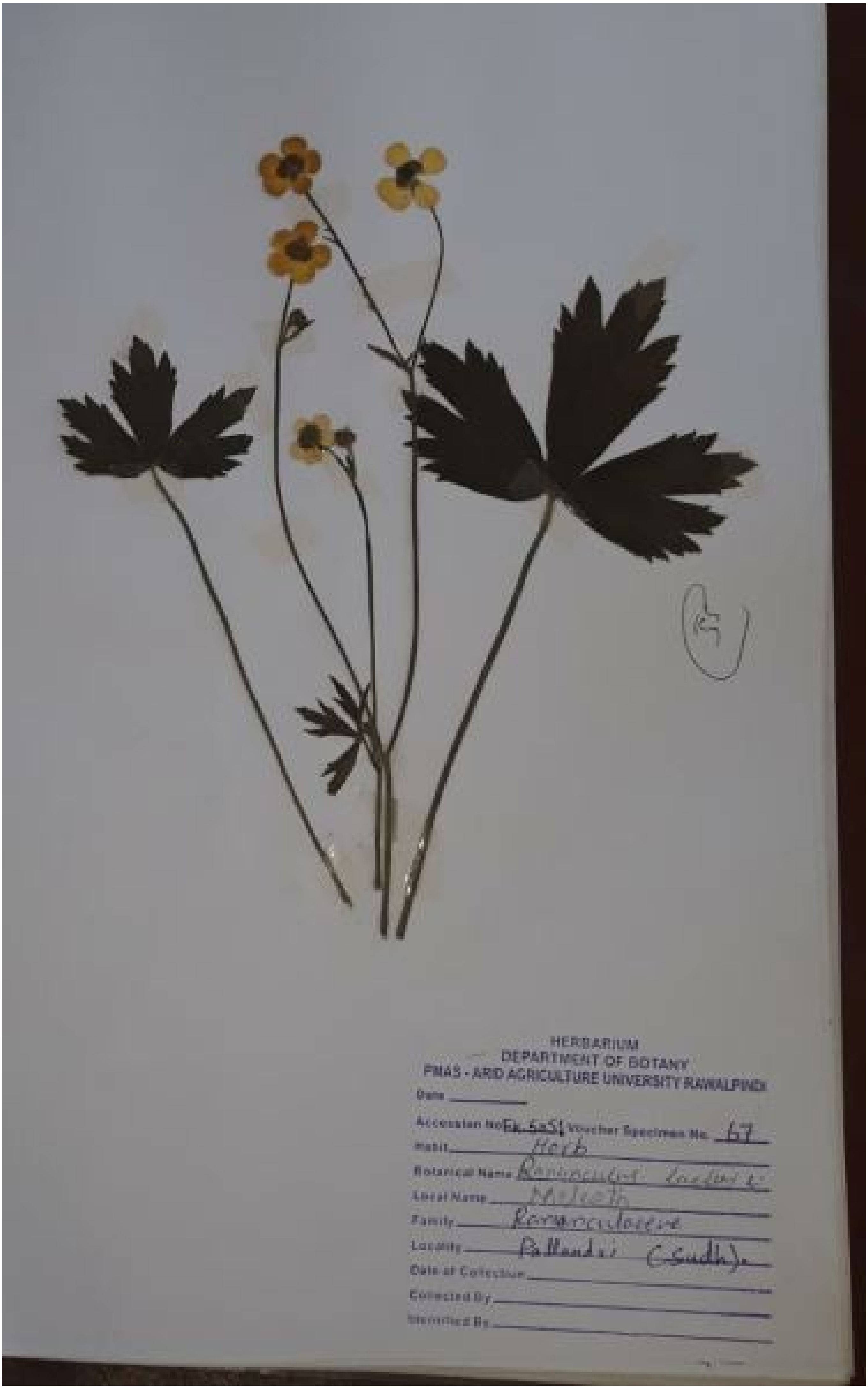
HERBARIUM
DEPARTMENT OF BOTANY
PMAS - ARID AGRICULTURE UNIVERSITY RAWALPINDI
Date
Accession No. Voucher Specimen No. 67
Habit Herb
Botanical Name
Local Name
Family
Locality
Date of Collection
Collected By
Identified By

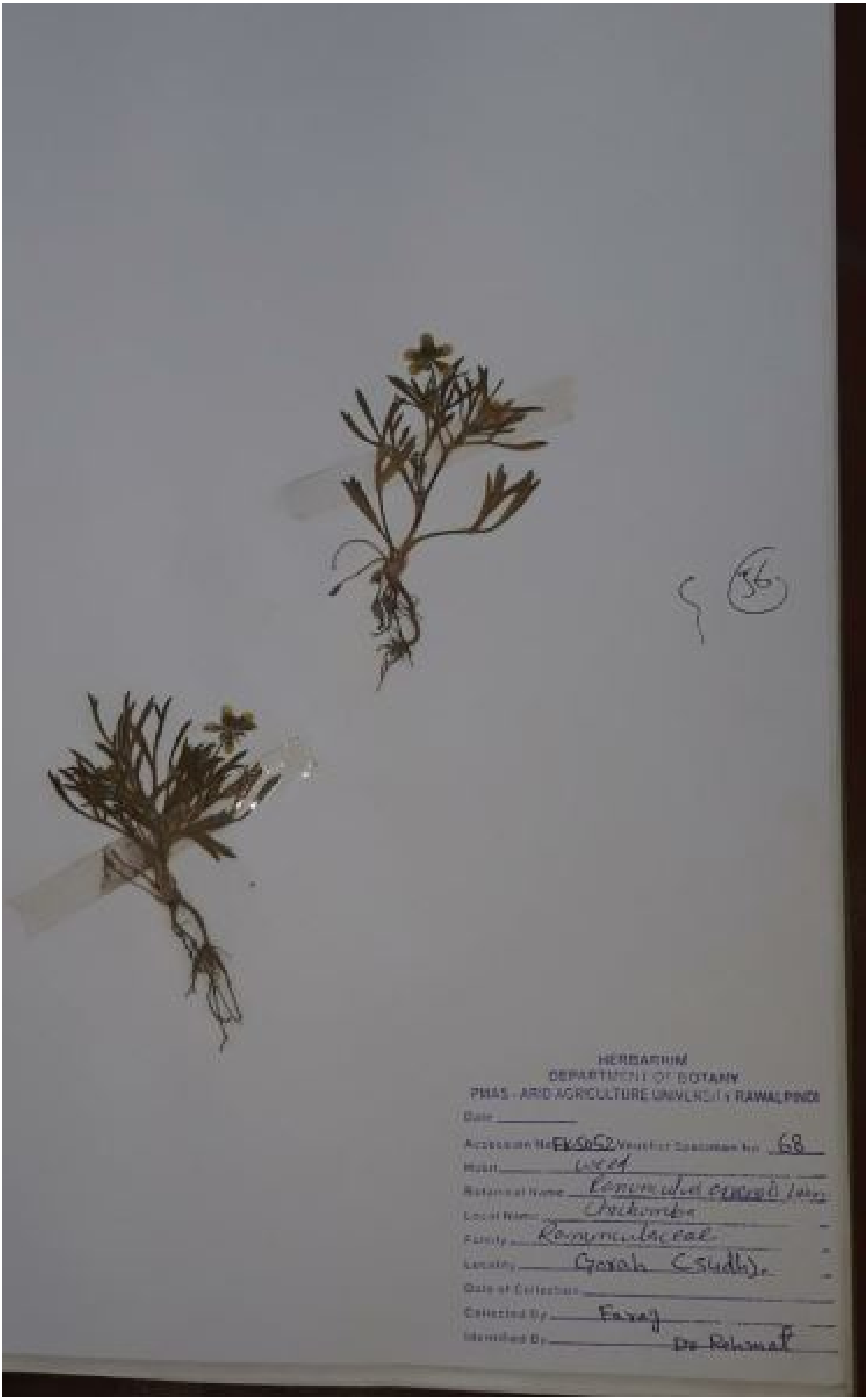
HERBARIUM
DEPARTMENT OF BOTANY
PMAS - ARID AGRICULTURE UNIVERSITY RAWALPINDI
Date
Accession No. FK5052 Voucher Specimen No. 68
Habit weed
Botanical Name
Local Name
Family Ranunculaceae
Locality Gorah
Date of Collection
Collected By Faraz
Identified By Dr Rehmat

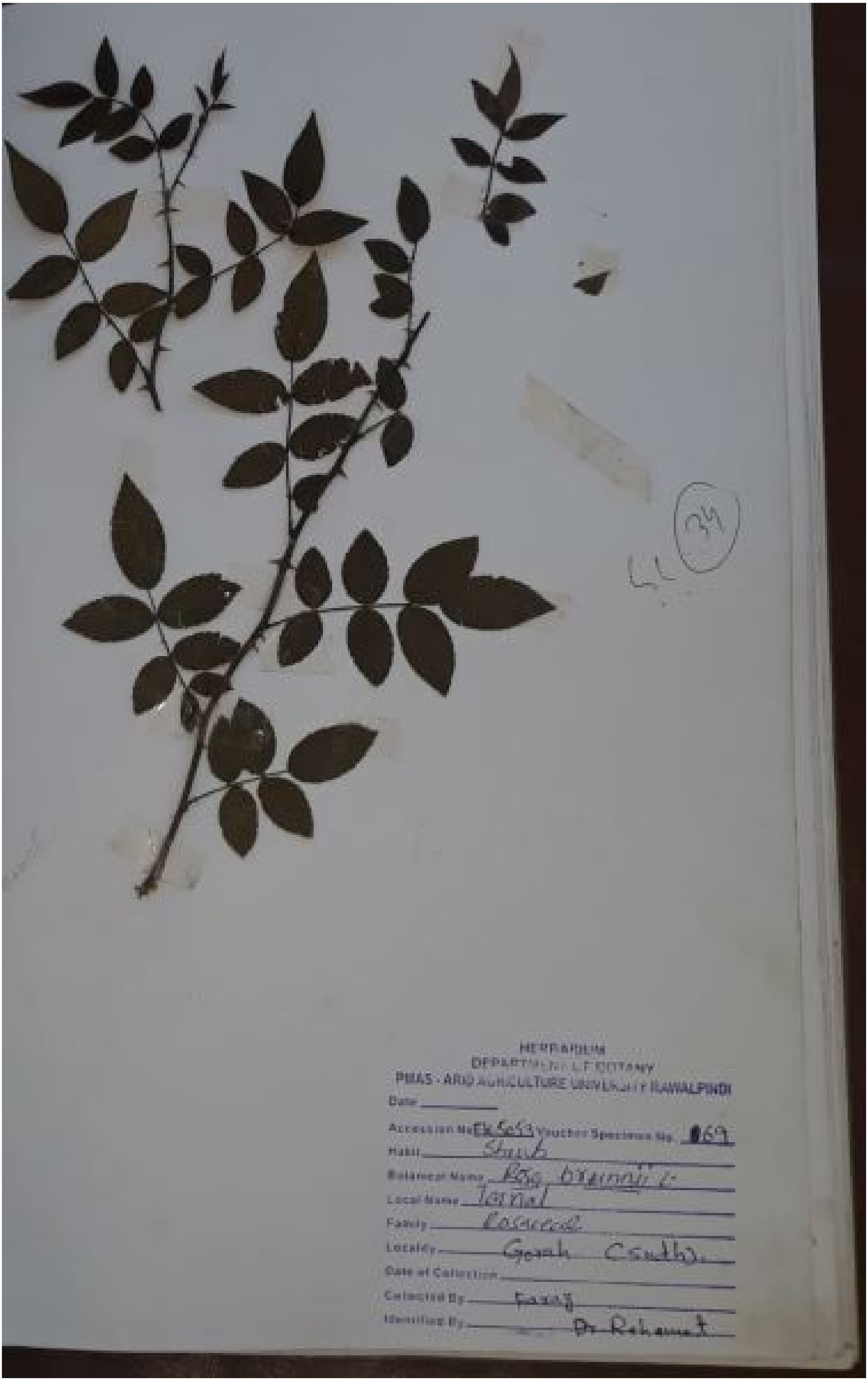
HERBARIUM
PMAS - ARID AGRICULTURE UNIVERSITY RAWALPINDI
Date
Voucher Specimen No.
Habit
Local Name
Family
Locality
Date of Collection
Collected By
Identified By

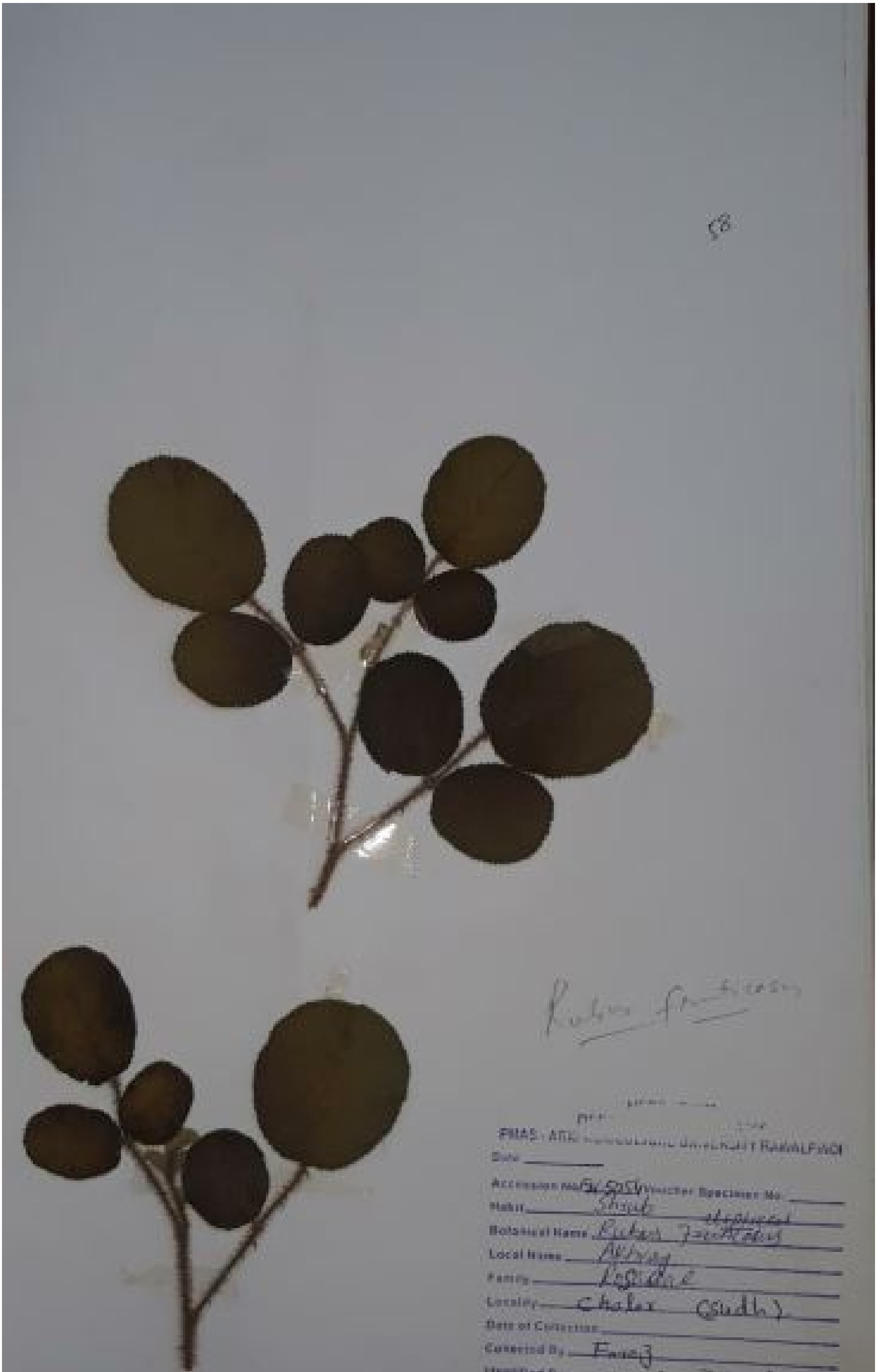

Rubus fruticosus
PMAS - ARID AGRICULTURE UNIVERSITY RAWALPINDI
Date
Accession No. Voucher Specimen No.
Habit Shrub
Botanical Name Rubus fruticosus
Local Name Akhray
Family Rosaceae
Locality Chakri (Sudh)
Date of Collection
Collected By Fawaz
Identified By Dr Rahmat

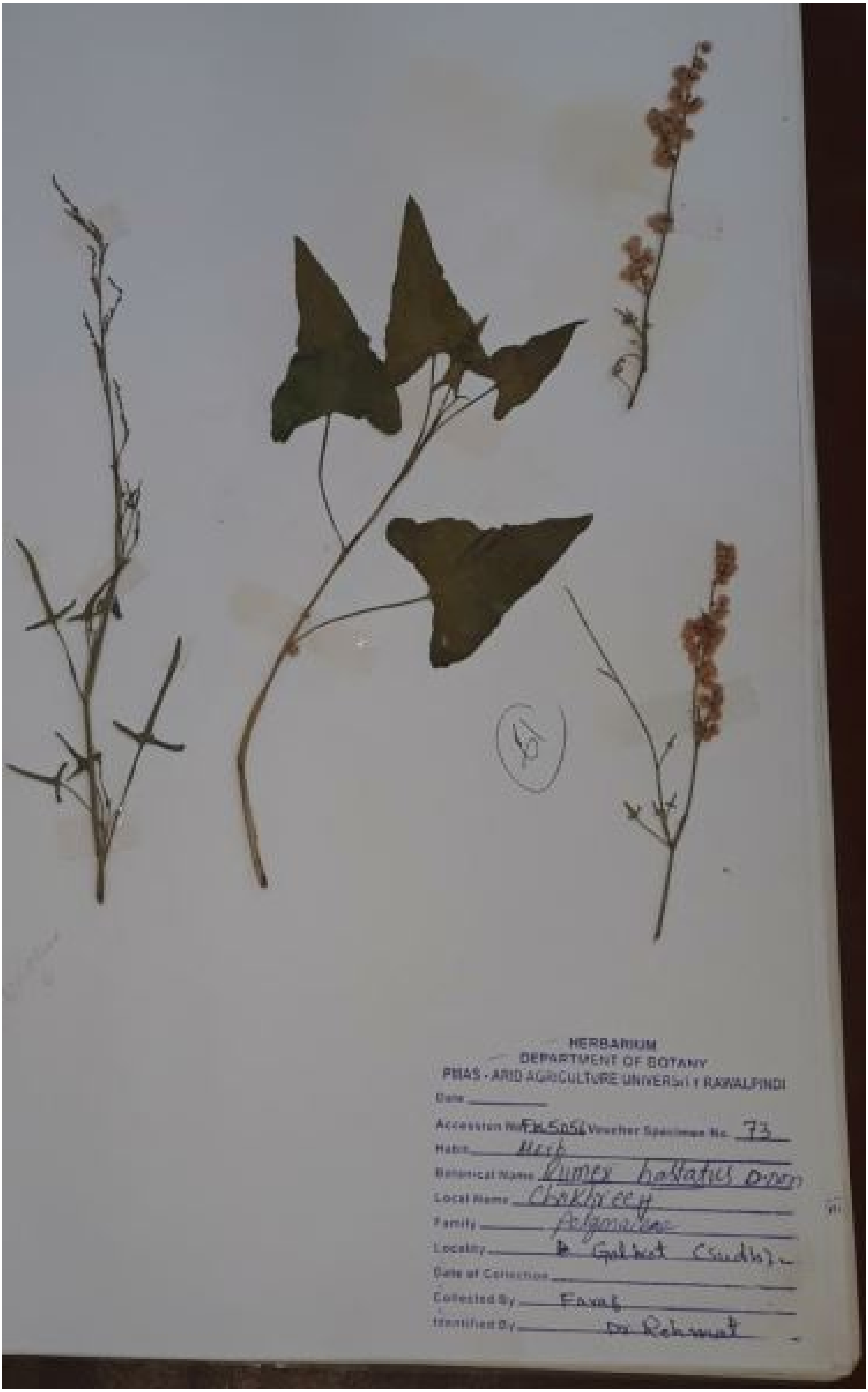
HERBARIUM
DEPARTMENT OF BOTANY
PMAS - ARID AGRICULTURE UNIVERSITY RAWALPINDI
Date
Accession No. Voucher Specimen No. 73
Habit Herb
Botanical Name Rumex hastatus
Local Name
Family
Locality
Date of Collection
Collected By
Identified By

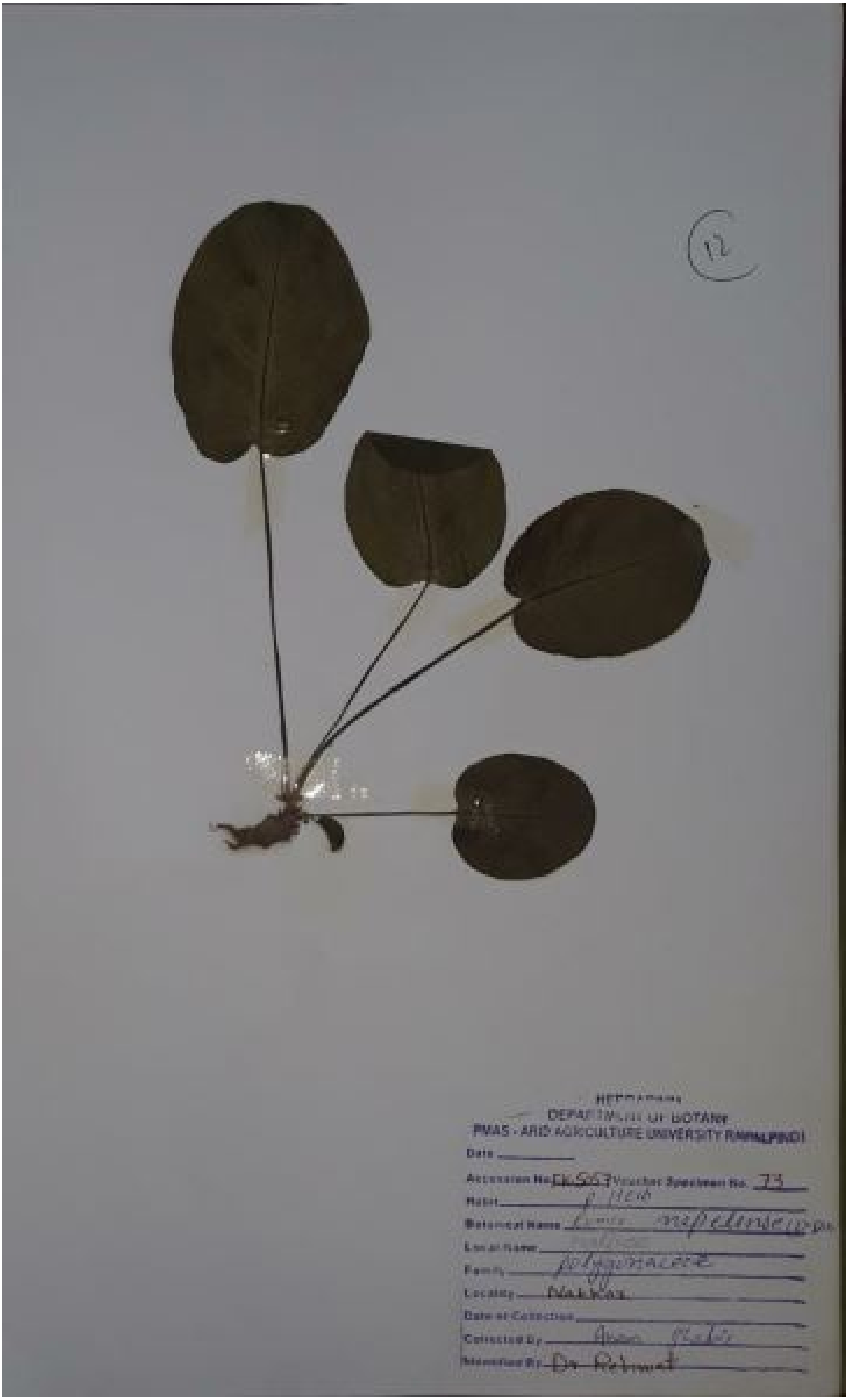
12
DEPARTMENT OF BOTANY
PMAS - ARID AGRICULTURE UNIVERSITY RAWALPINDI
Date
Accession No
Voucher Specimen No. 73
Habit
Botanical Name
Local Name
Family polygonaceae
Locality
Date of Collection
Collected By
Identified By

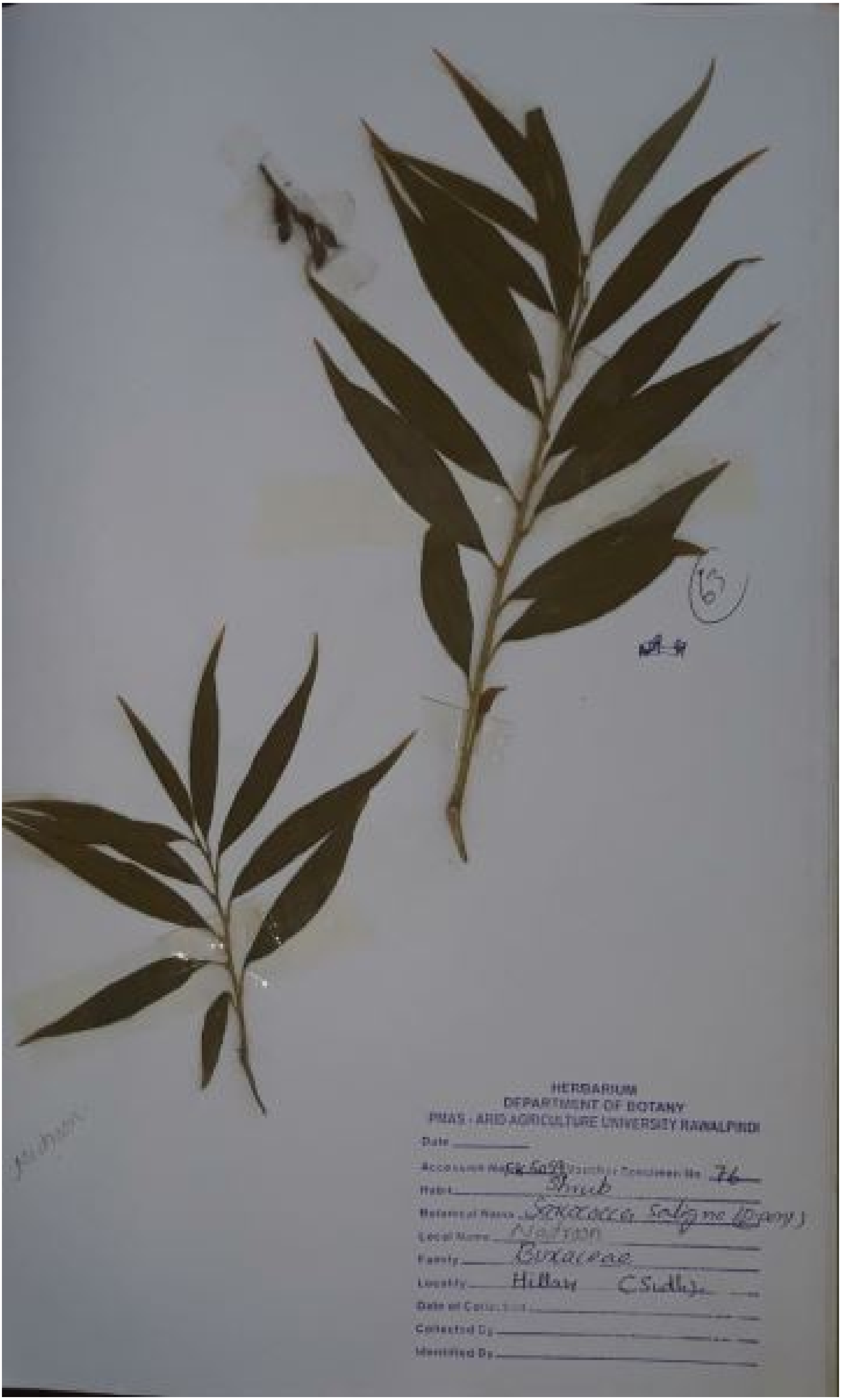
HERBARIUM
DEPARTMENT OF BOTANY
PMAS - ARID AGRICULTURE UNIVERSITY RAWALPINDI
Date
Accession No.
Specimen No. 76
Habit Shrub
Botanical Name
Local Name
Family Buxaceae
Locality
Date of Collection
Collected By
Identified By

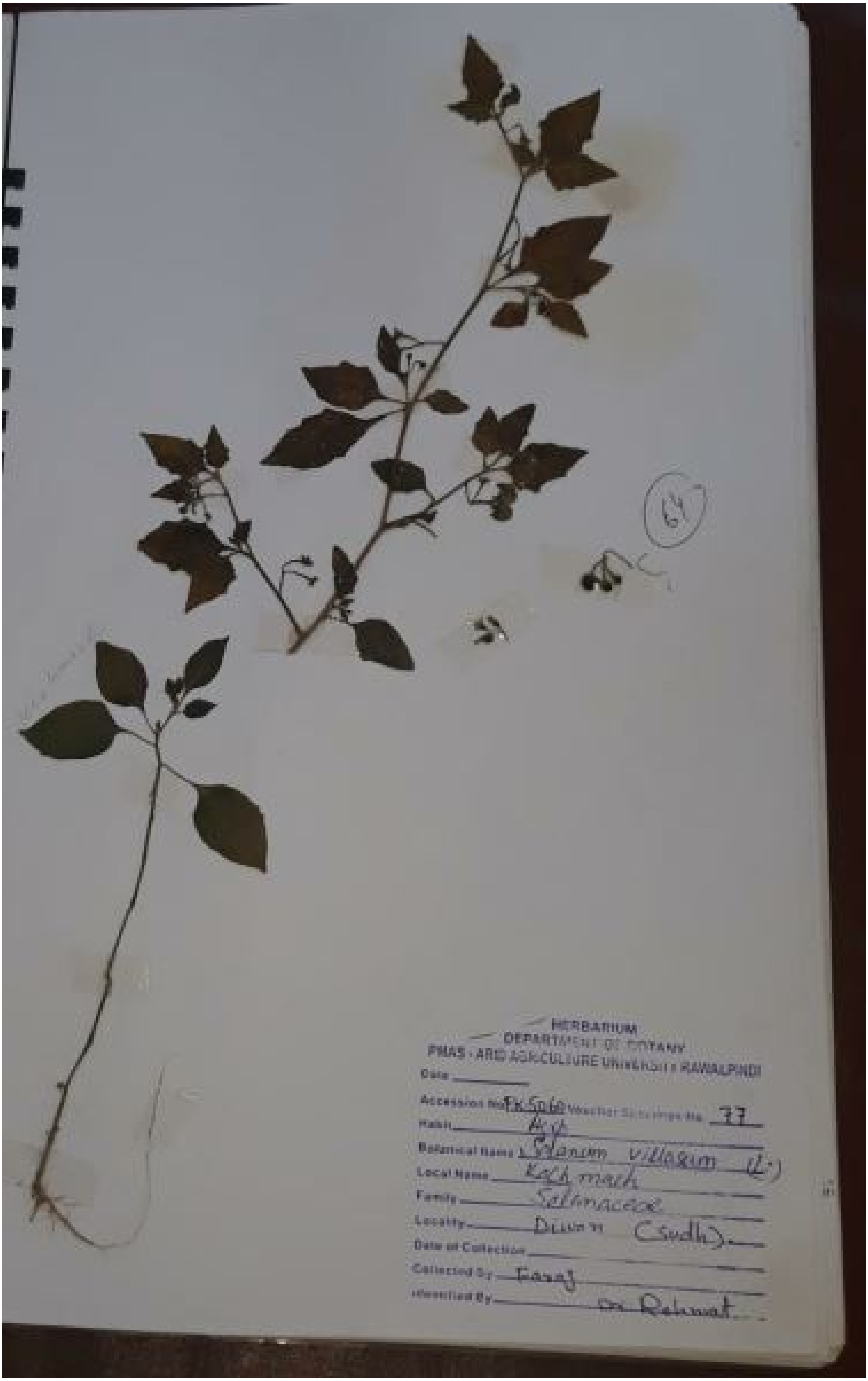

64
HERBARIUM
DEPARTMENT OF BOTANY
PMAS - ARID AGRICULTURE UNIVERSITY RAWALPINDI
Date
Accession No. PK 5060 [illegible] No. 77
Habit Hrb
Botanical Name Solanum villosum (L.)
Local Name Kach mach
Family Solanaceae
Locality Dluu 11 (Sudh)
Date of Collection
Collected By Faraz
Identified By Dr Rehmat

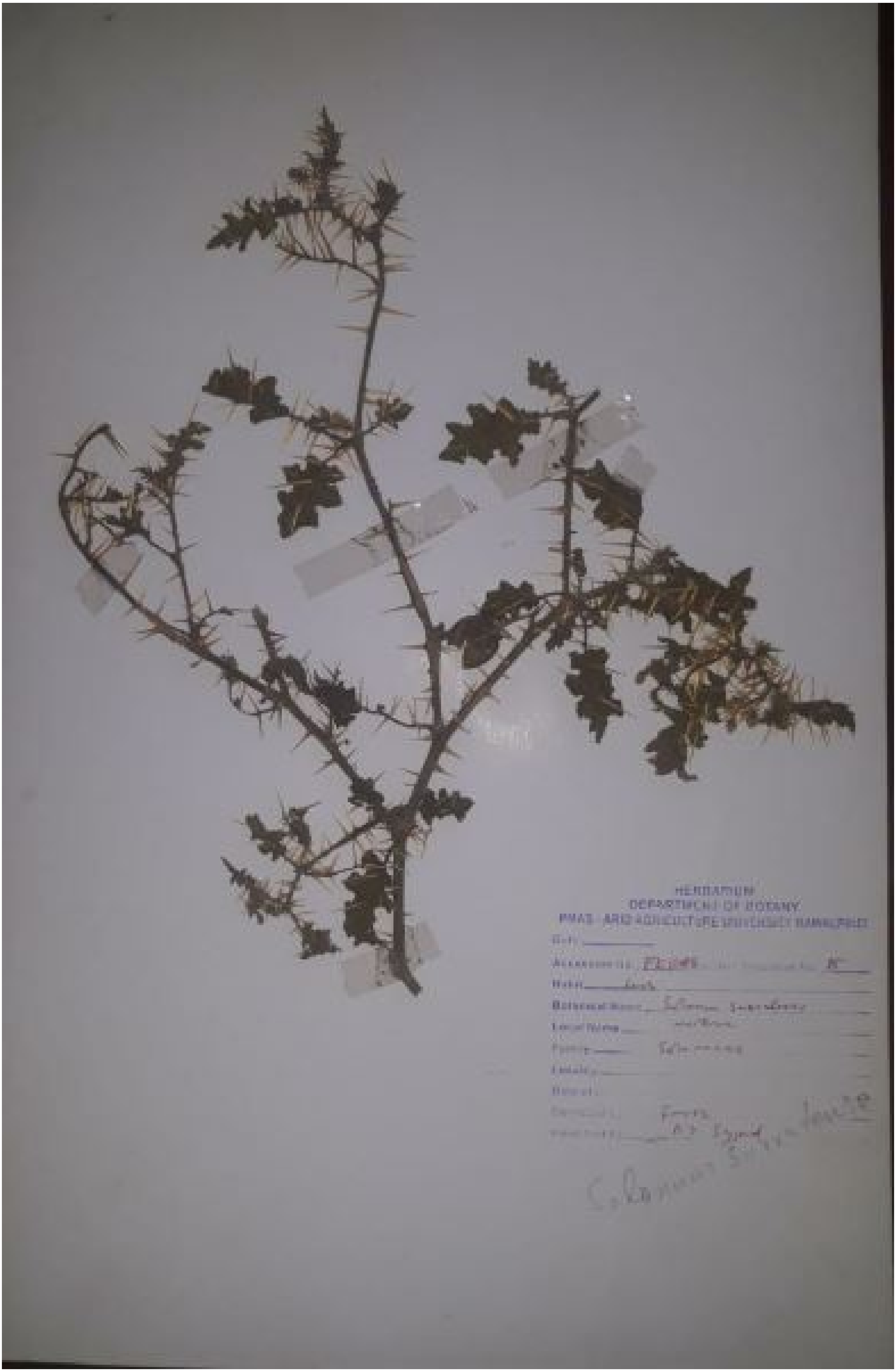
HERBARIUM
DEPARTMENT OF BOTANY
PMAS - ARID AGRICULTURE UNIVERSITY RAWALPINDI

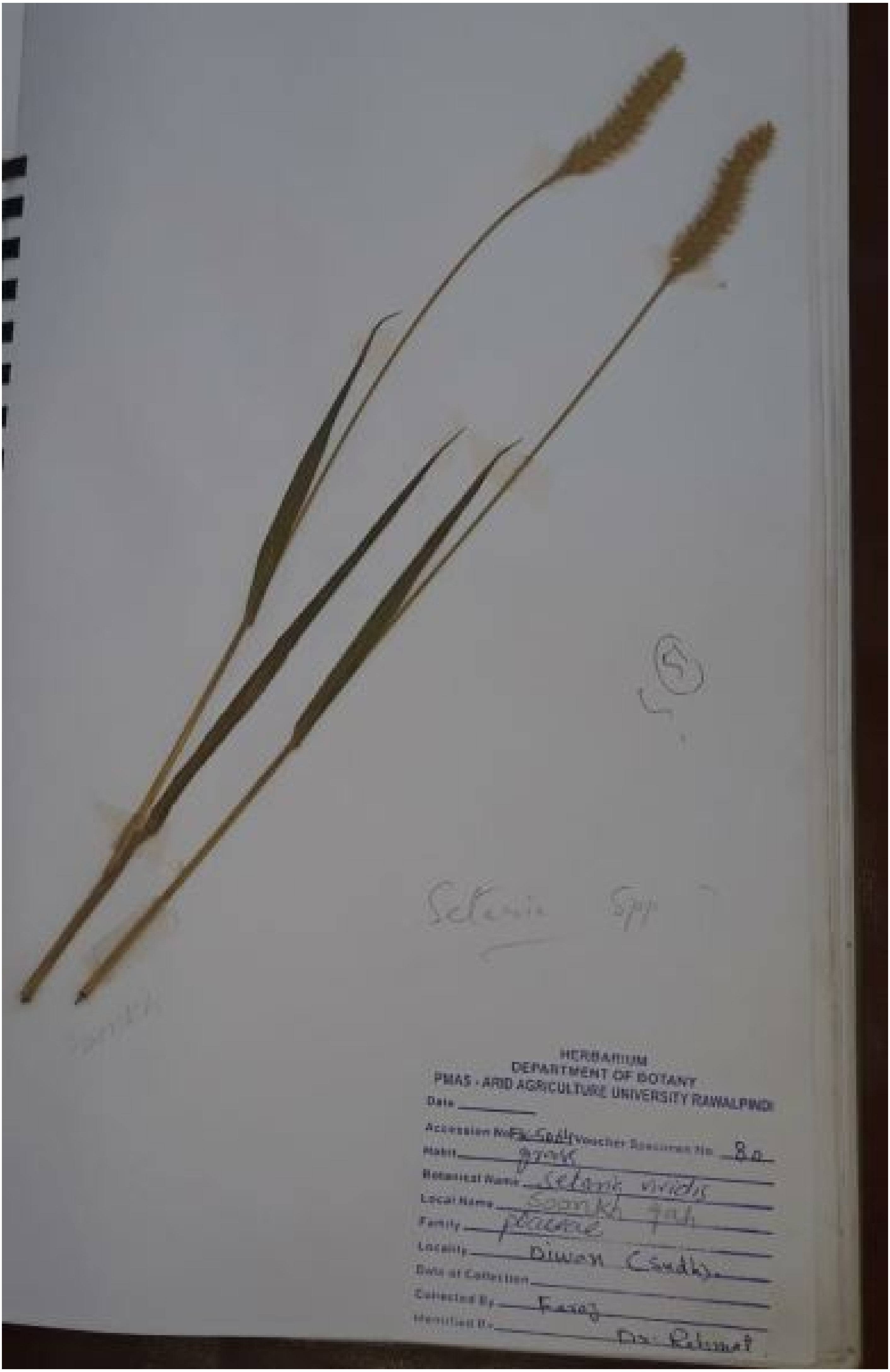
HERBARIUM
DEPARTMENT OF BOTANY
PMAS - ARID AGRICULTURE UNIVERSITY RAWALPINDI
Date
Accession No.
Voucher Specimen No.
Habit
Botanical Name
Local Name
Family
Locality
Date of Collection
Collected By
Identified By

EMORY UNIVERSITY
022133
HERBARIUM

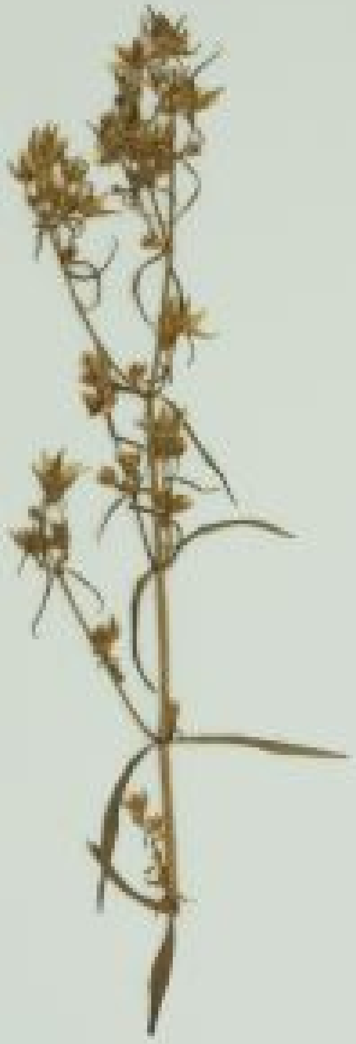

INCORPORATED INTO THE
MEDICAL BOTANY SPECIAL COLLECTION

FK 103

Michelle Lin 25-September-2017

## EMORY UNIVERSITY HERBARIUM

FLORA OF ISLAMIC REPUBLIC OF PAKISTAN

*Swertia chirata* Buch.-Ham. ex C.B. Clarke GENTIANACEAE
"*Jifwero*"

Dhir Dhrach, Sudhanoti, Azad Kashmir, Islamic Republic of Pakistan

wild herb, abundant

whitish yellow flowers, bitter in taste

roots have cooling effect used to treat diabetes and liver problems
local name: Chrirata

Muhammad Faraz Khan FK-103 10 October 2017

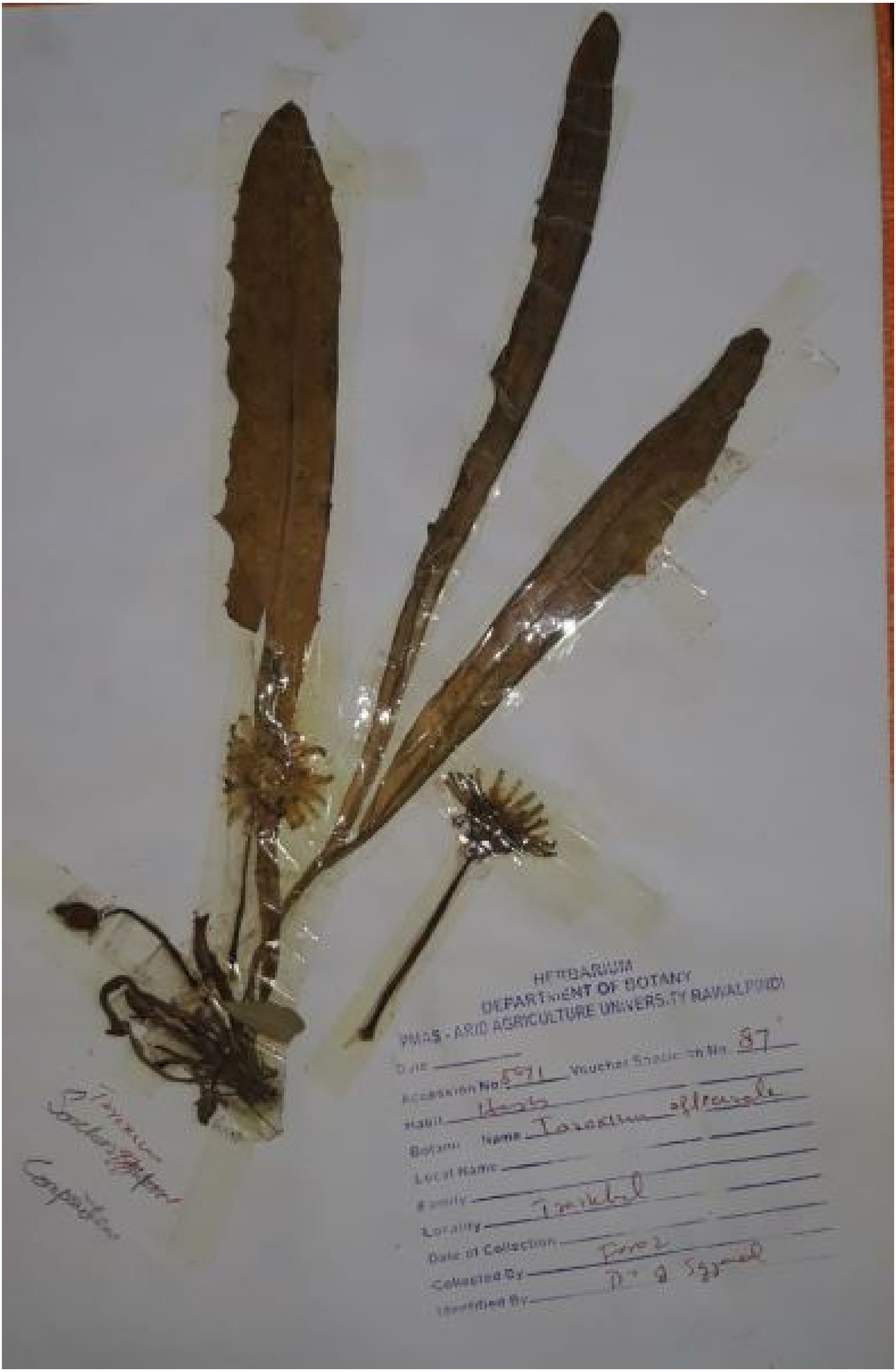
HERBARIUM
DEPARTMENT OF BOTANY
PMAS - ARID AGRICULTURE UNIVERSITY RAWALPINDI
Date
Accession No.
Voucher Specimen No. 87
Habit
Botanical Name Taraxacum officinale
Local Name
Family
Locality
Date of Collection
Collected By
Identified By
Compositae

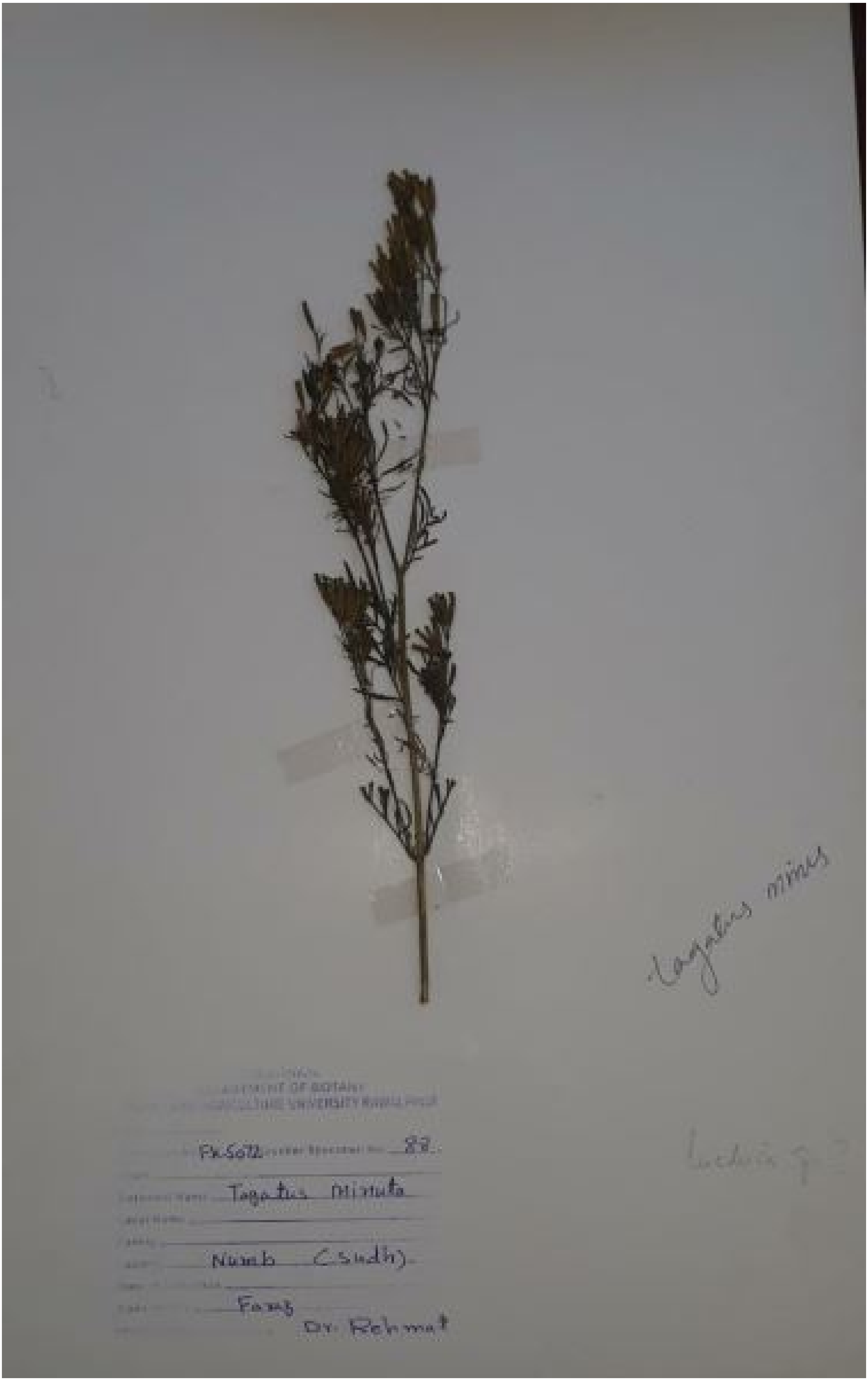
88
Tagatis minuta
Numb (Sudh)
Faraz
Dr. Rehmat

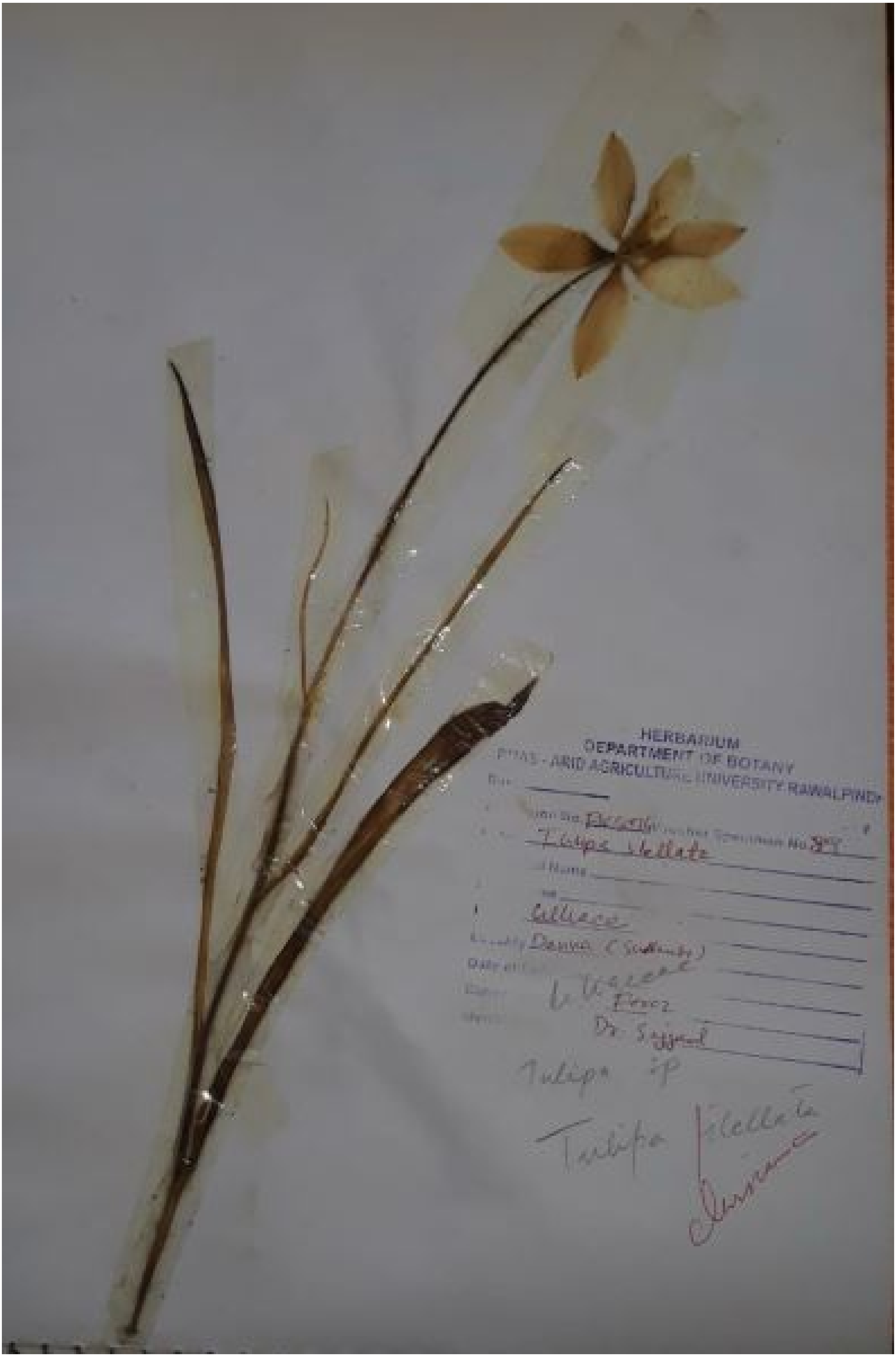
HERBARIUM
DEPARTMENT OF BOTANY
ARID AGRICULTURE UNIVERSITY RAWALPINDI
Liliaceae
Tulipa sp
Tulipa stellata

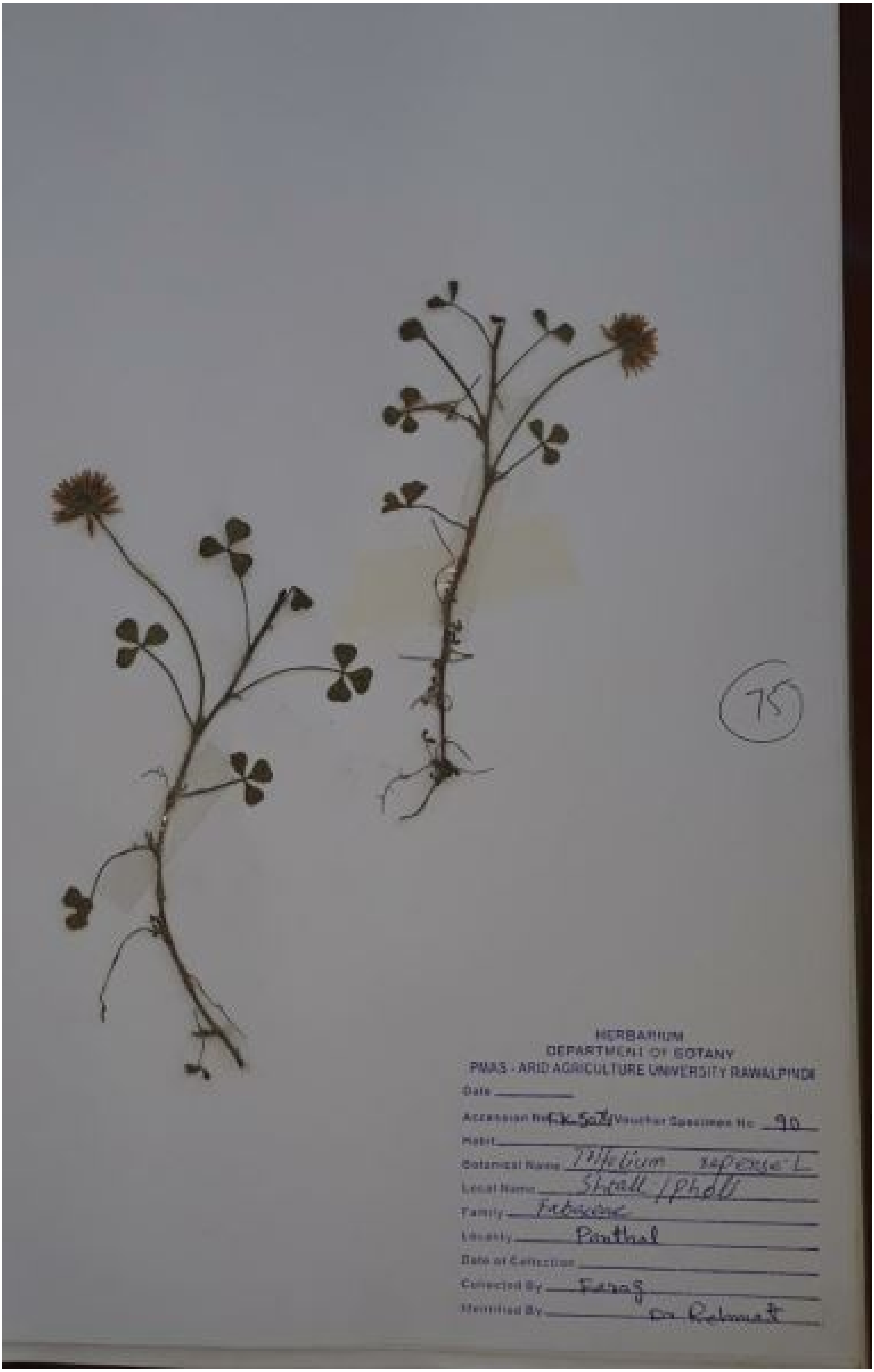
75
HERBARIUM
DEPARTMENT OF BOTANY
PMAS - ARID AGRICULTURE UNIVERSITY RAWALPINDI
Date
Accession No.
Voucher Specimen No. 90
Habit
Botanical Name
Local Name
Family
Locality
Date of Collection
Collected By
Identified By

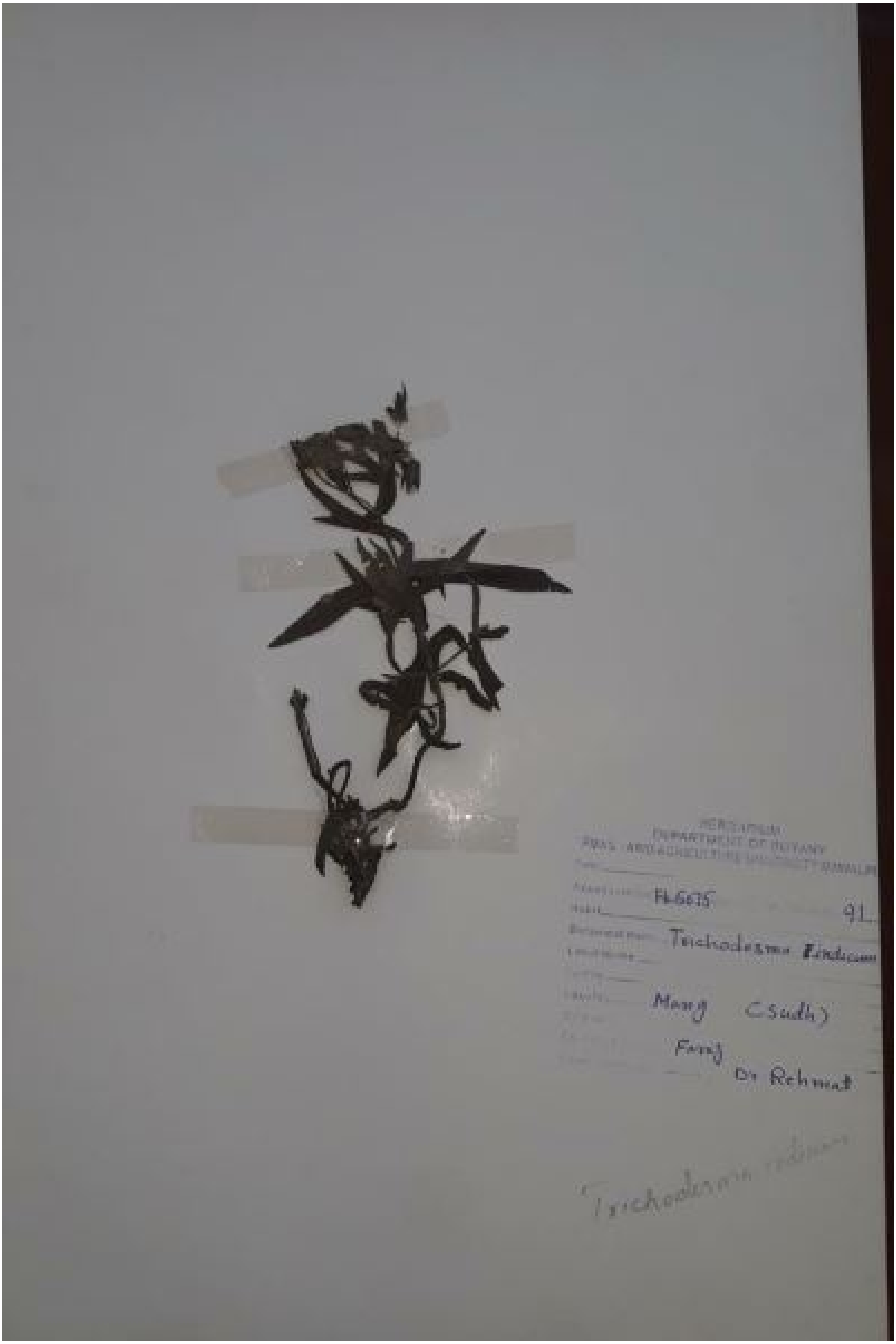

DEPARTMENT OF BOTANY
91
Trichodesma
Dr Rehmat

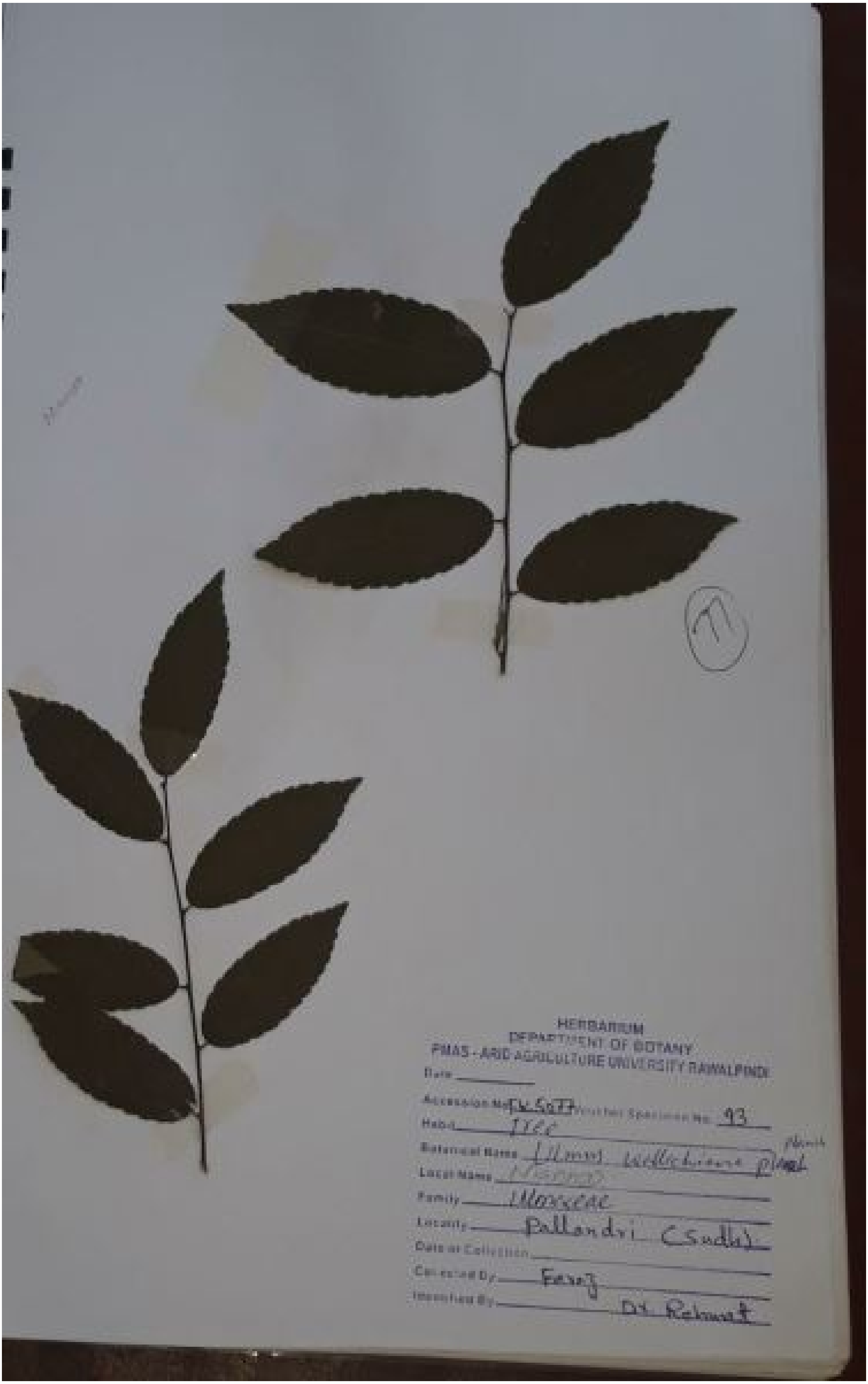
HERBARIUM
DEPARTMENT OF BOTANY
PMAS - ARID AGRICULTURE UNIVERSITY RAWALPINDI
Date
Accession No.
Specimen No. 93
Habit
Botanical Name
Local Name
Family
Locality Pallandri
Date of Collection
Collected By
Identified By Dr. Rehmat

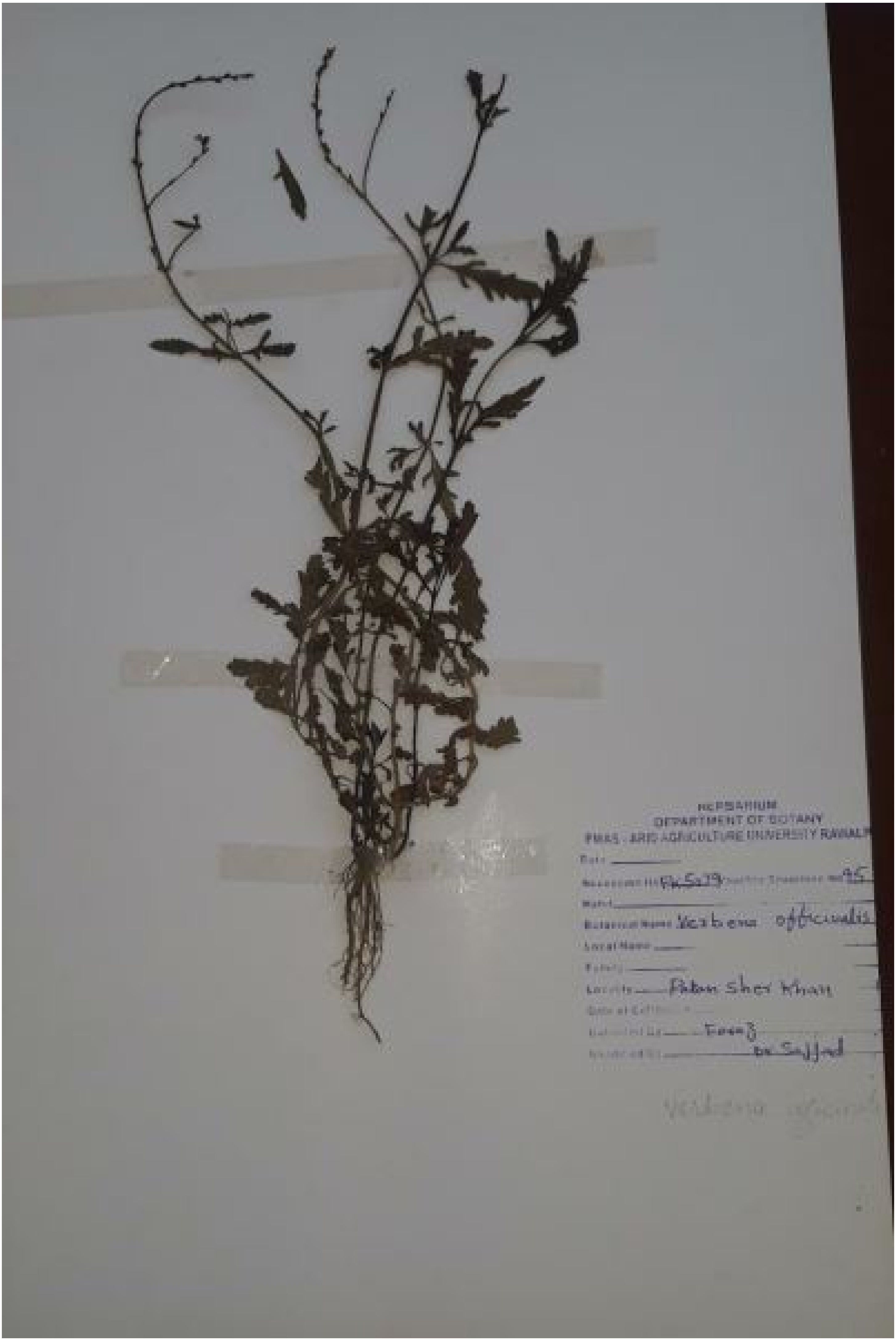
HERBARIUM
DEPARTMENT OF BOTANY
Pakan Sher Khan

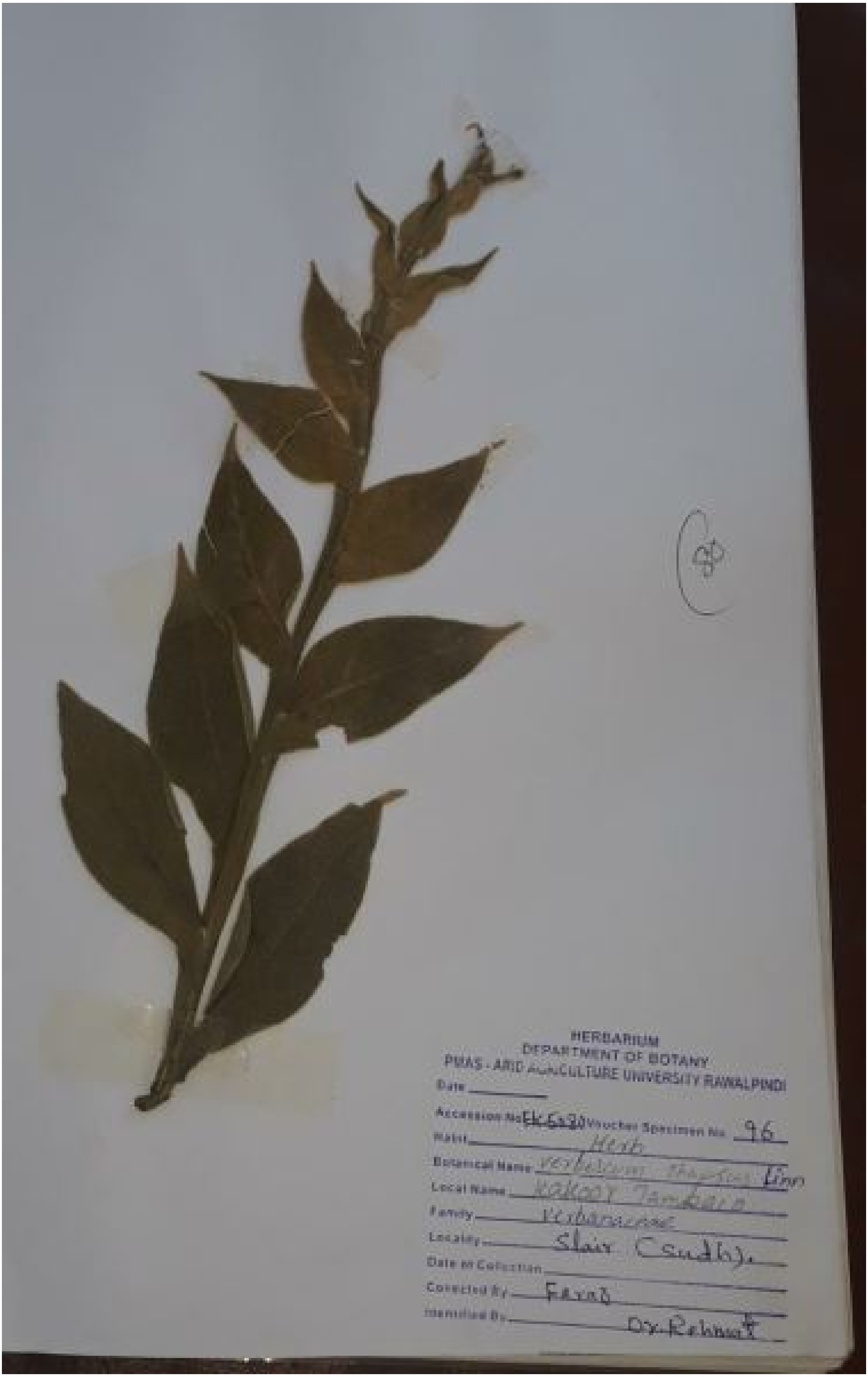

HERBARIUM
DEPARTMENT OF BOTANY
PMAS - ARID AGRICULTURE UNIVERSITY RAWALPINDI
Date
Accession No. Voucher Specimen No. 96
Habit Herb
Botanical Name
Local Name
Family
Locality
Date of Collection
Collected By Faraz
Identified By

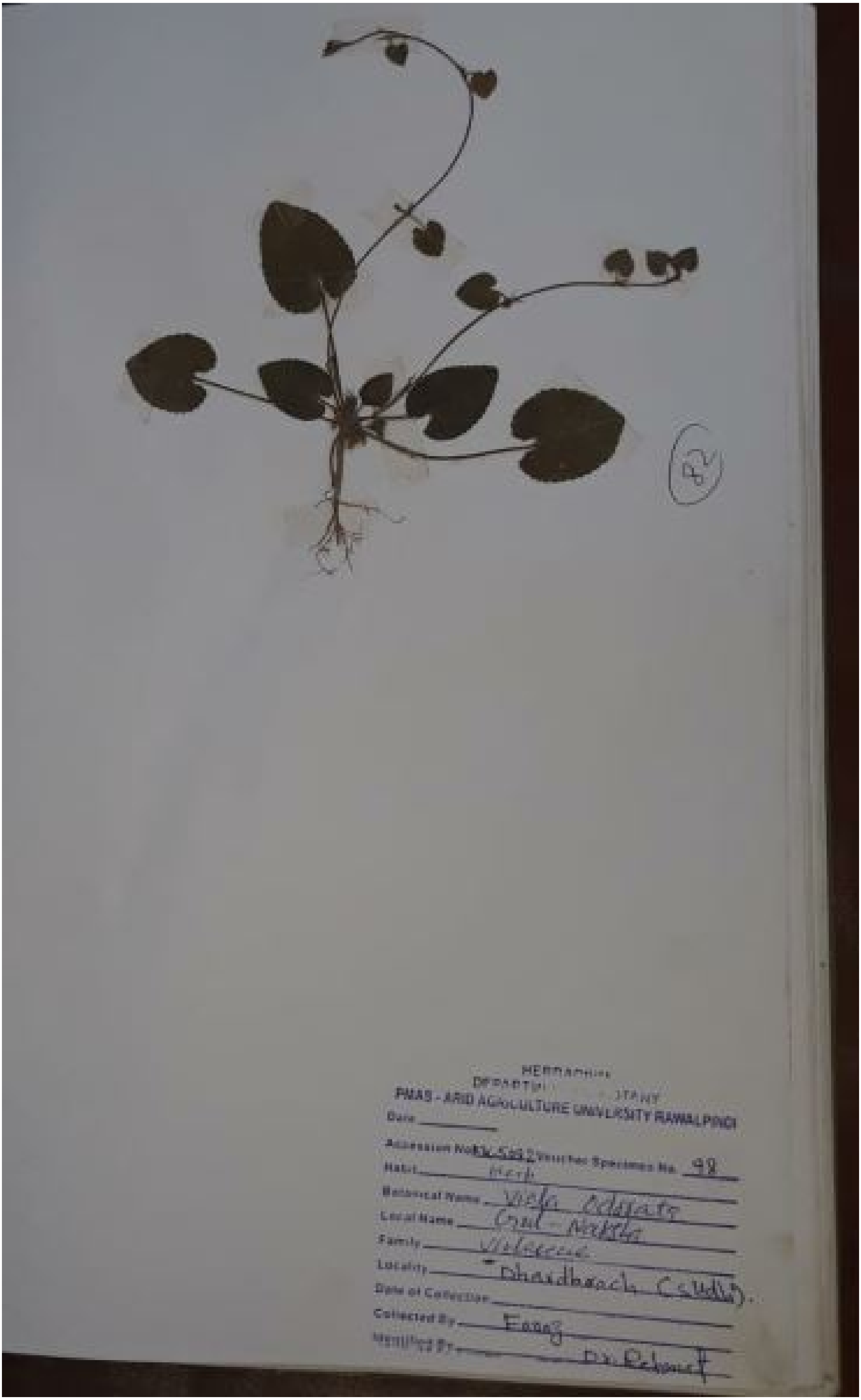
PMAS - ARID AGRICULTURE UNIVERSITY RAWALPINDI
Date
Accession No.
Voucher Specimen No.
Habit
Herb
Botanical Name
Local Name
Family
Locality
Date of Collection
Collected By
Identified By

EMORY UNIVERSITY
024201
HERBARIUM

INCORPORATED INTO THE
MEDICAL BOTANY SPECIAL COLLECTION

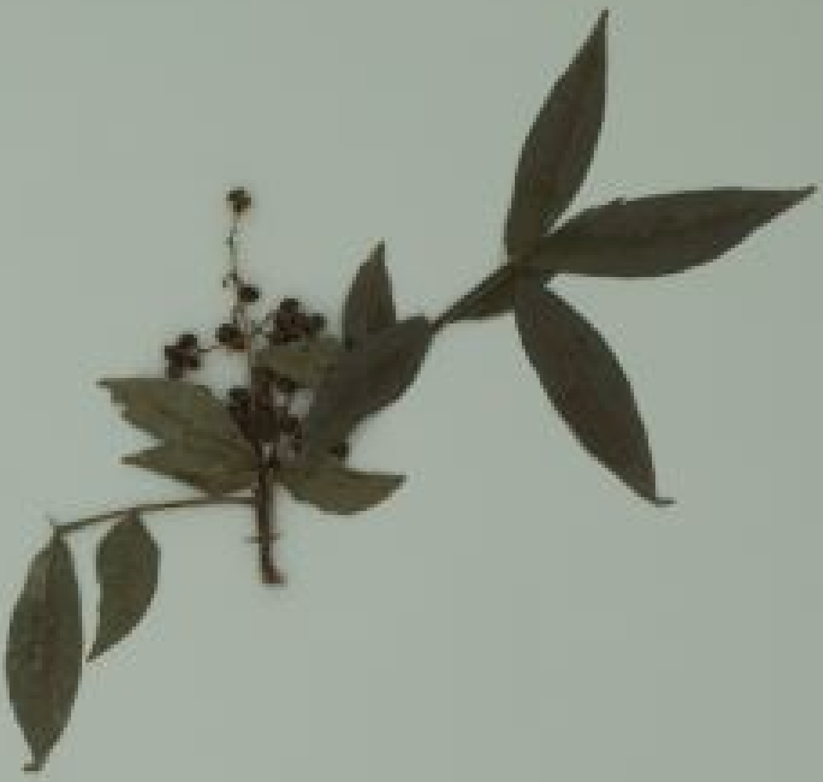

FK-73

[illegible]
20-Sept-[illegible]

**EMORY UNIVERSITY HERBARIUM**
FLORA OF ISLAMIC REPUBLIC OF PAKISTAN

***Zanthoxylum armatum*** DC. RUTACEAE
***"winged prickly ash"***

Baral, Palandri, Azad Kashmir, Islamic Republic of Pakistan.

distributed in Rangelands dry areas; common

shrub, flowers less conspicuous, prominent seed, thorns on the stem

local name: timber

Muhammad Faraz Khan FK-73 14 April 2016

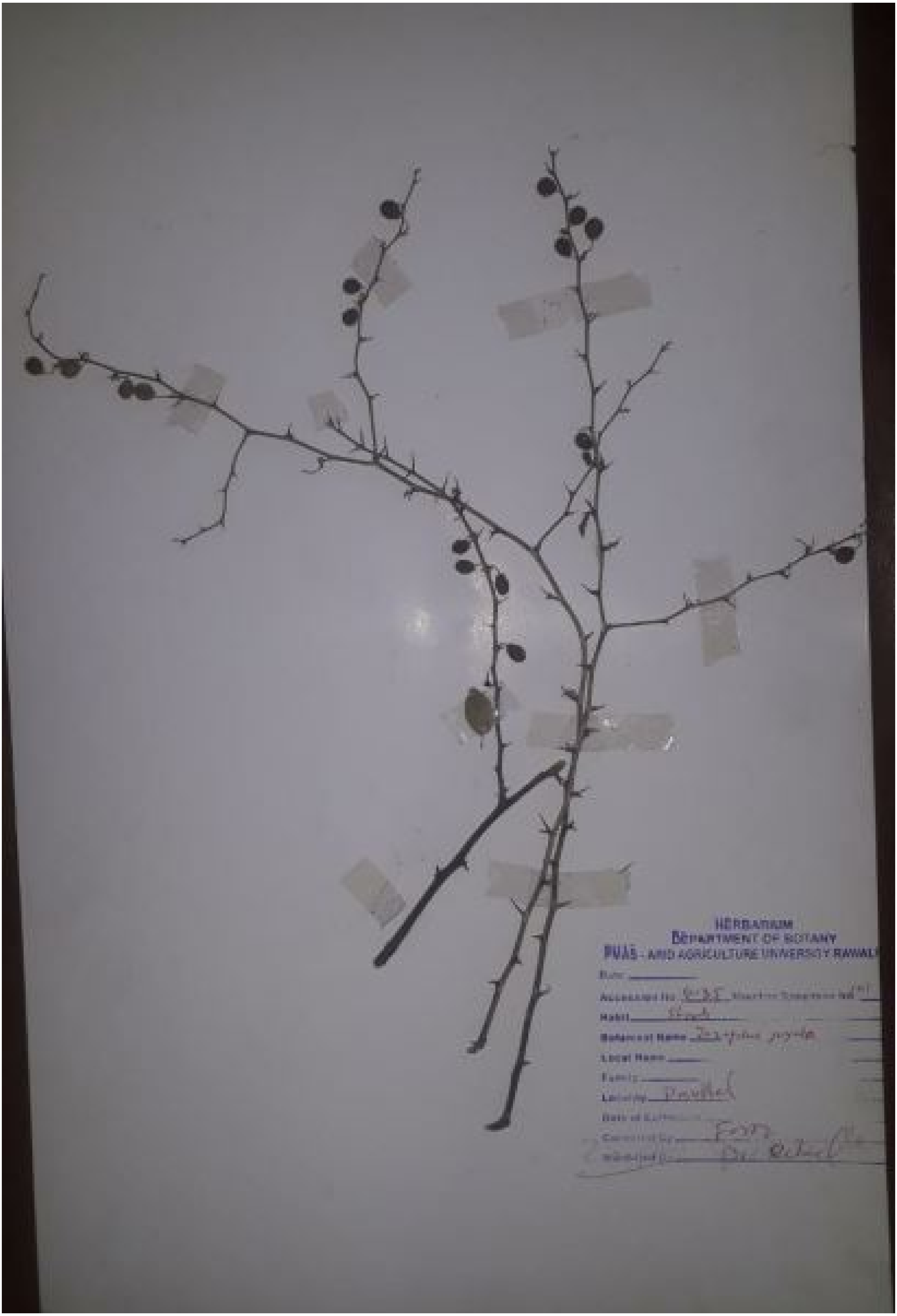
HERBARIUM
DEPARTMENT OF BOTANY
PMAS - ARID AGRICULTURE UNIVERSITY RAWAL
Habit
Botanical Name
Local Name
Family
Locality
